# A Cytotoxic Bis(1,2,3‐triazol‐5‐ylidene)carbazolide Gold(III) Complex Targets DNA by Partial Intercalation

**DOI:** 10.1002/chem.202100598

**Published:** 2021-05-17

**Authors:** Danielle van der Westhuizen, Cathryn A. Slabber, Manuel A. Fernandes, Daniël F. Joubert, George Kleinhans, C. Johan van der Westhuizen, André Stander, Orde Q. Munro, Daniela I. Bezuidenhout

**Affiliations:** ^1^ Molecular Sciences Institute School of Chemistry University of the Witwatersrand 2050 Johannesburg South Africa; ^2^ Department of Physiology University of Pretoria 0031 Pretoria South Africa; ^3^ Chemistry Department University of Pretoria 0028 Pretoria South Africa; ^4^ Future Production: Chemicals Pharmaceutical Technologies Research Group Council for Scientific and Industrial Research (CSIR) 0184 Pretoria South Africa; ^5^ Laboratory of Inorganic Chemistry Environmental and Chemical Engineering University of Oulu 3000 Oulu Finland

**Keywords:** anticancer, cytotoxicity, gold complexes, mesoionic carbenes, metallodrugs

## Abstract

The syntheses of bis(triazolium)carbazole precursors and their corresponding coinage metal (Au, Ag) complexes are reported. For alkylated triazolium salts, di‐ or tetranuclear complexes with bridging ligands were isolated, while the bis(aryl) analogue afforded a bis(carbene) Au^I^‐CNC pincer complex suitable for oxidation to the redox‐stable [Au^III^(CNC)Cl]^+^ cation. Although the ligand salt and the [Au^III^(CNC)Cl]^+^ complex were both notably cytotoxic toward the breast cancer cell line MDA‐MB‐231, the Au^III^ complex was somewhat more selective. Electrophoresis, viscometry, UV‐vis, CD and LD spectroscopy suggest the cytotoxic [Au^III^(CNC)Cl]^+^ complex behaves as a partial DNA intercalator. In silico screening indicated that the [Au^III^(CNC)Cl]^+^ complex can target DNA three‐way junctions with good specificity, several other regular *B*‐DNA forms, and *Z*‐DNA. Multiple hydrophobic π‐type interactions involving T and A bases appear to be important for *B*‐form DNA binding, while phosphate O⋅⋅⋅Au interactions evidently underpin *Z*‐DNA binding. The CNC ligand effectively stabilizes the Au^III^ ion, preventing reduction in the presence of glutathione. Both the redox stability and DNA affinity of the hit compound might be key factors underpinning its cytotoxicity in vitro.

## Graphical abstract

**Towards new Au^III^ anticancer agents**. Herein a redox‐stabilised DNA‐targeting Au^III^ bis(carbene) pincer complex with significant cytotoxicity is reported that is likely underpinned by partial DNA intercalation. In silico studies indicate that the complex can also target unusual DNA forms such as 3‐way junctions and *Z*‐DNA. The structures of several multinuclear Ag^I^ complexes discovered en route to the target Au^III^ complex were also elucidated.

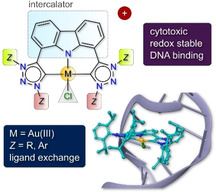

## Introduction

The wide range of coordination numbers, geometries, ligands, and redox states available for metal complexes provide chemists with an impressive array of molecular tools to target one, or several, biological macromolecules. A topical objective is to design metallodrugs for multi‐faceted chemotherapeutic approaches.[^1^, ^2^, ^3^, ^4^, ^5^, ^6^, ^7^, ^8^, ^9^, ^10^] Gold complexes, irrespective of their oxidation state, have emerged as effective anticancer agents due to their ability to target DNA and/or several intracellular proteins.[^11^, ^12^, ^13^, ^14^, ^15^, ^16^, ^17^, ^18^, ^19^, ^20^, ^21^, ^22^, ^23^, ^24^, ^25^] Importantly, stabilization of the Au^III^ ion by an appropriate multidentate ligand scaffold, for instance porphyrin/pyrrole[^26^, ^27^] or CNC type pincer ligands,[^28^, ^29^, ^30^, ^31^, ^32^, ^33^, ^34^, ^35^, ^36^, ^37^, ^38^, ^39^, ^40^] results in Au^III^ complexes that are remarkably stable when exposed to intracellular reductants. The complexes also elicit strong cytotoxicity against cancer cells and can bind to DNA noncovalently via intercalation. Enhancing the selectivity of Au^III^ pincer complexes towards cancer cells remains a formidable challenge but is improved using non‐toxic ligands such as NHCs (N‐heterocyclic carbenes), which are renowned strong σ‐donors that effectively stabilize both Au^I^ and Au^III^ under physiological conditions.[^41^, ^42^, ^43^, ^44^, ^45^, ^46^, ^47^, ^48^, ^49^, ^50^, ^51^, ^52^] For metallodrug design, 1,2,3‐triazol‐5‐ylidene (trz) ligands, which are a subclass of NHCs, offer facile modulation, high functional group tolerance, and stabilization of metals in both high‐ and low oxidation states.[^53^, ^54^, ^55^, ^56^, ^57^, ^58^] Surprisingly, few reports address the anticancer potential of trz‐based compounds.[^59^, ^60^, ^61^, ^62^]

We previously reported that a monoanionic CNC pincer ligand with a carbazole backbone and two flanking trz groups successfully stabilized reactive first row late transition metals.^[63]^ Notably, the gold derivative could be oxidized to robust Au^III^ complexes.[^64^, ^65^] The enhanced redox stability and the planar aromatic carbazole moiety present ideal attributes to be explored further for DNA binding and cytotoxicity towards cancer cells. Indeed, carbazoles are often used as structural motifs in biologically relevant compounds that have antitumor properties. The rigid, planar tricyclic structure allows for DNA intercalation and the inhibition of DNA topoisomerases.[^66^, ^67^] Herein, we report the synthesis of modified triazolium precursor salts and their corresponding Au^I^ and Au^III^ complexes prepared by replacement of the bulky 2,6‐diisopropylphenyl (Dipp) wingtip groups with alkyl groups, or removal of the *tert‐*butyl groups on the carbazole spacer. Our hypothesis is that the diminished steric hindrance of the target cationic gold complexes could facilitate DNA binding via intercalation and thus engender significant, exploitable cytotoxicity for the complexes.

## Results and Discussion

### Synthesis and characterization of N1‐aryl‐N3‐alkyl‐1,2,3‐triazol‐5‐ylidene metal complexes

**Scheme 1:**
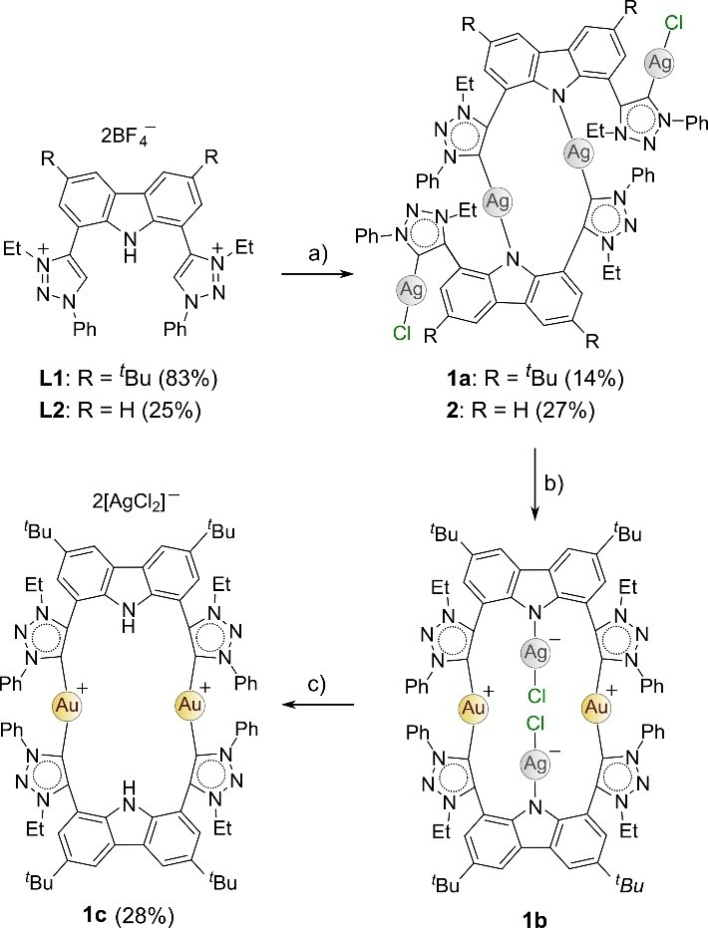
Synthesis of the bis(triazolylidene) complexes **1 b** and **1 c** via a transmetallation route starting from the triazolium salt **L1**. The tetranuclear analogues of the monocarbene silver(I) complexes **1 a** and **2** were also prepared from their respective triazolium salts, **L1** and **L2** (see text). The reaction conditions were as follows: a) 4 eq Ag_2_O, 10 eq KCl, CH_2_Cl_2_, rt, 7d. b) 1.5 eq Au(tht)Cl (tht=tetrahydrothiophene), 1 d. c) 7 days in solution.

**Scheme 2:**
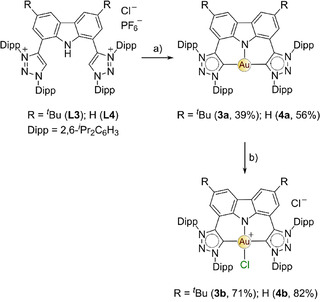
Metalation of **L3** and **L4** to form the Au^I^ CNC pincer complexes **3 a** and **4 a**, followed by oxidation to the corresponding Au^III^ complexes, **3 b** and **4 b**. The reaction conditions were as follows: a) 5 eq KN(Si(CH_3_)_3_)_2_, 1.5 eq Au(tht)Cl, THF, −60 °C, 3 d. b) 1.1 eq PhICl_2_, CH_2_Cl_2_, rt, 10 min–3 h.

The N3‐alkylated analogues of **L3** (**L1** and **L2**, Scheme 1) were prepared starting from the commercially available 9*H*‐carbazole precursor using modified literature procedures,[^68^, ^69^, ^70^, ^71^, ^72^] allowing for the synthesis of the targeted 1,4‐disubstituted‐1,2,3‐triazoles, **P1** and **P2** (Scheme S1, Supporting Information). Alkylation on both N3‐positions of the triazoles afforded the triazolium salts **L1** and **L2** as precursors for metalation with Au^I^ followed by oxidation to Au^III^. Initially, the metalation to Au^I^ was attempted by the *in situ* deprotonation of both ligand precursor salts **L1** and **L2** with 5 eq K[N(SiMe_3_)_2_] in the presence of 1.5 eq Au(tht)Cl (tht=tetrahydrothiophene) following the synthetic protocol previously reported for **3 a** (Scheme 2).^[64]^ Unfortunately for both **L1** and **L2**, the desired Au^I^ CNC pincer complexes were not obtained and no identifiable products could be isolated. Reactions in which we switched the base to KO^*t*^Bu, or the Au^III^ metal precursor to [^*n*^Bu_4_N][AuCl_4_] instead of Au(tht)Cl, were also unsuccessful, prompting the use of a transmetallation route.

The reaction of **L1** with 4 eq of Ag_2_O in excess KCl and 1.5 eq of Au(tht)Cl (step a followed by step b, without isolation of the intermediate **1 a**, Scheme 1) yielded a yellow solid. Analysis of the ^1^H NMR spectrum confirmed the disappearance of the two acidic triazolium (C5−H) and carbazole (N−H) protons, consistent with complex formation. However, each set of resonances was doubled in the ^1^H NMR spectrum (Figure S17, Supporting Information) and two carbene carbon atom resonances (δ_C_ 157.08 and 157.06 ppm) were observed in the ^13^C {^1^H} NMR spectrum at chemical shifts significantly upfield (Figure S18, Supporting Information) to the expected resonance of δ_C_ ∼176 ppm in C_6_D_6_ for the Au^I^‐CNC complex previously reported (**3 a**, Scheme 2).^[64]^ The identity of the reaction product was established unambiguously (X‐ray crystallography) as the zwitterionic Au^I^ bis(carbene) complex **1 b** from structural analysis of a suitable specimen of the yellow crystals grown from the concentrated reaction solution (CH_2_Cl_2_) layered with pentane.

The structures of **1 a**, **1 b**, **1 c**, and **2** in Scheme 1 were ultimately all elucidated by X‐ray crystallography and are briefly discussed in the Supporting Information (Figures S30 and S31). Despite being well‐characterized, the insolubility of **1 c** and the instability of **2** precluded their use in biological studies. All metalation attempts with modified ligand precursors **L1** and **L2** (where the phenyl substituents were replaced with the bulky aromatic Dipp group on N1, Schemes S4–S6, Supporting Information), were unsuccessful. To obtain the Au^III^ pincer complex to evaluate its potential as an anticancer agent and investigate its DNA affinity, sterically demanding Dipp groups were required on both the N1 and N3 wingtips of the trz rings to enforce pincer complex formation.

### Synthesis and characterization of N1,N3‐diarylated bis(1,2,3‐triazol‐5‐ylidene)carbazolide gold(I/III) complexes

**Figure 1:**
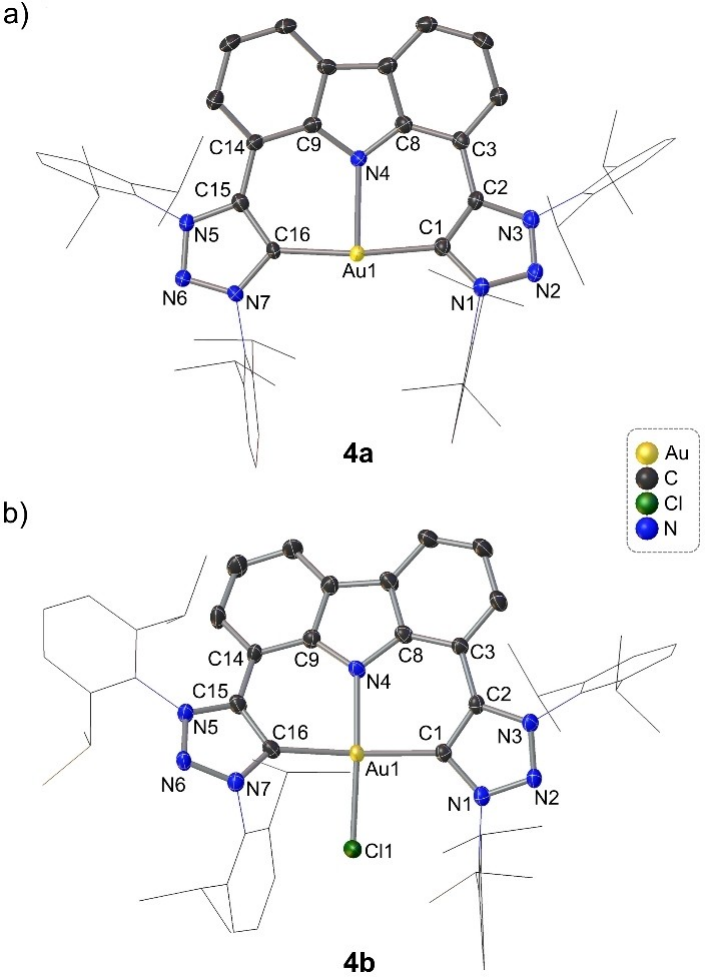
Molecular structures of a) **4 a** and b) **4 b** showing 50 % probability ellipsoids and partial atom‐numbering schemes. For clarity, the hydrogen atoms and counterions are omitted and the wingtip functionalities are displayed as wireframes. Selected bond lengths (Å) and angles (°) for **4 a**: Au1−C1 2.024(3), Au1−C16 2.021(3), Au1−N4 2.445(3), C1−Au1−C16 169.85(15), C1−Au1−N4 86.12(14), C16−Au1−N4 84.65(13). For **4 b**: Au1−C1 2.054(3), Au1−C16 2.040(4), Au1−N4 2.010(3), Au1−Cl1 2.266(10), C1−Au1−C16 171.54(14), N4−Au1−Cl1 172.97(9), C1−Au1−Cl1 92.83(10), C16−Au1−Cl1 90.24(10), C1−Au1−N4 89.86(12), C16−Au1−N4 88.01(12), C1−C2−C3−C8 4.6(6), C9−C14−C15−C16 −21.3(6).

The N1,N3‐diarylated triazolium salt **L3** was synthesized by the literature method^[63]^ and the synthesis of **L4** was adapted accordingly (Scheme S7). The synthesis of **L3** and **L4** follows a modified procedure for the cycloaddition of 1,3‐diaza‐2‐azoniaallene and alkynes to produce N1,N3‐diarylated‐1,2,3‐triazolium salts.^[73]^ Metalation of **L3** and **L4** with Au^I^ ensued via the concerted free‐carbene metalation route (Scheme 2).^[64]^ Formation of **4 a** was confirmed by the disappearance of the amine and triazolium CH protons in the ^1^H NMR spectrum (Figure S23, Supporting Information), in addition to the appearance of a carbene resonance at 175.0 ppm in the ^13^C NMR spectrum in CD_2_Cl_2_ (Figure S24, Supporting Information) and 175.9 ppm in C_6_D_6_. The carbene carbon signal is comparable to that reported for **3 a** (δ_C_ 176.0 ppm in C_6_D_6_) and the mesityl analogue (δ_C_ 175.8 ppm in C_6_D_6_).^[64]^ The carbene resonances of previously reported N1,N3‐diarylated ferrocenyl substituted Au^I^ complexes were within the range 160.9–183.1 ppm (CDCl_3_).^[60]^ Crystals of **4 a** were grown overnight from a concentrated CH_2_Cl_2_ solution layered with pentane at −20 °C. The molecular structure of **4 a** is shown in Figure 1a.

Like **3 a**, **4 a** features the unusual three‐coordinate T‐shape molecular geometry (Figure 1a). Other known three‐coordinate Au^I^ complexes mostly display distorted trigonal planar geometries.^[64]^ The C_carbene_ −Au−N_amido_ bond angle approaches 90° with an averaged bond angle of 85.4(14)° and the C_carbene_−Au−C_carbene_ bond deviates from linearity with a bond angle of 169.83(15)°. The average Au−C_carbene_ bond length (2.023(4) Å) is shorter than that for **3 a** (2.060 Å) but is within the range of Au−C_carbene_ bond lengths of known triazolylidene complexes (1.962–2.060 Å).^[54]^ The Au−N_amido_ bond (Au1−N4: 2.445(3) Å) is significantly longer than the Au−C_carbene_ bond and is comparable, albeit longer, to the Au−N_amido_ bond length reported for **3 a** (2.325(2) Å) and the mesityl‐analogue (2.412(3) Å). The N1‐Dipp substituents are orientated parallel to each other and rotated perpendicular to the carbazole plane, exposing the Au^I^ center.

After successful synthesis of the Au^I^ pincers **3 a** and **4 a**, facile oxidation to their corresponding Au^III^ complexes, **3 b** and **4 b**, was effected with iodobenzene dichloride as the oxidizing agent (step b, Scheme 2).^[74]^ Generally, oxidations of Au^I^‐NHC to Au^III^‐NHC complexes are accompanied by an upfield carbene ^13^C resonance shift of 20–30 ppm.^[75]^ However, in the case of **3 b** and **4 b**, the carbene resonances were not observed, in accord with the fluorinated analogue of **3 b**.^[65]^ Crystals of **4 b** were obtained from a saturated CH_2_Cl_2_ solution layered with toluene at room temperature and the solid‐state structure is shown in Figure 1b. The geometry of the Au^III^ ion is square planar with all the associated bond angles approaching 90° (avg C_carbene_−Au−N, 88.94(12)°; C_carbene_−Au−Cl, 91.54(10)°) and both bond angles of N−Au−Cl (172.97(9)°) and C_carbene_−Au−C_carbene_ (171.54(14)°) deviate slightly from linearity. The average Au−C_carbene_ bond length increased slightly to 2.047(4) Å compared with the Au^I^ precursor, **4 a** (2.023(3) Å), while the Au−N_amido_ bond length significantly decreased to 2.010(3) Å from 2.445(3) Å, consistent with the higher oxidation state for the metal.[^64^, ^65^] The C_carbene_−Au−C_carbene_ plane is slightly twisted in relation to the carbazole moiety with a twist angle of 15.30(3)°, whereby one of the triazoles rings (C1) is orientated 4.6° above the carbazole moiety mean plane, while the other triazole ring is canted by 21.3(6)° below the plane. The geometry of the solid‐state structure of **3 b** (Figure S32, Supporting Information) is similar to that of **4 b** (Figure 1b).

### Cytotoxicity

Preliminary cytotoxicity studies were conducted to identify active ligand precursors (**L3** and **L4**) and their complexes (**3 a**, **3 b**, **4 a** and **4 b**) against the breast cancer cell line MDA‐MB‐231. The cancer cells were exposed to two single point concentrations of 50 μM and 5 μM for 48 h and the percentage cell viability was determined using crystal violet assays (CVS, Figure S33, Supporting Information). At a concentration of 50 μM, all compounds inhibited the growth of the cells by more than 50 %, apart from the neutral Au^I^ pincer complex, **3 a**. At 5 μM only the cells treated with the cationic Au^III^ pincer complex **4 b** and the corresponding ligand precursor salt, **L4**, resulted in less than 50 % cell viability. At both test concentrations, **L4** and **4 b** were collectively the most cytotoxic towards MDA‐MB‐231 cancer cells.

The neutral Au^I^ complexes **3 a** and **4 a** exhibited low cytotoxicity and this can be attributed to their insolubility in dimethylsulfoxide (DMSO). Although the cationic Au^III^ complexes were soluble in DMSO (**4 b** to a greater extent than **3 b**), they precipitated in the culture medium overnight. The dicationic triazolium salts **L3** and **L4** were soluble in both DMSO and the culture medium. During the initial screening experiments, the DMSO concentration in the culture medium did not exceed 0.1 % (V/V). Due to the nucleophilic nature of DMSO, ligand dissociation of metal complexes in DMSO is not uncommon and can impact the results obtained from biological screening.^[76]^ We therefore investigated the stability of **4 b**, as a model compound, in DMSO over 48 h and a period of 2 months using ^1^H NMR spectroscopy (Figure S35, Supporting Information). Notably, even after 2 months in DMSO, no significant changes in the ^1^H NMR spectrum of the complex were evident, reflecting the lack of both chloride ion exchange and dissociation of the pincer ligand.

Since the triazolium salt **L4** and the corresponding Au^III^ complex **4 b** decreased cell viability below 50 % at both 50 and 5 μM during the preliminary experiment, these compounds were selected to undergo further screening to determine their half maximal inhibitory concentration (IC_50_) for MDA‐MB‐231 cells (Table 1). Toxicity towards healthy cells was established with the non‐tumorigenic endothelial cell line EA.hy926. The Au^III^ complex **4 b** was significantly cytotoxic against the chosen breast cancer cell line (MDA‐MB‐231) with a calculated IC_50_ value of 2.3 μM and a therapeutic index (T.I.) of 3.8 based on its IC_50_ value (8.6 μM, entry 1, Table 1) for the non‐tumorigenic cell line. Although direct comparison is not possible due to different incubation times, cytotoxicity assay methods, and cell lines, our limited data for **4 b** compare favorably with activities reported for other Au^III^ complexes, where the IC_50_ values range from 0.1–35 μM against various cell lines.[^26^, ^27^, ^28^, ^29^, ^30^, ^31^, ^32^, ^33^, ^34^, ^35^, ^36^, ^37^, ^38^, ^39^, ^40^] Importantly, the selectivity of **4 b** towards cancer cells also falls within the range reported for other Au^III^ complexes with T.I. values ranging from 2.5 to 147.[^30^, ^31^, ^32^, ^33^] Previously reported gold(I)‐NHC complexes with long aliphatic side‐chains exhibited IC_50_ values from 3.6–16.8 μM specifically against MDA‐MB‐231 cells after 96 hours of incubation.^[77]^ Gold(I)‐NHC complexes based on 4,5‐diarylimidazoles exhibited higher cytotoxicity towards MDA‐MB‐231 cells than the aliphatic gold(I) NHC complexes, with IC_50_ values of between 0.3 μM and 6.9 μM. The gold(III) analogues showed potent activity extending to the nanomolar range (0.5–4.4 μM; 72 h incubation).[^78^, ^79^] However, therapeutic indices were not reported for these compounds.

**Table 1:** Cytotoxicity data for **4b** and **L4** against a breast cancer cell line (MDA‐MB‐231) and non‐tumorigenic cell line (EA.hy926).

Entry	Compounds	IC_50_ (±SEM)^[a,b]^/μM		T.I.^[c]^
		MDA‐MB‐231	EA.hy926	
1	**4 b**	2.3 (±0.8)	8.6 (±2.1)	3.8
2	**L4**	0.4 (±0.1)	1.0 (±0.2)	2.7

[a] Expressed as average±standard error from mean (SEM) of three independent experiments, each with 6 replicates. [b] IC_50_ values were determined with the crystal violet staining (CVS) assay after 48 h of incubation with test compounds. [c] T.I. refers to the therapeutic index and is calculated from the ratio of the IC_50_ for normal cells (EA.hy926) relative to the IC_50_ for tumor cells (MDA‐MB‐231).

Interestingly, the triazolium salt **L4** was more potent than **4 b** towards both cell lines with IC_50_ values of 0.4 μM (MDA‐MB‐231) and 1.0 μM (EA.hy926). Previously reported pyridine‐based triazolium salts inhibited HeLa tumor cells with IC_50_ values of 54.4–91.6 μM,^[80]^ while fluorine‐substituted triazolium salts were significantly more cytotoxic (towards numerous tumor cell lines) with IC_50_ values as low as 1.7 μM.^[81]^ The salt **L4** is therefore more cytotoxic than previously reported triazolium salts, highlighting the fact that the ligand scaffold alone likely inhibits one or more cellular targets (currently unknown).

### Interaction of 4 b with GSH and DNA

#### Electronic spectroscopy

**Figure 2:**
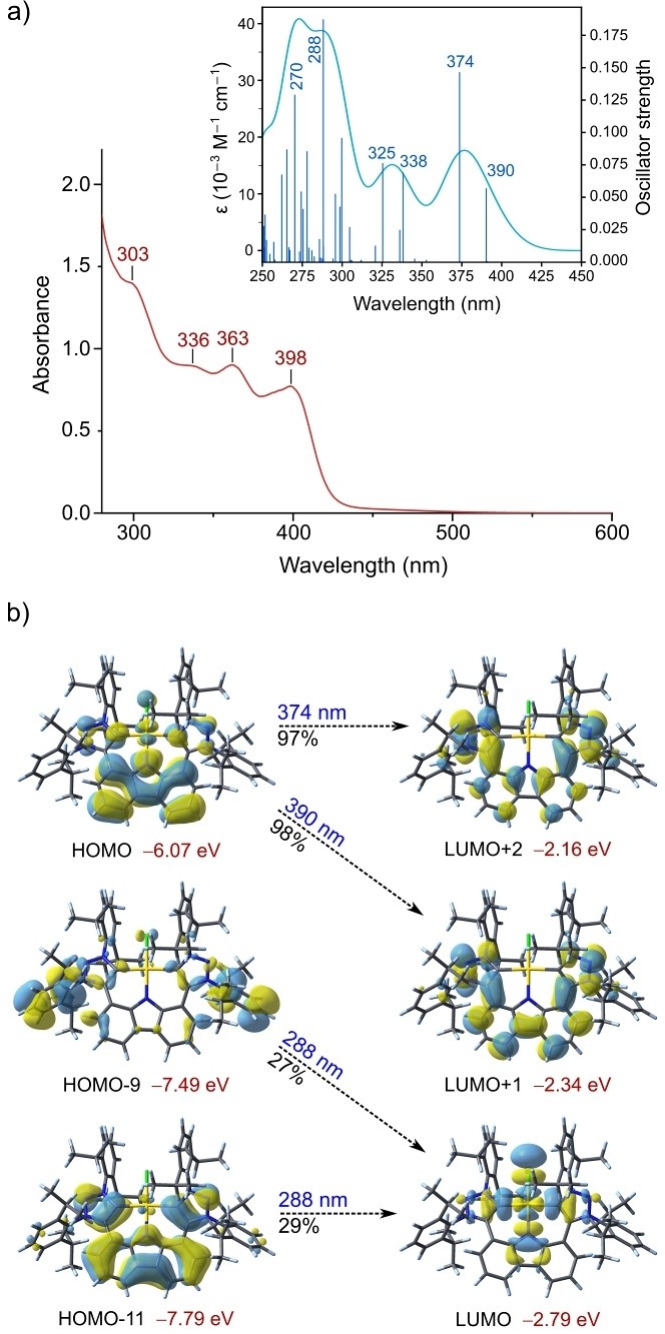
a) Experimental and DFT‐calculated (inset) electronic absorption spectra of *C*_2_‐symmetry **4 b** in DMSO. The absorption maxima (λ_max_) are indicated for the experimental spectrum at 303, 336, 363 and 398 nm (red line). The absorption envelope for the DFT‐calculated spectrum is plotted with a band width of 2200 cm^−1^ (full width at half maximum intensity, FWHM). b) Molecular orbitals involved in the three most intense absorption bands in the DFT‐calculated electronic spectrum of **4 b** in DMSO. The percentage contribution of the electronic transition to each band is indicated. The transition dipoles (not shown) will be polarized in the plane spanning the carbazole and triazole ring systems for the two lowest‐energy transitions.

The electronic structure of **4 b** in DMSO was characterized prior to investigating its ability to bind to DNA via UV‐vis spectroscopic titrations. The UV‐vis absorption spectrum of **4 b** in DMSO is shown in Figure 2a. Four absorption maxima are observed at 303 nm (ϵ=1.40× 10^4^ M^−1^ cm^−1^), 336 nm (ϵ=8.70× 10^3^ M^−1^ cm^−1^), 363 nm (ϵ=8.80× 10^3^ M^−1^ cm^−1^) and 398 nm (ϵ=7.49× 10^3^ M^−1^ cm^−1^). These values are consistent with those previously reported for the fluorinated analogue of **3 b**.^[65]^ In comparison, the absorbance maxima of **4 b** in 1x PBS (10 % DMSO) were similarly located at 298 nm (ϵ=1.40× 10^4^ M^−1^ cm^−1^), 362 nm (ϵ=9.17× 10^3^ M^−1^ cm^−1^) and 399 nm (ϵ=8.18× 10^3^ M^−1^ cm^−1^). An additional peak at 264 nm (ϵ=3.04× 10^4^ M^−1^ cm^−1^) was identified in 1x PBS due to the extended solvent window of water (190–1100 nm) as opposed to DMSO (268–1100 nm) and the absorbance maximum at 336 nm (present in DMSO) was not apparent in the electronic spectrum of **4 b** in 1x PBS (Figure 4, blue). The transitions (in DMSO only) were assigned using TD‐DFT calculations at the B3LYP−D3/Def‐2‐SVP level of theory; the DFT‐calculated electronic spectrum of **4 b** in DMSO (polarizable continuum model) is shown in the inset to Figure 2a. The predicted absorption maxima and the major contributions to each transition are given in Table S1, Supporting Information. Due to the limited solvent window of DMSO, the calculated absorbance peak at 270 nm is not experimentally observed in DMSO but is observed in 1x PBS (10 % DMSO) at 264 nm. The remaining experimental peaks relate closely to those predicted by the TD‐DFT calculations. For instance, the DFT‐calculated maxima at 390 nm and 374 nm agree with the experimental absorbance maxima of 398 nm and 363 nm, while the experimental peak at 336 nm is explained by two predicted band maxima at 325 nm and 338 nm (near‐equal intensity). The frontier molecular orbitals are involved in the lower energy transitions (336–398 nm). Specifically, the excitation of an electron from the HOMO to the LUMO+1 and the LUMO+2 contributes to the experimental transitions at 398 and 363 nm, respectively. The major contributor to the peak at 336 nm is from the excitation from HOMO−1 to LUMO+1 and LUMO+2. The HOMO and HOMO−1 are π‐bonding orbitals spanning the carbazole moiety, whereas the LUMO+1 and the LUMO+2 are antibonding in nature (π*) and predominantly localized on the triazolylidene rings (Figure 2b and Figure S36a, Supporting Information). Therefore, the lowest‐energy transitions of **4 b** may be assigned to intraligand charge transfer (ILCT) with dominant π→π* character. Because the LUMO has significant 5dx^2^–y^2^ character, transitions such as HOMO–11→LUMO are effectively in‐plane LMCT transitions (ligand‐to‐metal charge transfer).

#### Reactions with glutathione

The stability of **4 b** in the presence of the intracellular reductant glutathione (GSH) was evaluated. A solution of GSH (100 mM, D_2_O) was added to an NMR tube containing **4 b** (10 mg, DMSO‐*d*_6_) to obtain a final molar ratio of 1.5 : 1 (GSH:**4 b**). ^1^H NMR spectra were recorded at intervals over a 24‐hour period at room temperature (Figure S38, Supporting Information). A new set of peaks appeared in the ^1^H NMR spectrum of **4 b** in the presence of excess GSH, indicative of the formation of a **4 b**‐GS(H) adduct instead of reduction to the Au^I^ complex (no color change or red precipitate was observed) or demetallation (as no triazolium protons appeared in the ^1^H NMR spectrum either). Adduct formation was confirmed by MS with the appearance of the dicationic molecular ion peak at m/z=722.3282 (Figure S39, Supporting Information), corresponding to either [**4 b** – Cl+GSH]^2+^ or [**4 b‐SG** – Cl+H]^2+^. Exact structure elucidation of the formed adduct was not possible, i. e., it is not known if the thiol or a terminal amine of GSH displaces the chlorido ligand. Importantly, it is shown that GSH does not reduce **4 b** to **4 a**. From the ^1^H NMR spectrum of **4 a** (Figure S23, Supporting Information), the Au^I^ redox state for the complex is characterized by the triplet signal for carbazole proton H1c appearing upfield (6.65 ppm) of the doublet for carbazole proton H1b (6.95 ppm). In the spectra shown in Figure S38 for the reaction of **4 b** with GSH, the reverse pattern exists with the doublet for carbazole proton H1b occurring upfield (6.8–6.9 ppm) relative to the triplet for proton H1c (∼7.2 ppm). In short, the ^1^H NMR spectra show no evidence for anything but the Au^III^ state for the complex throughout the reaction of **4 b** with GSH.

**Figure 3:**
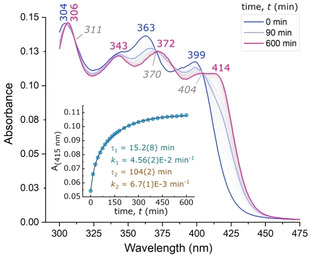
Electronic absorption spectra of 15 μM **4 b** in a water (10 % V/V) and DMSO mixture containing GSH at a molar ratio of 1 : 7 [**4 b**]:[GSH]. The inset shows the change in absorbance at 415 nm with time. The data are fitted to a standard double exponential kinetic function, ***A***=***B***_1_ *e* ^(−x/t1)^+***B***_2_ *e* ^(−x/t2)^+***A***_∞_, where ***B***_**1**_ and ***B***_**2**_ are adjustable pre‐exponential factors, *t*_1_ and *t*_2_ are the time constants, and **A_∞_** the limiting absorbance. The derived parameters are *k*_n_=1/*t*_n_ and τ_n_=*t*_n_ ln(2); *R* ^2^ for the fit is 0.9998.

The reaction of **4 b** and GSH was also monitored over time by electronic spectroscopy in the UV‐visible region using a 1 : 7 mole ratio (**4 b**:GSH) of the two reactants and is shown in Figure 3. The change in the spectrum from **4 b** to **4 b**‐GS(H) is associated with the appearance of four isosbestic points at 305, 311, 370, and 404 nm. The lowest‐energy band at 399 nm red‐shifts by 15 nm upon formation of the adduct, while the band at 363 nm red‐shifts by 9 nm to 372 nm. Inspection of the region near 385 nm indicates that two separate isosbestic points are established close to one another over the time course of the reaction, suggesting a biphasic reaction overall. The kinetic trace recorded from the time‐dependent spectra at 415 nm confirms the biphasic nature of the reaction with two distinct rate constants: *k*_1_=4.56(2)×10^−2^ min^−1^ and *k*_2_=6.7(1)×10^−3^ min^−1^. The mean lifetimes for these steps are τ_1_=15.2(8) min and τ_2_=104(2) min.

A full kinetic study of the mechanism of the reaction should be feasible and provide valuable information going forward, but clearly falls beyond the scope of the current article. Of particular importance, however, is the fact that GSH does not reductively demetallate **4 b**; instead a spectroscopically distinct Au^III^‐GSH adduct is formed and maintained in the presence of excess GSH. Considering that many Au^III^ complexes are reduced in vivo by GSH to either Au^I^ species, or in fact completely to Au^0^,^[19]^ the present study highlights the remarkable stability of Au^III^ engendered by the present class CNC pincer ligands.

#### Spectroscopic titrations

**Figure 4:**
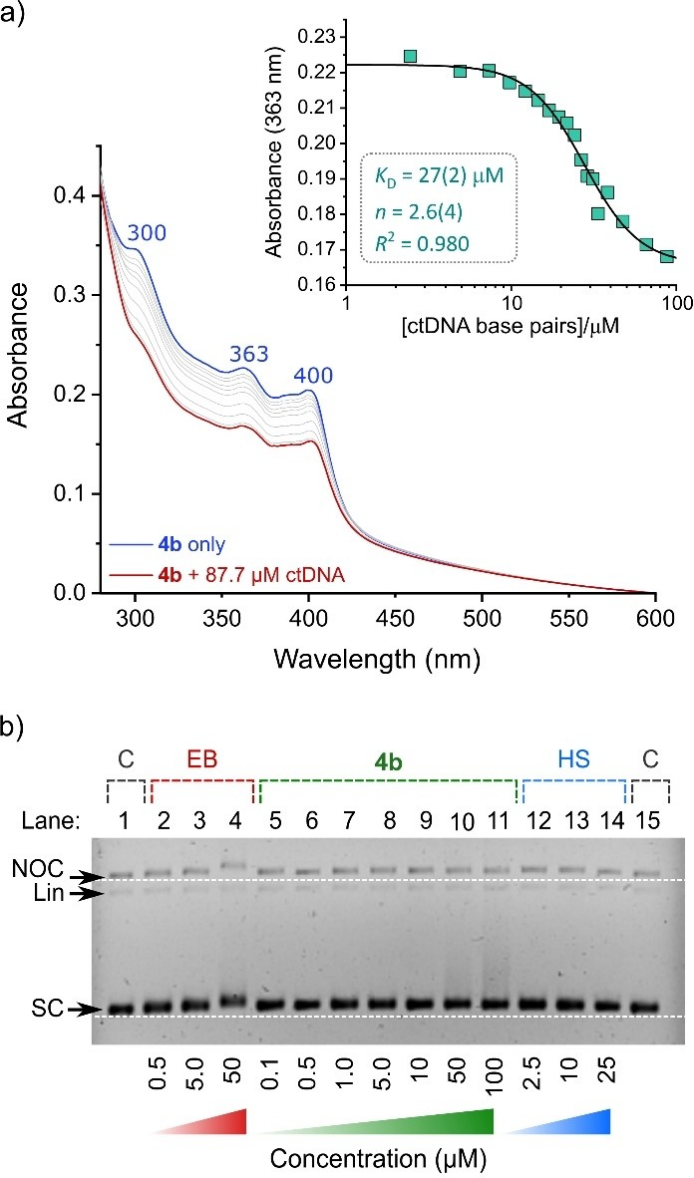
a) UV‐vis absorption spectra of **4 b** in 1 x PBS (10 % V/V DMSO) before (30.8 μM, blue spectrum) and after sequential additions of ctDNA (final [ctDNA]=87.7 μM, red spectrum). The spectra have been corrected for dilution and only selected intermediate spectra are plotted; the sloping (yet unchanging) background absorption reflects some aggregation of the chromophore in the buffer. Inset: change in the absorbance at 363 nm as a function of [ctDNA] fitted to the Hill binding isotherm. b) EMSA agarose gel for **4 b** with pUC57 plasmid DNA (12.5 ng/well, 1x TAE buffer, 5 % V/V DMSO). The DNA forms are supercoiled (SC), nicked‐open circular (NOC), and linear (Lin). Control lanes (1 and 15, C) contain pUC57 DNA only. Lanes with increasing concentrations of ethidium bromide (EB, 2–4), **4 b** (5–11), and Hoechst 33258 (HS, 12–14) compare the behaviour of a DNA intercalator, the Au^III^ complex, and a DNA minor groove binder, respectively. Experiments were done in triplicate and a representative assay is shown.

The interaction between **4 b** and DNA was investigated by spectroscopic titrations with ctDNA (calf thymus DNA) as the substrate. Figure 4 shows the change in the electronic spectrum of **4 b** for a representative experiment of three replicates, whereby the highest mole ratio of [ctDNA]:[**4 b**] obtained, before turbidity due to aggregation or partial precipitation became pronounced, was 3 : 1. In Figure 4a, the blue‐colored spectrum is that of **4 b** prior to the addition of ctDNA. The red spectrum represents the electronic spectrum of **4 b** in the presence of 87.7 μM ctDNA, after incremental additions of ctDNA (represented in grey). A non‐linear decrease in absorbance intensity occurs throughout the spectral range with increasing concentration of ctDNA. The band maxima at 300, 363 and 400 nm all decreased in intensity by ∼25 %. Minor bathochromic shifts in the peak energies are evident (∼2 nm) and no isosbestic points are visible. The change in the absorbance of **4 b** at 363 nm as a function of the concentration of ctDNA is shown in the inset of Figure 4a; significant hypochromicity is evident (absorbance decrease ∼0.06). The hypochromic response was fitted to the Hill model^[82]^ (*R* ^2^=0.9763), yielding an associative binding constant (*K*_A_) of 3.7(3)×10^4^ M^−1^. The fact that the absorption spectrum of **4 b** does not change significantly (e. g., a large bathochromic shift with discernible isosbestic points) indicates that additional experiments are needed to confirm the binding event and delineate a possible mode of interaction of **4 b** with ctDNA (*vide infra*). The spectroscopic changes shown in Figure 4a are consistent with those reported for cationic Ru^II^ polypyridine complexes^[83]^ and an anionic DNA‐binding probe for which a systematic drop in UV‐vis absorption intensity (300–425 nm) with increasing ctDNA concentration was observed (also without the establishment of isosbestic points).^[84]^

The spectroscopic behavior of **4 b** is similar to that of psoralen with ctDNA.^[85]^ In an extensive study, which hinged on several complementary methods, Zhou et al. showed how the uncharged planar aromatic natural product binds to ctDNA by intercalation with a *K*_A_ of 5.67×10^3^ M^−1^ (37 °C, pH 7.4 Tris‐HCl buffer). The binding constant of **4 b** with ctDNA is nearly an order of magnitude larger than that of psoralen. The modest *K*_A_ value measured here for **4 b** suggests that the Au^III^ pincer probably interacts with ctDNA via partial or complete intercalation similar to the Ru^II^ complex [Ru(phen)_3_]^2+^,^[86]^ where phen=1,10‐phenanthroline. Indeed, some of the best mononuclear polycationic DNA intercalators with ligands such as dppz (dppz=dipyrido[3,2‐*a*:20′,30′‐*c*]phenazine), which deeply penetrate the DNA base stack, e. g., [Ru(phen)_2_(dppz)]^2+^,^[87]^ have *K*_A_∼3×10^6^ M^−1^,^[88]^ which is about two orders of magnitude larger than that measured for **4 b**, supporting the idea that partial intercalative binding is likely.

#### Electrophoresis

Agarose gel electrophoretic mobility shift assays (EMSAs)^[89]^ were used to assess changes to the secondary and tertiary structure of pUC57 plasmid DNA^[90]^ as a function of the concentration of **4 b**, ethidium bromide (EB), and Hoechst 33258 (HS) after equilibration with the DNA target. From Figure 4b, the DNA intercalator control^[91]^ (EB) clearly reduces the migration of supercoiled (SC), linear, and nicked open circular (NOC) DNA forms with the effect being most pronounced at 50 μM for SC and NOC DNA (lane 4). This is the expected dose‐response profile for EB since intercalation partly unwinds and lengthens the dsDNA helix by ∼3.4 Å for each bound EB molecule,[^92^, ^93^, ^94^] increasing hydrodynamic drag, and consequently retarding the bulk mobility of the macromolecule within each band. A similar, though less marked profile is seen for **4 b** (lanes 5–11) and notable drag of the SC form is seen only when [**4 b**]≥50 μM. This suggests the Au^III^ complex is a *partial* DNA intercalator, commensurate with its generally rather significant steric bulk (compared with EB) and the fact that the carbazole ring system is only partially exposed for π‐stacking interactions with DNA bases (Figure S37, Supporting Information). The behavior of **4 b** is *inconsistent* with that of Hoechst 33258 (HS, lanes 12–14), which shows dose‐dependent compaction of the DNA bands and thus slightly enhanced mobility of all DNA forms at the highest concentration (25 μM, lane 14). HS is a classical near‐planar (yet twistable), unencumbered crescent‐shaped bis(benzimidazole) dye that targets the DNA minor groove with a high binding efficacy and a preference for 5′‐AATT‐3′ sites.[^95^, ^96^] Given the shape and steric bulk of **4 b**, interaction with DNA via a classical groove binding mechanism seems unlikely, as supported by the EMSA data.

#### Viscometry and DNA thermal melt analysis

**Figure 5:**
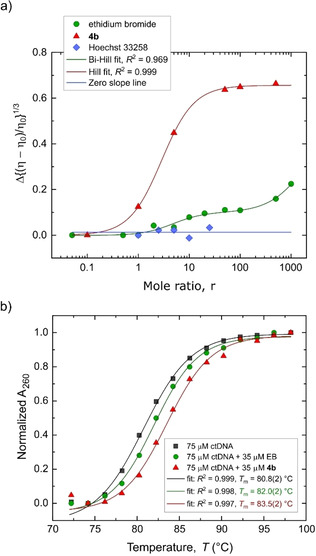
a) Plot of the change in relative viscosity of solutions of calf thymus DNA (ctDNA) as a function of *r*, where *r* is the analyte/DNA mole ratio (based on the DNA base pair concentration). The measurements were conducted at 37 °C in 1x phosphate‐buffered saline (PBS) containing 10 % (V/V) DMSO. Hoechst 33258 (HS) and ethidium bromide (EB) are the groove‐binder and intercalator controls, respectively. The data comprise several independent experiments and were fitted to single‐ and double‐Hill sigmoidal functions. For **4 b**, *r*_50_=2.9(1) while for EB, *r*_50(1)_=4.7(1.5) and *r*_50(2)_=1.9(5)×10^3^, where *r*_50_ is the mole ratio at which 50 % dissociation occurs. b) ctDNA melt curves recorded at equivalent concentrations of added EB and **4 b**. The Δ*T*_m_ values for EB and the gold complex are +1.8(2) and +2.7(2) °C, respectively, under non‐saturating conditions. The positive Δ*T*_m_ values reflect intercalation and stabilization of the DNA duplex; however, neither represent limiting Δ*T*_m_^max^ values. The data are fitted to Boltzmann sigmoidal functions.

Viscosity measurements employing a sheared ctDNA substrate were used to establish the effect of increasing the dose of **4 b** on the tertiary structure of the DNA target. Figure 5a shows the change in the relative viscosity (η) of ctDNA (compared to the viscosity of ctDNA in 1x PBS and 10 % DMSO, η_0_) against increasing mole ratios (*r*) of **4 b**, EB and HS to a fixed concentration (in base pairs) of ctDNA. The most striking observation is the significant increase (∼60 %) in the relative viscosity of ctDNA induced by **4 b** compared with the more modest increase (∼20 %) from intercalated EB. **4 b** clearly interacts with ctDNA more profoundly than EB. The viscosity increase induced by **4 b** is sigmoidal and follows a single‐step Hill binding isotherm, saturating when *r*>100. EB, in contrast, has a two‐step isotherm over the full range of *r* investigated, but is similarly saturated for the first step at *r* ∼ 100. The origin of the second step in the relative viscosity curve for EB is unknown but could reflect the onset of precipitation since we have observed that the addition of excess EB to a ctDNA solution in a microcentrifuge tube both gels and partly precipitates the DNA.

The viscosity of DNA is sensitive to changes in the axial length of the double helix. Intercalator compounds insert between the base pairs, the latter being separated by ∼7 Å to accommodate the guest. The double helix unwinds, lengthens, and stiffens, culminating in a commensurate increase in the solution viscosity. Groove‐binders (e. g., HS and DAPI) minimally perturb the structure of the DNA helix; a negligible change in the hydrodynamic behavior of the macromolecule and thus viscosity of the DNA solution is observed.[^97^, ^98^] Complexes such as [Ru(phen)_2_dppz]^2+^ increase the viscosity of DNA linearly with increasing dose.^[83]^ A similar linear increase was observed for [Au^III^(CNC)(L)]^+^ intercalators, where L=1‐dimethylimidazolium or *N*,*N*′‐dimethylimidazol‐2‐ylidene.[^28^, ^29^] Interestingly, the viscosity of DNA decreases in the presence of some partial intercalators such as Δ‐[Ru(phen)_3_]^2+^ and the organic insecticide acetamiprid (ACT). These compounds can act as a wedge by splaying the bases at the intercalation site on one strand, leading to a partial inward collapse of the base pair on the complementary strand.[^99^, ^100^, ^101^] The result is a bend in the DNA and a shortening of the effective length of the helix. The same effect is induced by the covalent binder cisplatin.[^6^, ^10^] The significant non‐linear increase in the viscosity of ctDNA induced by **4 b** is unique, reflecting neither the behavior of a classical intercalator nor a conventional partial intercalator. Further, the collective experimental evidence for **4 b** is *inconsistent* with covalent binding to bases (e. g., guanine) or groove‐binding of the complex.

We studied the change in the melting temperature (*T*_m_) of ctDNA in the presence of **4 b** to confirm intercalation (full or partial) as the likely DNA binding mechanism for the complex (Figure 5b). At the sub‐saturating [**4 b**]/[DNA] ratio of 1/3 used in our experiment, **4 b** and EB induced Δ*T*_m_ values of +2.7(2) °C and +1.8(2) °C, respectively (identical conditions). The interaction between **4 b** and ctDNA is clearly more stabilizing than that between EB and ctDNA, despite the gold complex having a similar, if not slightly lower, ctDNA affinity constant to EB (*K*_A_=4.94×10^5^ M^−1^).^[100]^ The thermal melt data are consistent with the viscosity data recorded for the gold complex since **4 b** increased the viscosity of ctDNA solutions more than EB. However, because sub‐saturating concentrations of both **4 b** and EB were used to avoid precipitation, the value of Δ*T*_m_^max^ remains unknown for this system. Normally, Δ*T*_m_ is dependent on the complex/DNA mole ratio as this reflects the position of the binding equilibrium. Δ*T*_m_ is also strongly influenced by the ionic strength of the buffer, with solutions of high ionic strength giving small positive Δ*T*_m_ values for intercalators but very large positive Δ*T*_m_ values (>+20 °C) for the same compounds at low ionic strength.^[102]^ Exemplifying the former variable, Δ*T*_m_ induced by [Ru(phen)_2_(pdppz)]^2+^ increased with increasing *r* (i. e., [complex]/[DNA]), with Δ*T*_m_<+2.0 °C at *r*=1/20 and Δ*T*_m_>+6 °C at *r*=1/10.^[83]^ Of relevance here, a Δ*T*_m_ value of +16.5 °C was reported for ctDNA with the complex [Au^III^(CNC)(L)]^+^, where L=*N*,*N*′‐dimethylimidazolium, at a 1 : 1 molar ratio.^[28]^ The degree to which partial intercalators change *T*_m_ also varies. For instance, [Ru(phen)_3_]^2+^ induces a Δ*T*_m_^max^ of +20 °C at *r*=1/6 in a low ionic strength buffer,^[102]^ whereas the partial intercalator ACT only increased the *T*_m_ of ctDNA by 3 °C at *r*=1/3 (pH 7.4, ionic strength not specified).^[99]^ The latter value is comparable to Δ*T*_m_ obtained here for **4 b**.

The ctDNA affinity constant (*K*_A_) together with the EMSA and thermal melt data suggests that **4 b** interacts with dsDNA via partial intercalation. The viscosity data, however, are unique. Conventional partial intercalators such as Δ‐[Ru(phen)_3_]^2+^ decrease the relative viscosity of ctDNA by bending or kinking the helical axis.^[100]^ The evidence for **4 b** thus points to an unconventional partial intercalative binding mechanism.

#### Circular and linear dichroism spectroscopy

**Figure 6:**
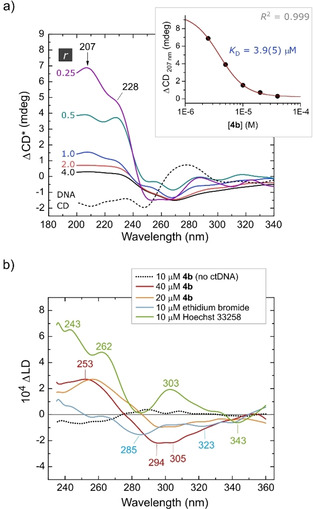
Circular and linear dichroism spectra delineating the interaction of **4 b** with ctDNA. All spectra are smoothed (8‐point fast Fourier transform) and were recorded in a Couette flow cell which orients the DNA colinear with the shear flow axis (i. e., flow direction). a) Difference CD spectra as a function of the mole ratio *r* of the gold complex to ctDNA base pairs (10 μM throughout) in PBS‐DMSO (10 % V/V) buffer at 37 °C. The difference spectra are corrected for dilution (normalized to an effective *r*=1) and have the native ctDNA CD spectrum subtracted throughout. The inset shows the best fit of the data at 207 nm to the Hill function (*K*_A_=2.6(3)×10^5^ M^−1^, Hill coefficient *n*=1.9(3)). b) Difference LD spectra (37 °C) for **4 b** bound to ctDNA. Spectra for Hoechst 33258 (minor groove binder) and ethidium bromide (intercalator) are included. The samples, DNA concentration, and buffer were the same as in Part a).

CD and LD spectra were recorded for solutions of sheared ctDNA with increasing doses of **4 b** (Figure 6). Because **4 b** is achiral, neither CD nor LD spectroscopy gives an appropriate absorption spectrum for the compound in the absence of DNA. Observation of an induced CD (ICD) spectrum in the presence of dsDNA signals stable complex formation, with minor groove binders usually giving positive ICD signals and intercalators negative ICD signals for the ligand's main visible or near‐UV π→π* bands.[^103^, ^104^]

From Figure 6a, increasing the dose of **4 b** from 0.25 to 4.0 mole equivalents of compound per DNA base pair attenuates the positive intensity bands in the far‐UV difference ICD spectrum. The strong positive ΔCD signal observed at the lowest value of *r* (0.25) suggests that **4 b** is bound to ctDNA and experiences a significant positive ICD spectrum; the π→π* transition dipoles concerned are evidently oriented orthogonal to the helix axis, which is indicative of intercalation.^[104]^ As the equilibrium is driven to completion by the addition of excess **4 b**, the ICD spectrum gains negative intensity over the 240–450 nm spectral range (Figure S42 and S43, Supporting Information), in accord with the behavior of EB[^105^, ^106^, ^107^, ^108^] and luminescent Ru^II^ polypyridyl complexes with extended dppz‐type intercalator ligands.^[83]^ Our spectra are consistent with those reported by Marrington et al.[^108^, ^109^] for EB bound to ctDNA recorded in a Couette flow cell of similar design to that used here. Because the CD spectrum for native ctDNA shows a mainly positive absorption band in this region, the response in the ΔCD spectrum is progressively damped by the addition of **4 b**, giving a moderately negative ΔCD band profile.

The sigmoidal change in the ΔCD spectrum at 207 nm with increasing [**4 b**] affords an exemplary fit to the classic Hill binding isotherm with a ligand dissociation constant, *K*_D_, of 3.9(5) μM at 37 °C (*K*_A_=2.6(4)×10^5^ M^−1^). Performing the experiment as a two‐fold dilution series with [ctDNA] fixed averted precipitation of the adduct. The dissociation constant measured by CD spectroscopy is therefore likely to be an accurate reflection of the true affinity of **4 b** for sheared ctDNA. Collectively, Figures 4a and 6a indicate that the binding constant for **4 b** with ctDNA falls in the range 3.7(3)×10^4^ M^−1^ to 2.6(4)×10^5^ M^−1^.

Couette flow linear dichroism (LD) spectroscopy was used to further delineate how **4 b** interacts with ctDNA oriented in a fluid shear flow field. The principles underlying the method are given in Figure S44. Figure 6b strikingly confirms the view that **4 b** binds to ctDNA via intercalation. In the absence of ctDNA, the achiral compound lacks orientation in the shear flow field and exhibits no linear dichroism (as expected). At an Au:DNA base pair ratio of 4.0 (40 μM **4 b**), the LD spectrum (plotted as a difference spectrum) is characterized by two negative bands peaking at 305 and 294 nm. The spectrum switches sign at 274 nm and exhibits a positive band maximum at 253 nm. From the DFT‐calculated electronic spectrum of **4 b** (Figure 2), this region of the spectrum is dominated by intense π→π* and CT bands. The LD spectrum itself exhibits negative absorption intensity throughout the range from 230–360 nm (Figure S45, Supporting Information); the positive band in the difference spectrum <274 nm reflects subtraction of the native ctDNA LD spectrum, which is more negative than that of the ctDNA⋅{**4 b**} complex below 274 nm. The negative LD and ΔLD bands for ctDNA⋅{**4 b**} confirm that the compound is bound to ctDNA as an intercalator with the π→π* transition dipole moments of the carbazole ring moiety lying perpendicular to the shear flow (and thus the DNA helix) axis. The spectrum of HS bound to ctDNA within the minor groove (Figure 6b) gives the expected response with all major absorption bands displaying a positive ΔLD signal consistent with orientation of the in‐plane π→π* transition dipoles along the axis of the minor groove, which is canted (∼45°) relative to the fluid flow and DNA helix axes.^[110]^ The LD spectra for **4 b** are clearly distinct from those of Hoechst 33258 and inconsistent with the behavior of a minor groove binder.

#### Biomolecular simulations

The present compounds were initially designed around the hypothesis that ligands such as **L2** (Scheme 1) might be sufficiently sterically unencumbered when coordinated to Au^III^ to provide stable cationic species, [Au^III^Cl(**L2**)]^+^, suitable for targeting normal dsDNA (see *Glide*[^111^, ^112^, ^113^, ^114^, ^115^] simulations, Figures S46–S48 and Table S2, Supporting Information) as metallointercalators and/or metalloinserters.[^116^, ^117^, ^118^]

From Figures 4–6, the experimental data suggest that **4 b** interacts with DNA via unconventional partial intercalation. Given the steric bulk of the pincer ligand, we were not surprised to find that *Glide* produced relatively few poses with **4 b** bound to standard DNA intercalation sites (Table S2, Supporting Information). However, an example depicting the way **4 b** might partially intercalate DNA was obtained with the palindromic sequence used by Barton et al. to study intercalation and insertion of 5′‐D(CGGAAATTACCG)‐3′ by the cationic ruthenium complex [Ru(bpy)_2_(dppz)]^2+^ (PDB code: 4E1U).^[118]^ Like the Ru^II^ complex, **4 b** was able to target the central 5′‐AT‐3′ step in the oligonucleotide (Figure S48, Supporting Information), albeit with a relatively modest *Glide* docking score (Δ*G*_bind_=−7.93 kcal/mol). The binding mode is best described as a part‐intercalation/part‐minor‐groove interaction. Interestingly, **4 b** readily targeted insertion sites located at base pair mismatches (e. g., 5′‐CGGAAA‐3′ paired with 3′‐GCCATT‐5′ as the partner strand). The *Glide* docking scores for these dsDNA substrates averaged −8.2 ± 1.0 kcal/mol, with the best adenine base mismatch insertion achieving a docking score of −9.55 kcal/mol (5′‐CGGAAATTACCG‐3′ target; PDB code: 3GSJ).^[117]^

Although the *Glide* docking algorithm deploys a fixed grid for the macromolecular target (ligand conformations were flexibly sampled), which could account for the lack of suitable binding poses for **4 b** with many standard intercalation sites, the fact remains that, experimentally, **4 b** binds to sheared ctDNA (Figure 5a) and plasmid DNA (Figure 4b). To better understand what type of DNA targets might be suitable for **4 b**, we considered some less common DNA substrates, including hairpin loops, *Z*‐form DNA (Table S3, Figure S49), and DNA junctions. Some of these species could, in principle, be present in sheared ctDNA. Moreover, structures such as DNA three‐ and four‐way junctions are crucial to several genomic processes, including DNA recombination and replication, in vivo.[^119^, ^120^, ^121^] In our view, stabilization of three‐ and four‐way DNA junctions (or similar clusters formed from single‐strand overhangs that might be present in sheared dsDNA) could explain the unusually large viscosity increase observed with increasing [**4 b**] (Figure 5a), since this is akin to cross‐linking and entangling polymer strands. Inspection of the EMSA data for **4 b** with pUC57 plasmid DNA (Figure S40, lanes 5–7) reveals a dose‐dependent increase in the fraction of dimeric nicked‐open circular DNA forms present in the solution. This behavior is unique to **4 b** as it is not mirrored in the lanes for EB or HS, providing experimental support for the idea that **4 b** facilitates intermolecular DNA−DNA interactions.

**Figure 7:**
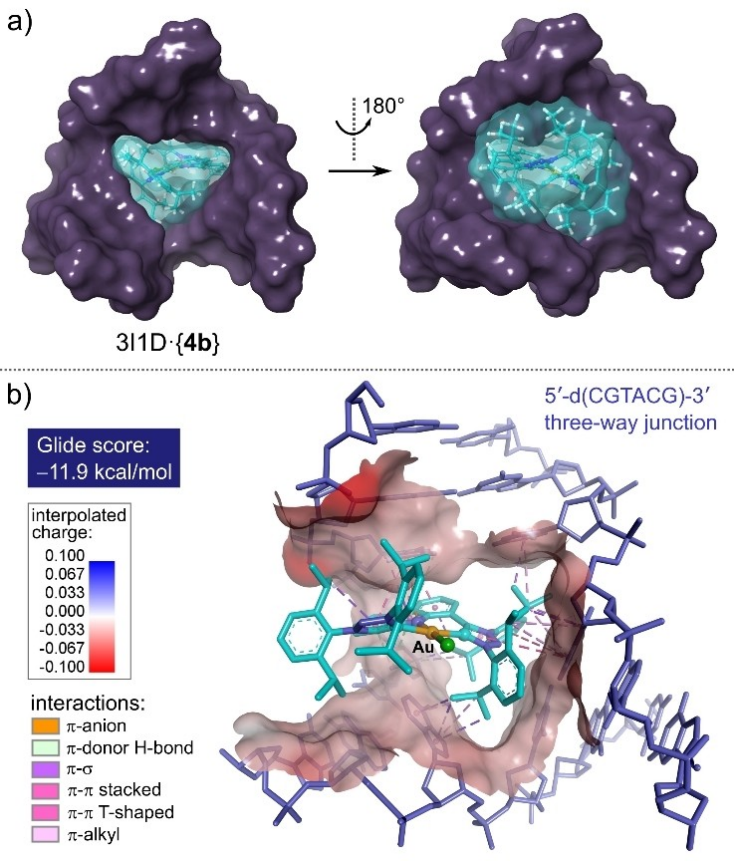
a) Lowest‐energy pose for **4 b** docked within the pocket of the DNA three‐way junction taken from PDB code 3I1D. The carbazole ring system of the metal complex is intercalated at the 5′‐TA‐3′ step within each strand. The shape complementarity between the metal complex (light blue) and the DNA target (purple) is discernible from the two views down the 3‐fold axis. Molecular surfaces were calculated using a 1.4 Å probe radius. b) View of the noncovalent interactions between the metal complex and the DNA bases (A and T) lining the triangular cavity of the three‐way junction (H atoms omitted for clarity). C−H⋅⋅⋅π, π⋅⋅⋅σ, and π⋅⋅⋅π interactions between the carbazole and Dipp groups of the complex and the DNA bases are dominant. A π⋅⋅⋅anion interaction is also present between the carbazole nitrogen and closest stacked thymine residue.

We investigated whether **4 b** could target a three‐way junction (3WJ) formed by the palindromic hexameric oligonucleotide 5′‐d(CGTACG)‐3′ (Figure 7), as exemplified in recent work by Duskova et al.,^[122]^ where unconventional large organic macrocycles, cations, and metal‐organic compounds, were screened for their ability to target DNA 3WJs in vitro. The binding site in this junction comprises the three 5′‐TA‐3′ steps, which are slightly splayed, at the center of the structure. As shown in Figure 7, **4 b** has a remarkably good shape complementarity for the binding pocket and forms a stable noncovalent adduct with a highly exergonic binding free energy (docking score ranging from −10.7 to −11.9 kcal/mol, depending on the grid parameters used). The dominant noncovalent interactions between **4 b** and the DNA target span the carbazole ring system and the two Dipp substituents appended to one triazole ring; they are hydrophobic in nature (e. g., C−H⋅⋅⋅π, π⋅⋅⋅π, and π⋅⋅⋅alkyl interactions) and do not include classical hydrogen bonds. The molecular surface of the triangular pocket is negatively charged and cation **4 b** exhibits the required charge complementarity to interact with the DNA target electrostatically. Importantly, the carbazole ring clearly intercalates one of the three 5′‐TA‐3′ steps in the 3WJ (principally by π‐stacking on the thymine base of the pair). This interaction amounts to intercalation of a single‐stranded section of DNA within the central triangular hole of the 3WJ, and suggests that our analysis of the EMSA, thermal melt, and viscosity data for **4 b** (admittedly with heterogeneous DNA targets) holds some merit.

The above in silico result clearly requires experimental verification. Although synthetic dsDNA oligomers spontaneously form 3WJs after strand separation and annealing,[^122^, ^123^, ^124^] we are unaware of reports describing spontaneous formation of 3WJs in samples of sheared or plasmid DNA in vitro, despite their formation being thermodynamically favored and the fact that they are vital, dynamic structural entities found at the DNA replication fork in all living cells.[^125^, ^126^, ^127^] It is difficult to model these species without X‐ray data in *Glide*, but local unwinding to expose 5′‐TA‐3′ steps, base extrusions, or transient openings^[127]^ in dynamic dsDNA conformations could broaden the range of DNA substrates targeted by these compounds. Interestingly, larger DNA four‐way junctions also form spontaneously in solution.^[128]^ One implication is that fragments of sheared dsDNA might contain oligomers with the correct sequence (and self‐complementary sticky ends) to self‐assemble into 3WJs or higher‐order structures.^[124]^ If these species exist in the solutions used for our rolling‐ball viscometry experiments, which employ a gold‐coated steel ball, **4 b** might act as a template for their self‐assembly (perhaps in unison with physisorption of the DNA fragments on the gold surface),^[129]^ thereby dramatically increasing the viscosity of the ctDNA target beyond that seen for EB.

Docking experiments are essentially speculative. However, in silico interaction of **4 b** with a well‐characterized DNA 3WJ is notable because it confirms (i) that **4 b** can, despite its steric bulk, theoretically bind to at least one DNA target with favorable in silico thermodynamics and (ii) suggests a possible mechanism whereby the compound, which is resistant to reduction by glutathione, may become cytotoxic to cells. Specifically, DNA junctions occur at DNA replication and transcription forks.[^130^, ^131^] If **4 b** can enter tumor cell nuclei and target genomic DNA, then it is conceivable that disruption of replication/transcription could culminate in caspase‐mediated cell death. Future studies will either support or refute this mechanism.

## Conclusions

Drug design is dependent on the synthetic feasibility of the intended drug, as eloquently illustrated in this study. Deploying a concerted free carbene route to synthesize mononuclear tridentate pincer complexes of Au^I^ (**4 a** and the known **3 a**), with subsequent oxidation to Au^III^ (**4 b** and **3 b**), was successful only if the diarylated triazolium precursors **L3** and **L4** were employed. Of the diarylated compounds, the precursor ligand salt **L4** and corresponding Au^III^ complex, **4 b**, were notably cytotoxic against the breast cancer cell line MDA‐MB‐231.

The interaction between **4 b** and DNA was investigated with multiple techniques, which suggested that **4 b** is a partial DNA intercalator with an unconventional interaction relative to known partial‐ or full DNA intercalators. The bulky Dipp groups appear to hinder classical modes of DNA intercalation for **4 b**. Biomolecular simulations revealed that **4 b** can selectively target DNA three‐way junctions (3WJs) over dsDNA in silico, with the DNA affinity of the compound for multiple binding sites being only marginally lower than that of ethidium bromide on average. *Z*‐form DNA was also targeted. Notably, **4 b** does not reduce to Au^I^ or demetallate in the presence of excess glutathione (GSH). The cytotoxicity, DNA‐targeting action, and redox stability of **4 b** establish a positive foundation on which to develop this class of compounds as potential medicinal agents.

## Experimental Section

**Crystallographic data**: Deposition numbers 2048088, 2048089, 2048090, 2048091, 2048092, 2048093, 2048094, 2048095, 2048096, and 2048097 contain the supplementary crystallographic data for this paper. These data are provided free of charge by the joint Cambridge Crystallographic Data Centre and Fachinformationszentrum Karlsruhe Access Structures service.

## Conflict of interest

The authors declare no conflict of interest.

## Supporting information

As a service to our authors and readers, this journal provides supporting information supplied by the authors. Such materials are peer reviewed and may be re‐organized for online delivery, but are not copy‐edited or typeset. Technical support issues arising from supporting information (other than missing files) should be addressed to the authors.

Supplementary
