# Supplementary material for: A Cytotoxic Bis(1,2,3‐triazol‐5‐ylidene)carbazolide Gold(III) Complex Targets DNA by Partial Intercalation
Source: Chemistry. 2021 May 17;27(32):8295–307. doi: 10.1002/chem.202100598 (PMC8251726; doi:10.1002/chem.202100598)
Supplement: Supplementary file 1 — Supplementary [file CHEM-27-8295-s001.pdf]

Table of Contents

General considerations **S2**

Synthetic methodologies **S3**

*1.1 Synthesis of triazoles **P1–P4*** *S4*

*1.2 Synthesis of triazolium salts, **L1–L3, L5^H*** *S12*

*1.3 NMR spectra of triazolium salts, **L1, L2 and L5*** *S14*

1.4 Synthesis of metal complexes *S19*

Single-crystal X-ray diffraction data **S32**

*1.5 Crystal structure data for the triazoles, **P1, P4** and the triazolium salt, **L2*** *S32*

*1.6 Crystal structures of the silver(I) complexes **1a** and **2*** *S34*

*1.7 Crystal structures of the gold(I) complexes **1b, 1c and 4a*** *S37*

*1.8 Crystal structure data for the gold(III) complexes, **3b** and **4b*** *S39*

Cell maintenance and in-vitro procedures **S40**

1.9 Seeding for in-vitro procedures *S40*

1.10 Crystal violet assay *S40*

1.11 Preliminary screening of L3, L4, 3a, 3b , 4a and 4b *S41*

*1.12 Dose-response curves of **L4** and **4b*** *S42*

*1.13 Stability of **4b** in DMSO-d_6* *S43*

Bioinorganic methodologies **S43**

*1.14 Electronic structure of **4b*** *S43*

*1.15 Coordinates and single point energy of **4b*** *S47*

1.16 Stability studies with GSH *S48*

1.17 UV-vis spectroscopy *S50*

1.18 Electrophoretic mobility studies *S51*

1.19 Viscosity measurements *S53*

1.20 CD and LD spectra *S54*

1.21 Macromolecular simulations (docking with Glide) *S57*

1.22 Predictive modelling (docking with Chimera) *S63*

References **S65**

General considerations

Materials. All synthetic procedures sensitive to air and moisture were conducted under inert atmosphere (either under nitrogen or argon gas) using standard Schlenk or vacuum line techniques. Air sensitive solids were stored and handled in a PureLab HE glovebox. All synthetic procedures resulting in novel compounds were performed at least twice to confirm reproducibility. Anhydrous tetrahydrofuran, diethyl ether, toluene and *n*-hexane were distilled over sodium metal under N_2 (g). Dichloromethane and acetonitrile were dried by distillation over CaH_2, under N_2 (g). Triethylamine was purified by distillation over potassium hydroxide. Deuterated dimethyl sulfoxide was stored in a Schlenk tube over activated 4 Å molecular sieves. All other reagents were commercially available and used without any purification. The carbazole precursors 3,6-di(*tert*-butyl)-9*H*-carbazole,[1] 3,6-di(*tert*-butyl)-1,8-dibromo-9*H*-carbazole,[2] 3,6-di(*tert*-butyl)-1,8-bis[(trimethylsilyl)ethynyl]-9*H*-carbazole,[3] 3,6-di(*tert*-butyl)-1,8-bisethynyl-9*H*-carbazole,[3] 1,8-dibromo-9*H*-carbazole,[4] 1,8-bis[(trimethylsilyl)ethynyl]-9*H*-carbazole,[3] 1,8-diethynyl-9*H*-carbazole,[3] 1,3-bis(2,6-diisopropylphenyl)triaz-1-ene,[5,6] *tert*-butyl hypochlorite,[7] dichloro iodobenzene,[8] chloro(tetrahydrothiophene) gold(I),[9] and Meerwein's salt, triethyloxonium tetrafluoroborate[10] were synthesized according to literature procedures as well as the triazolium salt, **L3** and the corresponding gold(I) complex, **3a**.[11,12] All other reagents were commercially available and used without any purification. Caution! Trimethylsilylazide is potentially explosive and should be handled with care

Instrumentation. Nuclear magnetic resonance (NMR) spectra were acquired on either a Bruker Avance-III-300, operating at 300.13 MHz for ^{1}H, 75.47 MHz for ^{13}C, 121.49 MHz for ^{31}P and 282.40 MHz for ^{19}F, or a Bruker Avance-III-400, operating at 400.21 MHz for ^{1}H, 100.64 MHz for ^{13}C, 162.01 MHz for ^{31}P and 376.57 MHz for ^{19}F, or a Avance-III-500, operating at 500.13 MHz for ^{1}H, 125.31 MHz for ^{13}C, 202.46 MHz for ^{31}P and 470.59 MHz for ^{19}F, spectrometer. Standard Bruker pulse programs at 298 K were used in the experiments. Chemical shifts are reported in δ (in ppm) relative to the deuterated solvent signal. For $CDCl_3$, C_6D_6, CD_3CN and $(CD_3)_2SO$ the δ H was referenced at 7.26, 7.16, 1.94 and 2.50 ppm, respectively and the δ ^{13}C {^{1}H} was referenced at 77.16 128.1, 118.26 and 39.52 ppm, respectively. The spectra were analysed with MestReNova Software (9.0.1). The spectral coupling patterns are abbreviated as follows: s - singlet; d - double; t - triplet; q - quartet; sept – septet; m – multiplet; br s – broad signal. Proton coupling constants (*J*) are given in hertz (Hz). An asterisk (*) denotes solvent impurities in the NMR spectra[13] and quaternary carbons are represented by the symbol C_q in ^{13}C {^{1}H} NMR spectra. The multiplicities of the ^{13}C signal in ^{13}C {^{1}H} NMR spectra were deduced from DEPT-135 {^{1}H} experiments. Assignment of non-quaternary carbons were confirmed with 2D heteronuclear (^{13}C-^{1}H) single quantum coherence (HSQC) experiments. Single crystal X-ray structure analyses were performed on a three-circle Bruker Apex II-CCD X-ray diffractometer. Crystals were mounted under oil on nylon loops and the crystals were kept at 173.15 K during data collection. Using Olex2,[14] the structures were solved with the ShelXT[15] structure solution program using intrinsic phasing

and refined with ShelXL[16] refinement package using least squares minimization. Mass spectral analyses were performed on a Bruker Compact Q-TOF high resolution mass spectrometer by direct infusion at a flow rate of 5 µL/min for 1 min using Bruker Daltronics HyStar 3.2 SR4 software and 5 mM sodium formate for calibration. Positive electrospray was employed as ionization technique over a m/z range of 150-3000. Samples for measurements were either prepared in Merck Millipore® purified water or HPLC-grade acetonitrile. Chromatograms were analysed with Bruker Compass DataAnalysis software (Version 4.3) and possible molecular ion adducts were identified with the aid of the mass spectrometry calculator developed by Fiehn Lab which is based on the spectral data reported by Siegel et al.[17] Elemental analyses were carried out using an Elementar varioELcube CHNS-O analyser. Melting points were measured using a Stuart SMP10 melting point apparatus. Absorbance experiments were conducted using either a Specord 210® Plus (Analytik-Jena) using WinASPECT Plus version 4.2.0.0 equipped with a double 8-cell changer unit and a Peltier thermostat system or a PerkinElmer UV-vis Lambda 365 spectrometer fitted with a Peltier Temp. multicell unit and Peltier controller. Quartz cuvettes with 10 mm pathlength were used and measurements were done at either 23 or 37 °C. OriginPro 2020b was used to analyse all UV-vis spectra. All viscometry measurements were carried out in triplicate using an Anton Paar Lovis 2000 M rolling ball microviscometer at 37 °C. A Bio-Rad Mini-Sub-Cell GT® Agarose Gel Electrophoresis System was used for all electrophoresis experiments at ambient temperature, typically at 65 V and at least in triplicate. Gels were visualized using a G:Box Chemi XRQ gel doc system (Syngene) with mid-wave UV transillumination and a UV filter (GeneSys 1.4.6.0).

Synthetic methodologies

The N3-alkylated analogues (**L1** and **L2**, Scheme S1) of **L3** were prepared starting from the commercially available 9*H*-carbazole precursor using modified literature procedures. The 3- and 6-positions of the carbazole were functionalised by *tert*-butyl groups that provide a dual function: as protecting group for the 3,6-postions, which can be removed by reverse Friedel-Crafts alkylation to afford the 1,8-disubstituted carbazole,[4] or if left as carbazole substituents, sterically hindered analogues allowing for comparative studies. A sequence of synthetic modifications allowed for the synthesis of the respective terminal alkynes i.e., 1,8-diethynyl-9*H*-carbazole and 3,6-*tert*-butyl-1,8-diethynyl-9*H*-carbazole to combine with phenyl azide in the CuAAC reaction to produce the 1,4-disubstituted-1,2,3-triazoles, **P1** and **P2**, also adapted from previous literature reports.[18] Alkylation on both N3-positions of the triazoles afforded the triazolium salts, **L1** and **L2** as precursors for metalation.

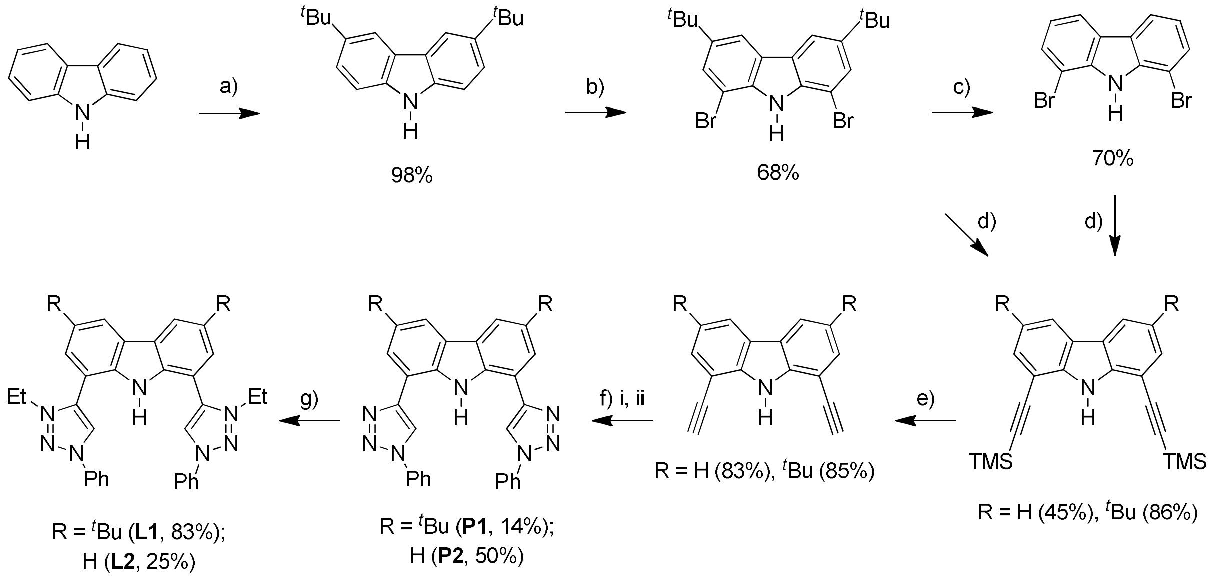

Scheme S1. The synthesis of the 1,4-disubstitued-1,2,3-triazoles, **P1** and **P2** and subsequent alkylation to **L1** and **L2**, respectively from 9*H*-carbazole. The reaction conditions were as follows: a) 3 eq ^{t}BuCl, 3 eq $ZnCl_2$, nitromethane, rt, 1d; b) 2.2 eq Br_2, AcOH, rt, 2d; c) 3 eq $AlCl_3$, toluene, rt, 4 hr; d) 5% $Pd(PPh_3)_2Cl_2$, 6% CuI, 2.5 eq ethynyltrimethylsilane, Et_3N, 75°C, 2 d; e) 6 eq K_2CO_3, MeOH, 75°C, 30 min; f) i) 3 eq aniline, 1.5 eq ^{t}BuONO, 1.2 eq $TMSN_3$, CH_3CN, 0°C, 2 h; ii) 0.8 eq $CuSO_4.5H_2O$, 1.6 eq Na-ascorbate, 3 eq K_2CO_3, 2 eq pyridine, THF/H_2O, rt, 4 d; g) 6 eq Et_3OBF_4, CH_2Cl_2, 0°C, 4 d.

1.1 Synthesis of triazoles P1–P4

Synthesis and characterization of *3,6-di(tert-butyl)-1,8-bis-(3-phenyl-1,2,3-triazole)-9H-carbazole* (P1)

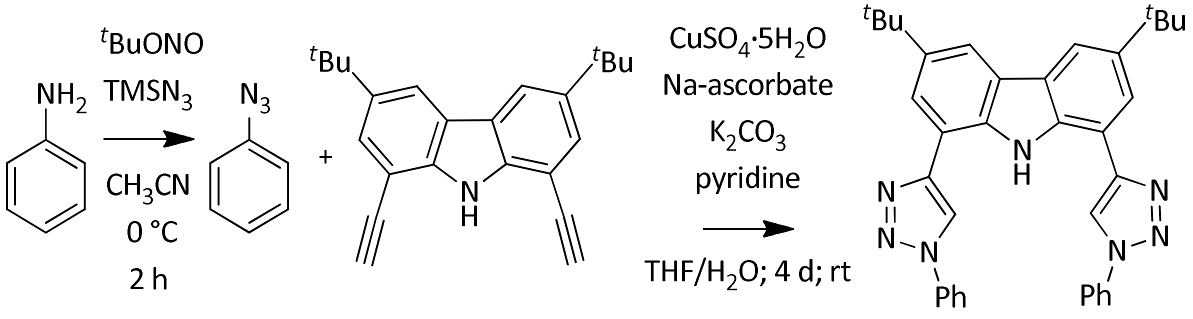

P1 (50%)

Scheme S2. Synthesis of *3,6-di(tert-butyl)-1,8-bis-(3-phenyl-1,2,3-triazole)-9H-carbazole* (**P1**).

To a solution of aniline (1.7 g, 18 mmol) in 20 mL CH_3CN at −20°C, was added dropwise *tert*-butyl nitrite (2.8 g, 27 mmol) followed by trimethylsilyl azide (2.5 g, 22 mmol). After the reaction was stirred for 2 hours at room temperature, 3,6-di(*tert*-butyl)-1,8-diethynyl-9*H*-carbazole (1.0 g, 3.1 mmol) dissolved in 20 mL THF was added, followed by potassium carbonate (1.3 g, 9.1 mmol), pyridine (0.48 g, 6.1 mmol), 20 mL aqueous copper(II) sulphate pentahydrate (0.61 g, 2.4 mmol) solution and 20 mL aqueous sodium ascorbate (0.97 g, 4.9 mmol) solution. The reaction proceeded for 4 days and was quenched with 0.1 M EDTA/NH_4OH. The organic phase was extracted with CH_2Cl_2, dried over magnesium sulphate,

and concentrated to a dark red oil, which was further purified by flash chromatography (eluted with CH_2Cl_2). The solvent was evaporated and the oily solid was stirred overnight in diethyl ether and filtered, affording a bright yellow solid. Crystals suitable for XRD analysis were grown from the NMR sample in $CDCl_3$. Yield: 0.85 g (50%). ^{1}H NMR (300 MHz, $CDCl_3$) δ 11.89 (s, 1H, NH), 8.48 (s, 2H, trz-CH), 8.19 (d, *J* = 0.9 Hz, 2H, Ar-$CH_{carbazole}$, H-1_a), 7.92 (d, *J* = 7.7 Hz, 4H, Ar-CH_{Ph}, H-2_a), 7.83 (d, *J* = 1.5 Hz, 2H, Ar-$CH_{carbazole}$, H-1_b), 7.58 (dd, *J* = 7.7, 7.7 Hz, 4H, Ar-CH_{Ph}, H-2_b), 7.49 (t, *J* = 7.3 Hz, 2H, Ar-CH_{Ph}, H-2_c), 1.54 (s, 18H, ^{t}Bu-CH_3). ^{13}C {^{1}H} NMR (75 MHz, $CDCl_3$) δ 148.4, 142.1, 137.4, 136.1 (all Ar-C_q), 129.9 (Ar-CH_{Ph}, C-2_b), 128.9 (Ar-CH_{Ph}, C-2_c), 124.2 (Ar-C_q), 120.8 (Ar-CH_{Ph}, C-2_a), 120.6 (Ar-$CH_{carbazole}$, C-1_b), 117.4 (trz-CH), 116.9 (Ar-$CH_{carbazole}$, C-1_a), 112.6 (Ar-C_q), 34.9 (^{t}Bu-C_q), 32.3 (^{t}Bu-CH_3). Anal. Calcd for $C_{36}H_{35}N_7$: C 76.43, H 6.24, N 17.33. Found: C 76.81, H 6.25, N 17.69. ESI-(+)MS (Q-TOF)(positive mode, m/z): calcd for $[M+H]^+$: 566.3027. Found: 566.3016.

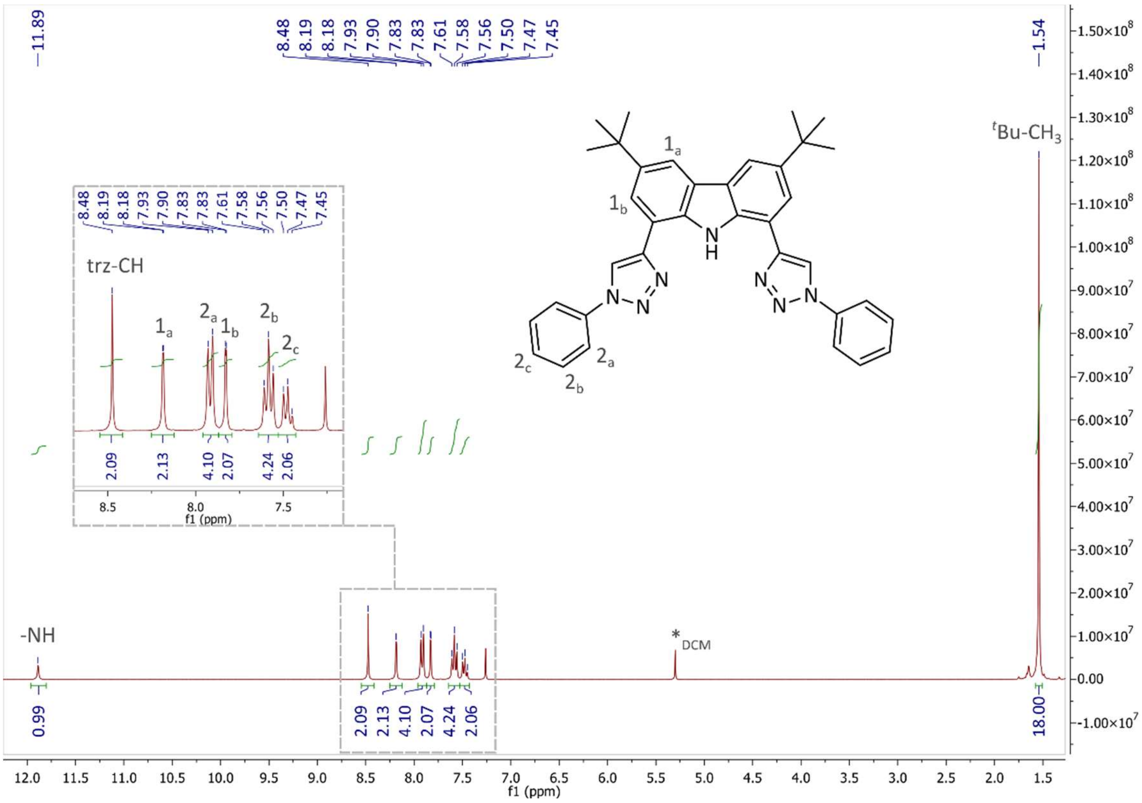

Figure S1.^{1}H NMR spectrum of **P1** in $CDCl_3$.

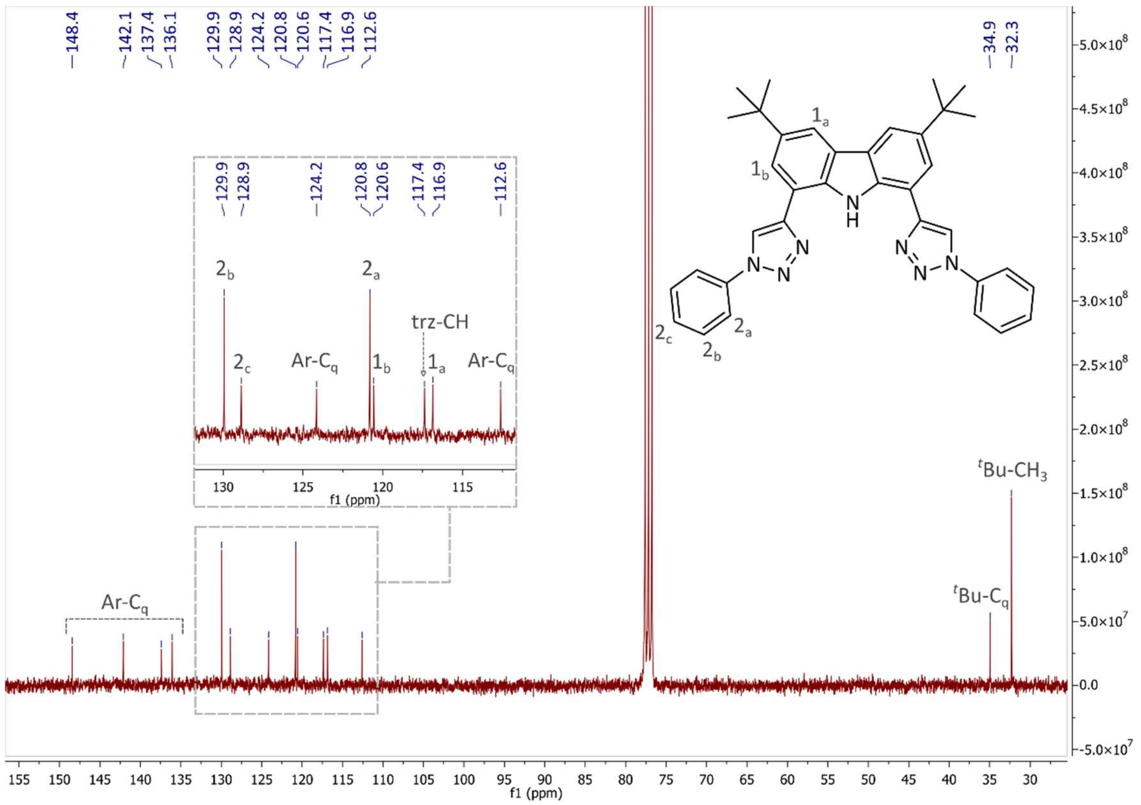

Figure S2. The ^{13}C {^{1}H} NMR spectrum of **P1** in $CDCl_3$.

Synthesis and characterization of *1,8-bis-(3-phenyl-1,2,3-triazole)-9H-carbazole* (P2)

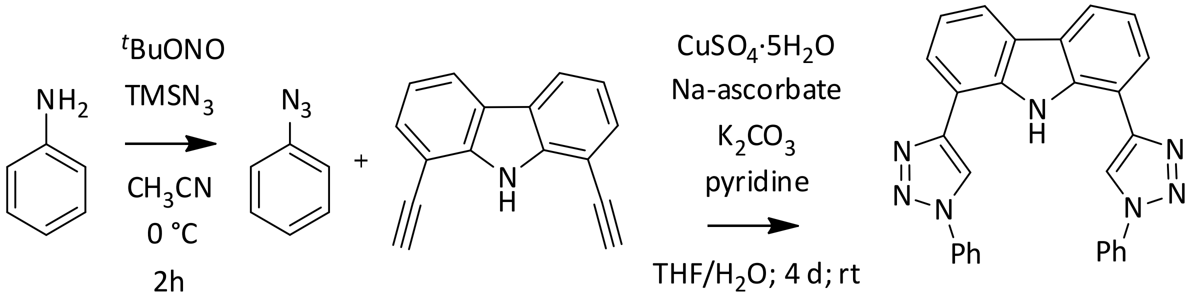

P2 (14%)

Scheme S3. The synthesis of *1,8-bis-(3-phenyl-1,2,3-triazole)-9H-carbazole* (**P2**).

To a solution of aniline (2.7 g, 29 mmol) in 20 mL CH_3CN at −20 °C, was added dropwise *tert*-butyl nitrite (4.3 g, 42 mmol) and then trimethylsilyl azide (3.8 g, 33 mmol). After the reaction was stirred for 2 hours at room temperature, 1,8-diethynyl-9*H*-carbazole (1.0 g, 4.7 mmol) dissolved in 20 mL THF was added to the reaction mixture followed by potassium carbonate (1.9 g, 14 mmol), pyridine (0.74 g, 9.3 mmol), 20 mL aqueous copper(II)sulphate pentahydrate (0.93 g, 3.7 mmol) solution and 20 mL aqueous sodium ascorbate (1.5 g, 7.4 mmol) solution. The reaction continued for 4 days and was quenched with 0.1 M EDTA/NH_4OH. The suspension was filtered and the solid dissolved in CH_2Cl_2. The organic phase was extracted with CH_2Cl_2, dried over magnesium sulphate and concentrated to a dark red oil, which was further purified by flash chromatography (eluted with CH_2Cl_2), evaporated and stirred overnight in diethyl ether and filtered, affording a bright yellow solid. Yield: 0.3 g (14%). M.p.

260 °C (decomp.). ^{1}H NMR (300 MHz, $(CD_3)_2SO$) δ 12.32 (s, 1H, NH), 9.63 (s, 2H, trz-CH), 8.28 (d, *J* = 7.6 Hz, 2H, Ar-$CH_{carbazole}$, H-1_a), 8.10 (d, *J* = 7.5 Hz, 4H, Ar-CH_{Ph}, H-2_a), 8.02 (d, *J* = 7.5 Hz, 2H, Ar-$CH_{carbazole}$, H-1_b), 7.70 (dd, *J* = 7.8, 7.8 Hz, 4H, Ar-CH_{Ph}, H-2_b), 7.57 (t, *J* = 7.4 Hz, 2H, Ar-CH_{Ph}, H-2_c), 7.40 (dd, *J* = 7.6, 7.6 Hz, 2H, Ar-$CH_{carbazole}$, H-1_c). ^{13}C {^{1}H} NMR (75 MHz, $(CD_3)_2SO$) δ 147.1, 136.7, 136.0 (all Ar-C_q), 130.0 (Ar-CH_{Ph}, C-2_b), 129.0 (Ar-CH_{Ph}, C-2_c), 123.4 (Ar-C_q), 123.1 (Ar-$CH_{carbazole}$, C-1_b), 120.5 (Ar-$CH_{carbazole}$, C-1_a), 120.2 (Ar-CH_{Ph}, C-2_a), 119.8 (trz-CH), 119.6 (Ar-$CH_{carbazole}$, C-1_c), 113.0 (Ar-C_q). Anal. Calcd for $C_{28}H_{19}N_7$ (+ 0.3 eq DMSO): C 72.02, H 4.40, N 20.56. Found: C 72.37, H 4.53, N 20.47. ESI-(+)MS (Q-TOF) (positive mode, m/z): calcd for $[M+H]^+$: 453.1780. Found: 453.1782.

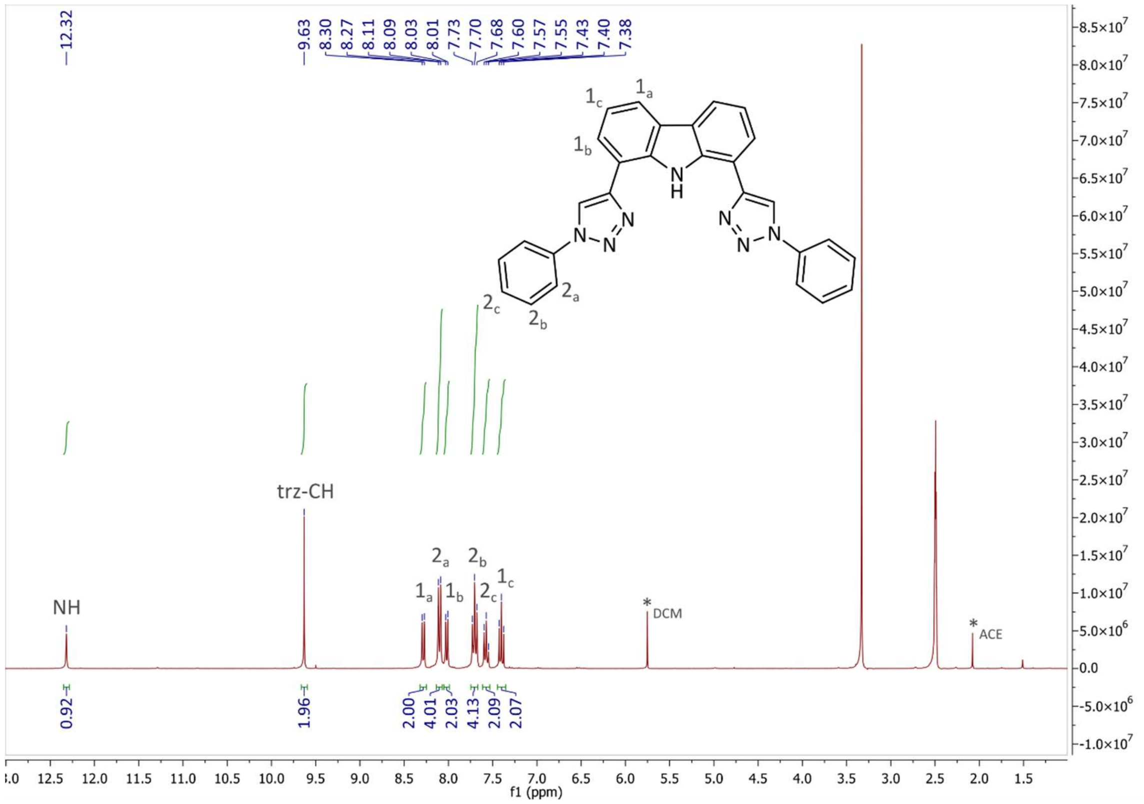

Figure S3. ^{1}H NMR spectrum of **P2** in $(CD_3)_2SO$.

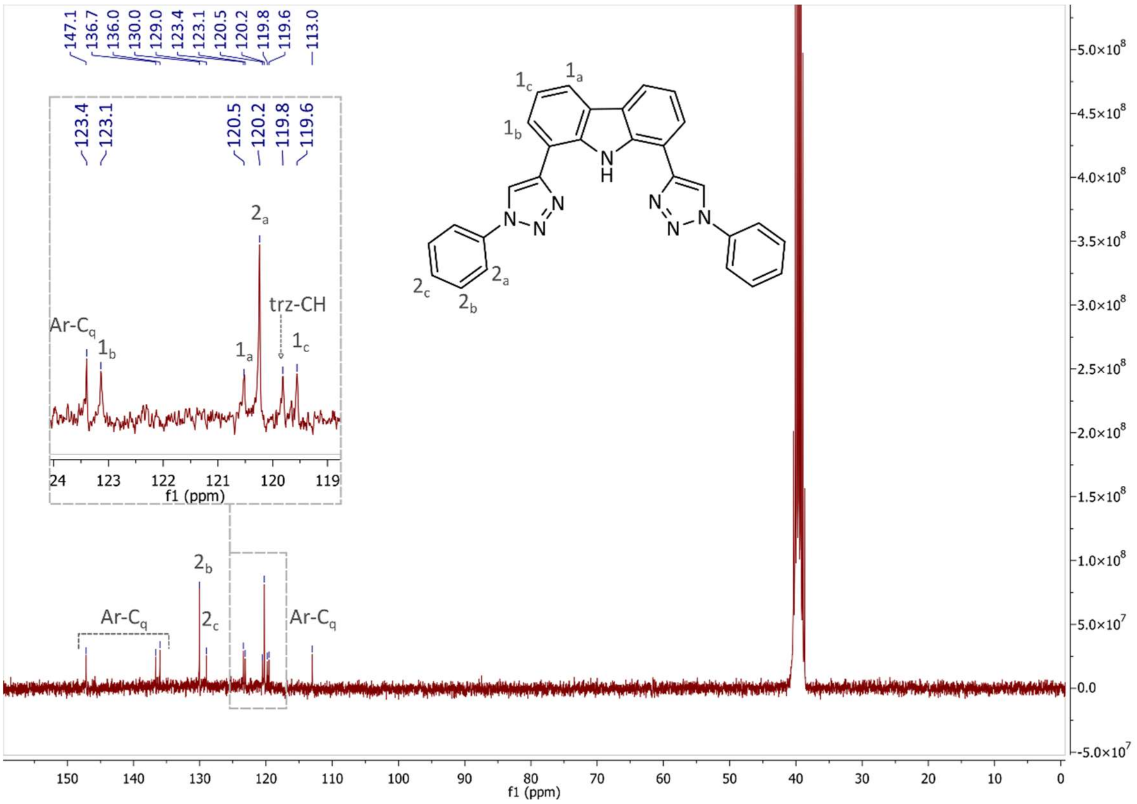

Figure S4. The $^{13}C\ \{^1H\}$ NMR spectrum of **P2** in $(CD_3)_2SO$.

Synthesis and characterization of 3,6-di(tert-butyl)-1,8-bis-(3-diisopropylphenyl-1,2,3-triazole)-9H-carbazole (P3)

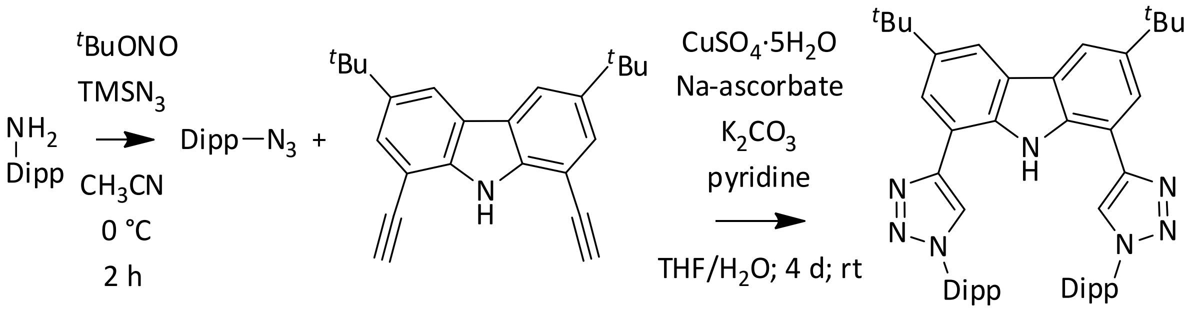

Scheme S4. Synthesis of *3,6-di(tert-butyl)-1,8-bis-(3-diisopropylphenyl-1,2,3-triazole)-9H-carbazole* (**P3**).

To a solution of 2,6-diisopropylaniline (2.4 g, 14 mmol) in 20 mL CH_3CN at -20 °C, was added dropwise *tert*-butyl nitrite (2.0 g, 19 mmol) followed by trimethylsilyl azide (1.8 g, 16 mmol). After the reaction was stirred for 2 hours at room temperature, 3,6-di(*tert*-butyl)-1,8-diethynyl-9H-carbazole (1.1 g, 3.4 mmol) dissolved in 20 mL THF was added, followed by potassium carbonate (1.6 g, 11 mmol), pyridine (0.59 g, 7.5 mmol), 20 mL aqueous copper(II)sulphate pentahydrate (0.73 g, 2.9 mmol) solution and 20 mL aqueous sodium ascorbate (1.2 g, 5.8 mmol) solution. The reaction continued for 4 days and was quenched

with 0.1 M EDTA/NH_4OH. The organic phase was extracted with CH_2Cl_2, dried over magnesium sulphate, and concentrated to a red foam. The crude product was further purified by flash chromatography (eluted with CH_2Cl_2), after which the solvent was evaporated and the product was stirred for 20 minutes in hexane and filtered, affording a white solid. Yield: 2.2 g (86%). ^{1}H NMR (300 MHz, $CDCl_3$) δ 11.72 (s, 1H, NH), 8.20 (d, J = 1.8 Hz, 2H, Ar-$CH_{carbazole}$, H-1_a), 8.16 (s, 2H, trz-CH), 7.87 (d, J = 1.8 Hz, 2H, Ar-$CH_{carbazole}$, H-1_b), 7.52 (t, J = 7.7 Hz, 2H, Ar-CH_{Dipp}, H-2_a), 7.32 (d, J = 7.7 Hz, 4H, Ar-CH_{Dipp}, H-2_b), 2.43 (sept, J = 6.5 Hz, 4H, ^{i}Pr-CH, H-2_c), 1.54 (s, 18H, ^{t}Bu-CH_3), 1.19 (d, J = 6.9 Hz, 24H, ^{i}Pr-CH_3, H-2_d). ^{13}C {^{1}H} NMR (75 MHz, $CDCl_3$) δ 147.3, 146.4, 142.2, 136.0, 133.5 (all Ar-C_q), 130.9 (Ar-CH_{Dipp}, C-2_a), 127.1 (Ar-C_q), 124.3 (Ar-C_q), 124.0 (Ar-CH_{Dipp}, C-2_b), 122.3 (trz-CH), 120.6 (Ar-$CH_{carbazole}$, C-1_b), 116.8 (Ar-$CH_{carbazole}$, C-1_a), 112.7 (Ar-C_q), 35.0 (^{t}Bu-C_q), 32.3 (^{t}Bu-CH_3), 28.5 (^{i}Pr-CH, C-2_c), 24.5 (^{i}Pr-CH_3, C-2_d), 24.4 (^{i}Pr-CH_3, C-2_d). ESI-(+)MS (Q-TOF) (positive mode, m/z): calcd for $[M+H]^+$: 734.4904. Found: 734.4890.

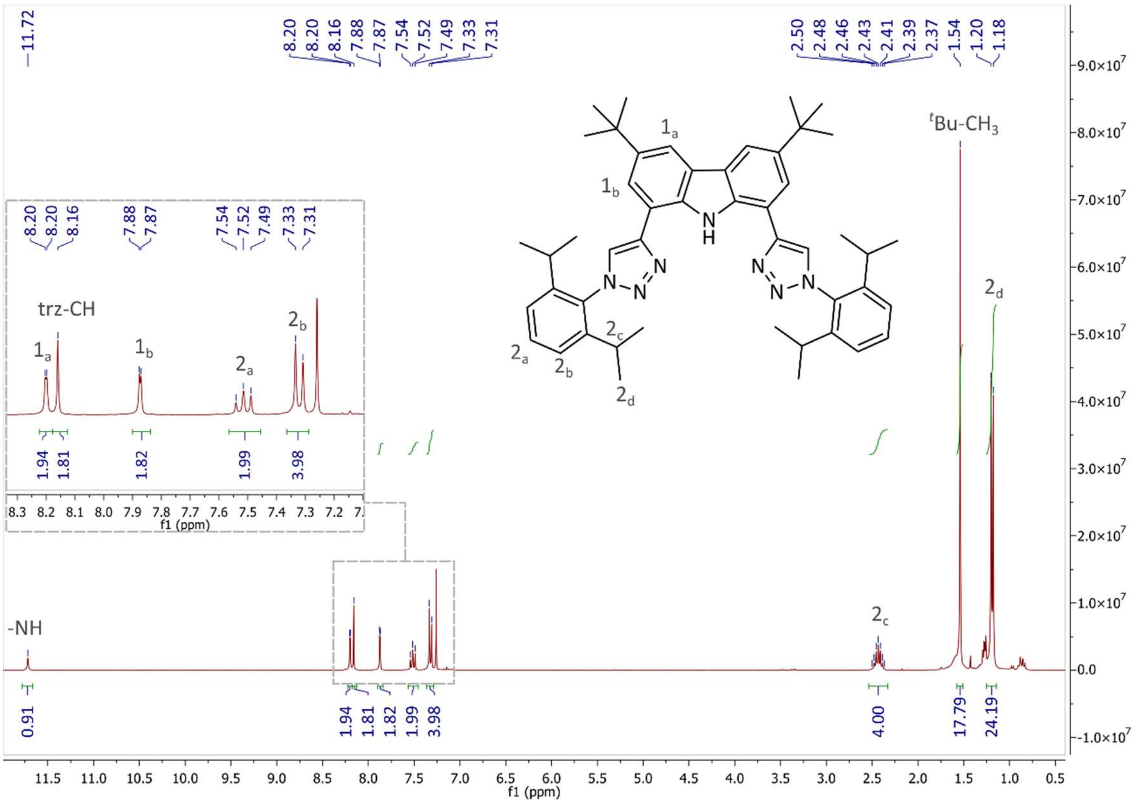

Figure S5. ^{1}H NMR spectrum of **P3** in $CDCl_3$.

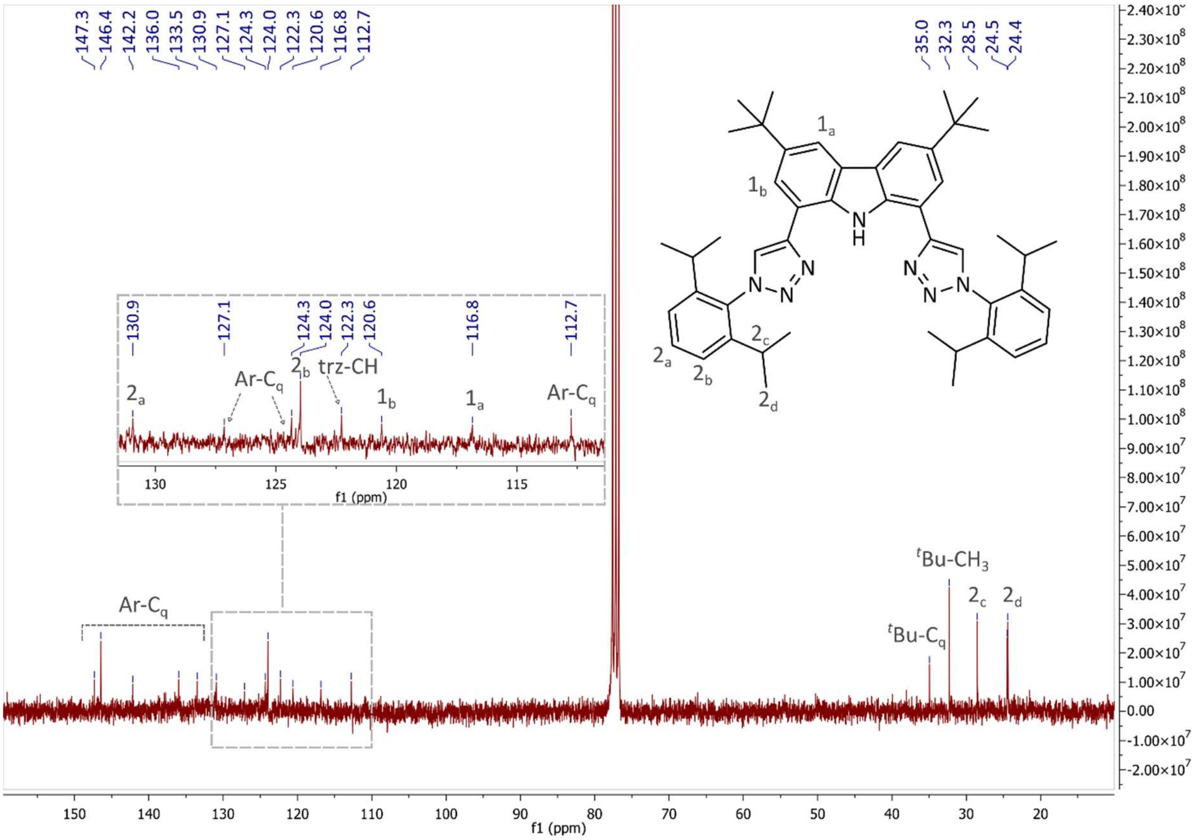

Figure S6. The $^{13}C\ \{^{1}H\}$ NMR spectrum of **P3** in $CDCl_3$.

Synthesis and characterization of *1,8-bis-(3-diisopropylphenyl-1,2,3-triazole)-9H-carbazole* (P4)

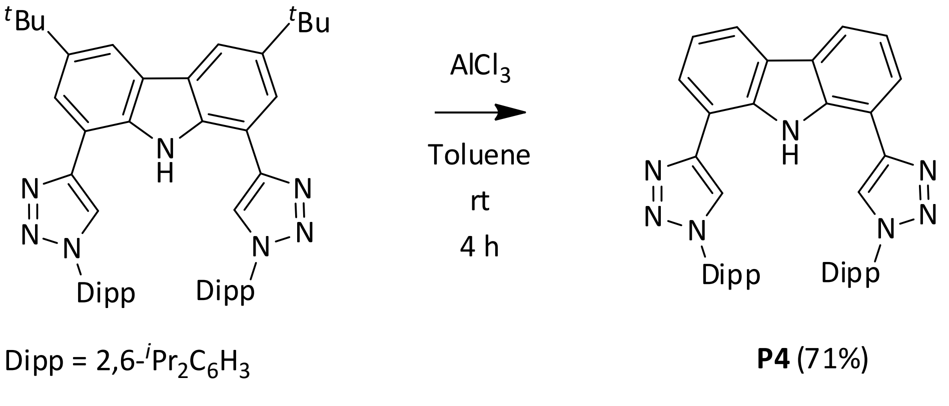

Scheme S5. Synthesis of *1,8-bis-(3-diisopropylphenyl-1,2,3-triazole)-9H-carbazole* (**P4**).

The synthesis of **P4** did not follow the conventional click route. Instead it was prepared by the deprotection of *tert*-butyl groups described for non-triazole aromatic systems.[3,19–22] **P3** (2.0 g, 2.7 mmol) was dissolved in 50 mL of anhydrous toluene followed by the addition of anhydrous aluminium chloride (9.2 g, 69 mmol) in small amounts under a nitrogen atmosphere. The reaction mixture turned dark green and was stirred for 4 hours, after which the colour changed to orange red. The reaction was slowly quenched with water and the crude product was subsequently extracted with CH_2Cl_2, dried over magnesium sulphate, and

evaporated. The residue was stirred in hexane overnight and filtered, affording the product as an off-white solid. Crystals suitable for single crystal X-ray diffraction were grown from the slow evaporation of diethyl ether into a concentrated chloroform solution. Yield: 1.2 g (71%). ^{1}H NMR (300 MHz, $CDCl_3$) δ 12.33 (s, 1H, NH), 8.19 (d, *J* = 7.7 Hz, 2H, Ar-$CH_{carbazole}$, H-1_a), 8.13 (s, 2H, trz-CH), 7.82 (d, *J* = 7.4 Hz, 2H, Ar-$CH_{carbazole}$, H-1_b), 7.53 (t, *J* = 7.8 Hz, Ar-CH_{Dipp}, H-2_a), 7.36 (dd, *J* = 7.6, 7.6 Hz, 2H, Ar-$CH_{carbazole}$, H-1_c), 7.33 (d, *J* = 7.8, 4H, Ar-CH_{Dipp}, H-2_b), 2.42 (sept, *J* = 6.8 Hz, 4H, ^{i}Pr-CH, H-2_c), 1.18 (t, *J* = 7.2 Hz, 24H, ^{i}Pr-CH_3, H-2_d). ^{13}C {^{1}H} NMR (75 MHz, $CDCl_3$) δ 147.1, 146.4, 137.7, 137.3, 133.4 (all Ar-C_q), 131.0 (Ar-CH_{Dipp}, C-2_a), 124.3 (Ar-C_q), 124.0 (Ar-CH_{Dipp}, C-2_b), 122.9 (Ar-$CH_{carbazole}$, C-1_c), 122.4 (Ar-$CH_{carbazole}$, C-1_b), 120.4 (trz-CH), 119.3 (Ar-$CH_{carbazole}$, C-1_a), 113.6 (Ar-C_q), 28.5 (^{i}Pr-CH, C-2_c), 24.4 (^{i}Pr-CH_3, C-2_c), 24.3 (^{i}Pr-CH_3, C-2_d). Anal. Calcd for $C_{40}H_{43}N_7$ (+ 0.1 eq hex): C 77.35, H 7.10, N 15.55. Found: C 77.77, H 7.14, N 15.18. ESI-(+)MS (Q-TOF) (positive mode, m/z): calcd for $[M+H]^+$: 622.3652. Found: 622.3649.

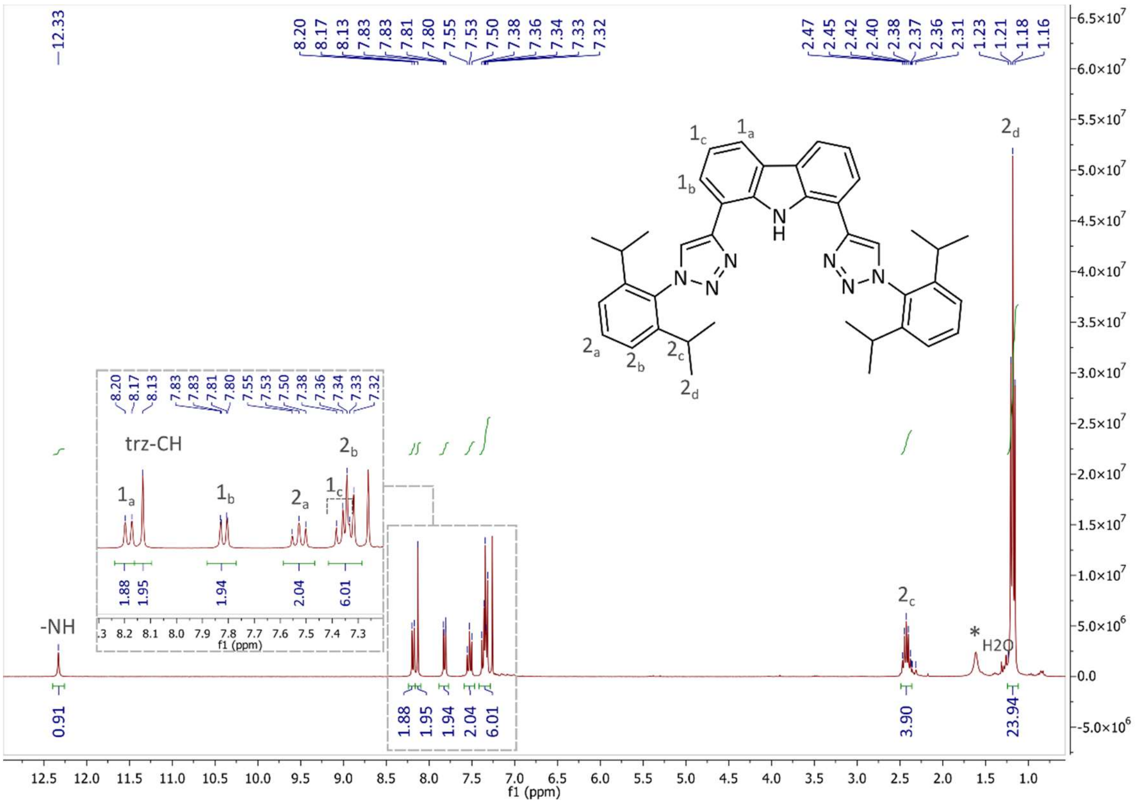

Figure S7. ^{1}H NMR spectrum of **P4** in $CDCl_3$.

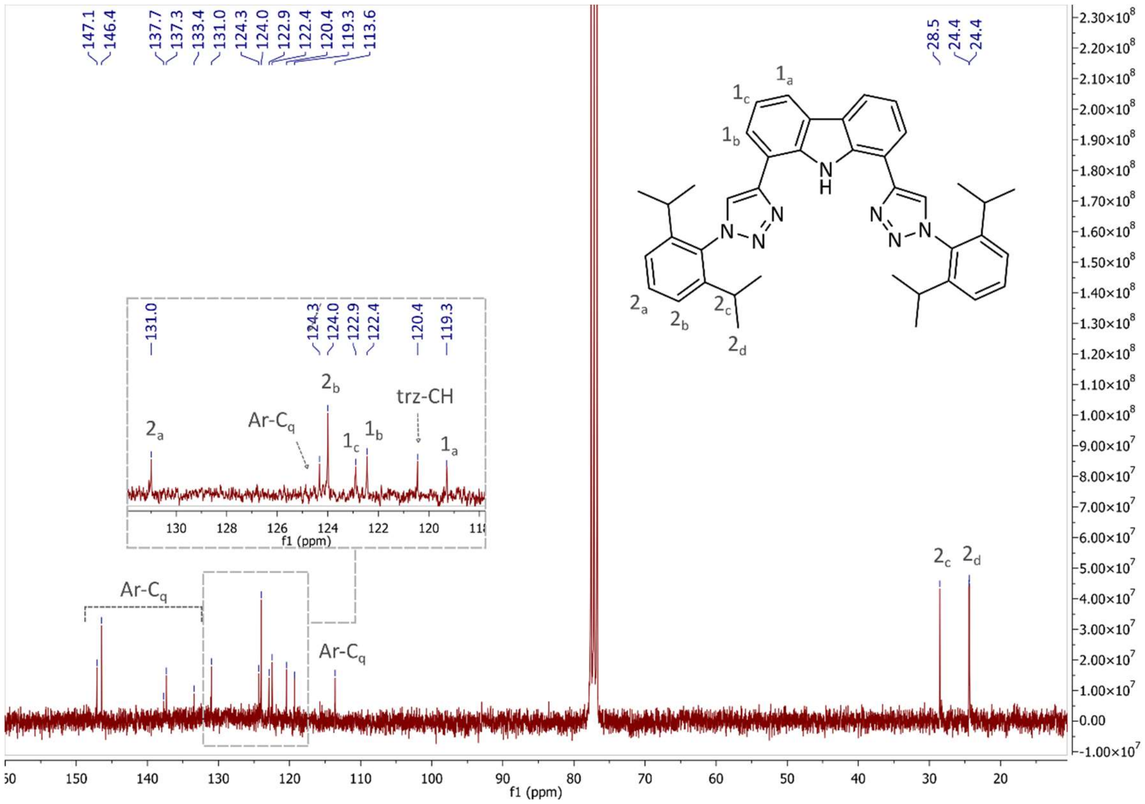

Figure S8. The $^{13}C\ \{^1H\}$ NMR spectrum of **P4** in $CDCl_3$.

1.2 Synthesis of triazolium salts, L1–L3, L5[H]

The precursor salts, 3,6-di(R)-1,8-bis-(1-ethyl-3-Ar-1,2,3-triazolium)-9*H*-carbazole tetrafluoroborate(III), where R = H, *tert*-butyl and Ar = phenyl, diisopropylphenyl, were synthesized from their corresponding triazoles.

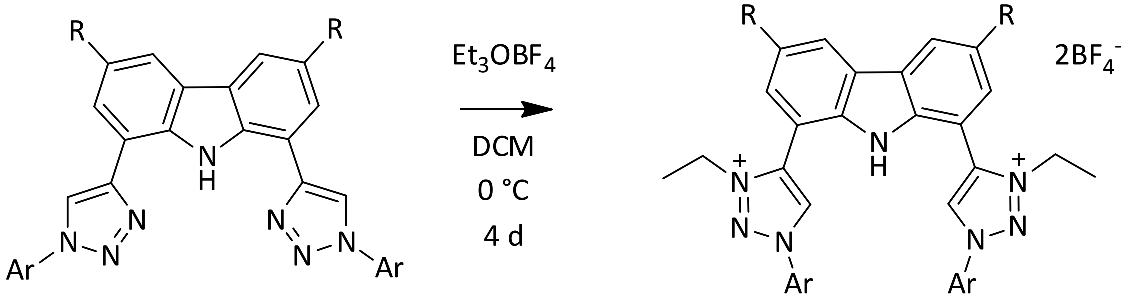

L1: R = ^{t}Bu, Ar = Ph (83%)
L2: R = H, Ar = Ph (25%)
L5: R = ^{t}Bu, Ar = Dipp (2,6-$^iPr_2C_6H_3$) (25%)

Scheme S6. The preparation of the N3-alkylated triazolium salts, **L1**, **L2** and **L5**.

To a solution of the appropriate triazole derivative in anhydrous CH_2Cl_2 (20 mL), a solution of 6 equivalents of triethyloxonium tetrafluoroborate in CH_2Cl_2 (10 mL) was added at −30 °C and was left to reach room temperature slowly overnight, after which the reaction was stirred for an additional three days. The reaction was quenched with methanol and the solvents

evaporated. The residue was dissolved in minimum acetone (4 mL) and then diethyl ether (40 mL) and the suspension was stirred for 1 h. The precipitate was filtered and dried under vacuum.

3,6-tert-butyl-1,8-bis-(1-ethyl-3-phenyl-1,2,3-triazolium)-9H-carbazole tetrafluoroborate(III), **L1**: White powder. Yield: 1.75 g (83%). ^{1}H NMR (300 MHz, $CDCl_3$) δ 9.80 (s, 1H, NH), 9.45 (s, 2H, trz-CH), 8.35 (d, *J* = 1.3 Hz, 2H, Ar-$CH_{carbazole}$, H-1_a), 8.00–7.97 (m, 4H, Ar-CH_{Ph}, H-2_a), 7.56–7.55 (m, 4H, Ar-CH_{Ph}, H-2_b), 7.54–7.53 (m, 2H, Ar-CH_{Ph}, H-2_c), 7.49 (d, *J* = 1.6 Hz, 2H, Ar-$CH_{carbazole}$, H-1_b), 4.62 (q, *J* = 7.3 Hz, 4H, Et-CH_2, H-3_a), 1.71 (t, *J* = 7.3 Hz, 6H, Et-CH_3, H-3_b), 1.50 (s, 18H, ^{t}Bu-CH_3). ^{13}C {^{1}H} NMR (75 MHz, $CDCl_3$) δ 143.4, 140.6, 138.0, 135.4 (all Ar-C_q), 131.7 (Ar-CH_{Ph}, C-2_c), 130.2 (Ar-CH_{Ph}, C-2_b), 128.4 (trz-CH), 124.8 (Ar-$CH_{carbazole}$, C-1_b), 124.7 (Ar-C_q), 122.3 (Ar-CH_{Ph}, C-2_a), 120.3 (Ar-$CH_{carbazole}$, C-1_a), 104.7 (Ar-C_q), 47.8 (Et-CH_2, C-3_a), 35.1 (^{t}Bu-C_q), 32.1 (^{t}Bu-CH_3), 14.4 (Et-CH_3, C-3_b). ^{19}F {^{1}H} NMR (470 MHz, $CDCl_3$) δ -152.36 (d, *J* = 24.45 Hz, BF_4). SI-(+)MS (Q-TOF) (positive mode, m/z): calcd for $[M–2BF_4]^{2+}$: 311.6862. Found: 311.6864.

1,8-bis-(1-ethyl-3-phenyl-1,2,3-triazolium)-9H-carbazole tetrafluoroborate(III), **L2**: White powder. Crystals suitable for XRD analysis were grown from the slow evaporation of diethyl ether into a concentrated DMF solution. Yield: 0.4 g (25%).^{1}H NMR (300 MHz, $(CD_3)_2SO$) δ 11.41 (s, 1H, NH), 10.04 (s, 2H, trz-CH), 8.68 (d, *J* = 7.8 Hz, 2H, Ar-$CH_{carbazole}$, H-1_a), 8.13–8.10 (m, 4H, Ar-CH_{Ph}, H-2_a), 7.85 (d, *J* = 7.5 Hz, 2H, Ar-$CH_{carbazole}$, H-1_b), 7.81–7.79 (m, 6H, Ar-CH_{Ph}, H-2_b + H-2_c), 7.63 (dd, *J* = 7.7, 7.7 Hz, 2H, Ar-$CH_{carbazole}$, H-1_c), 4.73 (q, *J* = 7.2 Hz, 4H, Et-CH_2, H-3_a), 1.53 (t, *J* = 7.2 Hz, 6H, Et-CH_3, H-3_b). ^{13}C {^{1}H} NMR (75 MHz, $(CD_3)_2SO$) δ 139.6, 138.4, 134.7 (all Ar-C_q), 132.1 (Ar-CH_{Ph}, C-2_c), 130.7 (Ar-CH_{Ph}, C-2_b), 128.9 (trz-CH), 128.7 (Ar-$CH_{carbazole}$, C-1_b), 124.7(4) (Ar-C_q), 124.7 (Ar-$CH_{carbazole}$, C-1_a), 123.8 (Ar-C_q), 121. 0 (Ar-CH_{Ph}, C-2_a), 120.5 (Ar-$CH_{carbazole}$, C-1_c), 105.4 (Ar-C_q), 47.8 (Et-CH_2, C-3_a), 13.7 (Et-CH_3, C-3_b). ^{19}F {^{1}H} NMR (376 MHz, $(CD_3)_2SO$) δ -148.35 (d, *J* = 21.2 Hz, BF_4). Anal. Calcd for $C_{36}H_{35}N_7B_2F_8$ (+ 0.15 eq hex): C 56.60, H 4.49, N 14.04. Found: C 57.02, H 4.14, N 14.33. ESI-(+)MS (Q-TOF) (positive mode, m/z): calcd for $[M–2BF_4]^{2+}$: 255.6237. Found: 255.6251

3,6-tert-butyl-1,8-bis-(1-ethyl-3-diisopropylphenyl-1,2,3-triazolium)-9H-carbazole tetrafluoroborate(III), **L5**: White powder. Yield: 0.30 g (25%). ^{1}H NMR (300 MHz, $CDCl_3$) δ 9.77 (s, 1H, NH), 8.90 (s, 2H, trz-CH), 8.38 (d, *J* = 1.7 Hz, 2H, Ar-$CH_{carbazole}$, H-1_a), 7.61 (t, *J* = 7.78 Hz, 2H, Ar-CH_{Dipp}, H-2_a), 7.39 (d, *J* = 1.3 Hz, 2H, Ar-$CH_{carbazole}$, H-1_b), 7.37–7.36 (m, 4H, Ar-CH_{Dipp}, H-2_b), 4.74 (q, *J* = 7.2 Hz, 4H, Et-CH_2, H-3_a), 2.46 (p, *J* = 6.7 Hz, 4H, ^{i}Pr-CH, H-2_c), 1.69 (t, *J* = 7.1 Hz, 6H, Et-CH_3, H-3_b), 1.52 (s, 18H, ^{t}Bu-CH_3), 1.24 (d, *J* = 7.3 Hz, 12H, ^{i}Pr-CH_3, H-2_d), 1.21 (d, *J* = 7.4 Hz, 12H, ^{i}Pr-CH_3, H-2_d). ^{13}C {^{1}H} NMR (75 MHz, $CDCl_3$) δ 146.0, 143.4, 142.1, 137.8 (all Ar-C_q), 132.7 (Ar-CH_{Dipp}, C-2_a), 131.9 (trz-CH), 131.4 (Ar-C_q), 126.1 (Ar-$CH_{carbazole}$, C-1_b), 125.7 (Ar-C_q), 124.8 (Ar-CH_{Dipp}, C-2_b), 121.3 (Ar-$CH_{carbazole}$, C-1_a), 104.8 (Ar-C_q), 49.0 (Et-CH_2, C-3_a), 35.0 (^{t}Bu-C_q), 32.1 (^{t}Bu-CH_3), 28.9 (^{i}Pr-CH, C-2_c), 24.9 (^{i}Pr-CH_3, C-2_d), 23.6 (^{i}Pr-CH_3, C-2_d), 14.5 (Et-CH_3, C-3_b). ^{19}F {^{1}H} NMR (470 MHz, $CDCl_3$) δ -151.98 (d, *J* = 24.3 Hz, BF_4). ESI-(+)MS (Q-TOF) (positive mode, m/z): calcd for $[M–2BF_4]^{2+}$: 395.7801. Found: 395.7804.

1.3 NMR spectra of triazolium salts, L1, L2 and L5

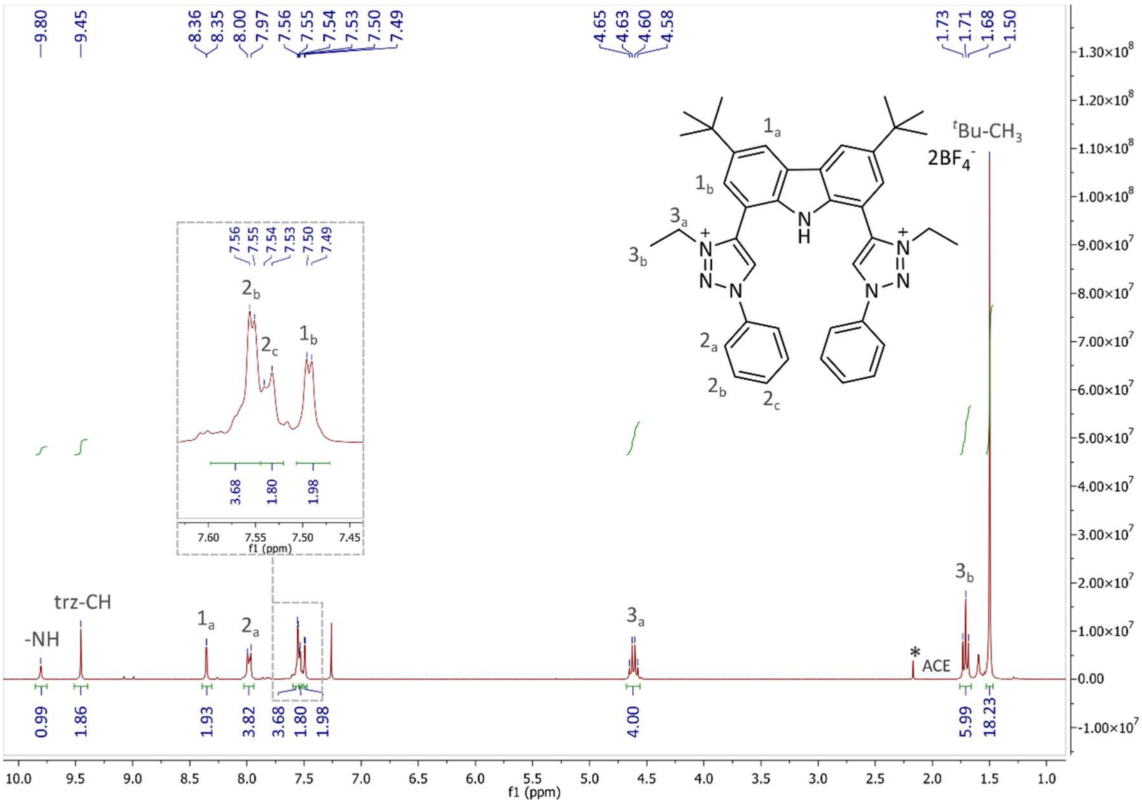

Figure S9. ^{1}H NMR spectrum of **L1** in $CDCl_3$.

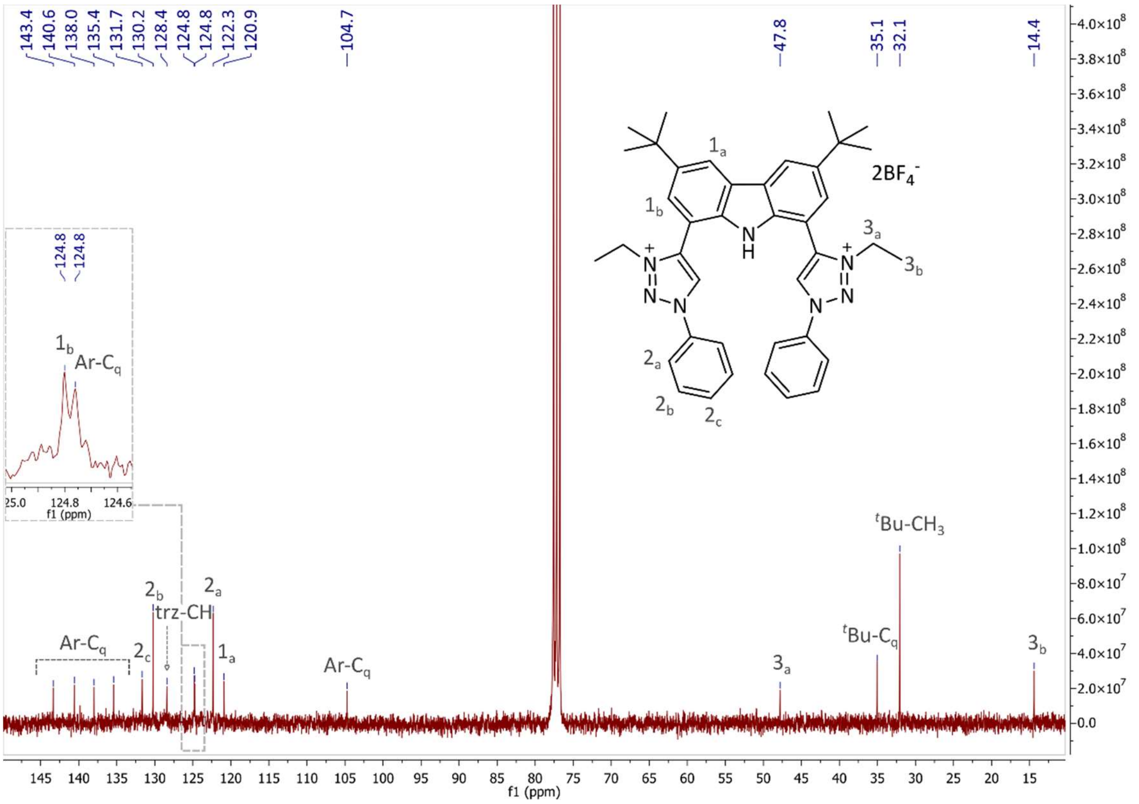

Figure S10. The $^{13}C\ \{^1H\}$ NMR spectrum of **L1** in $CDCl_3$.

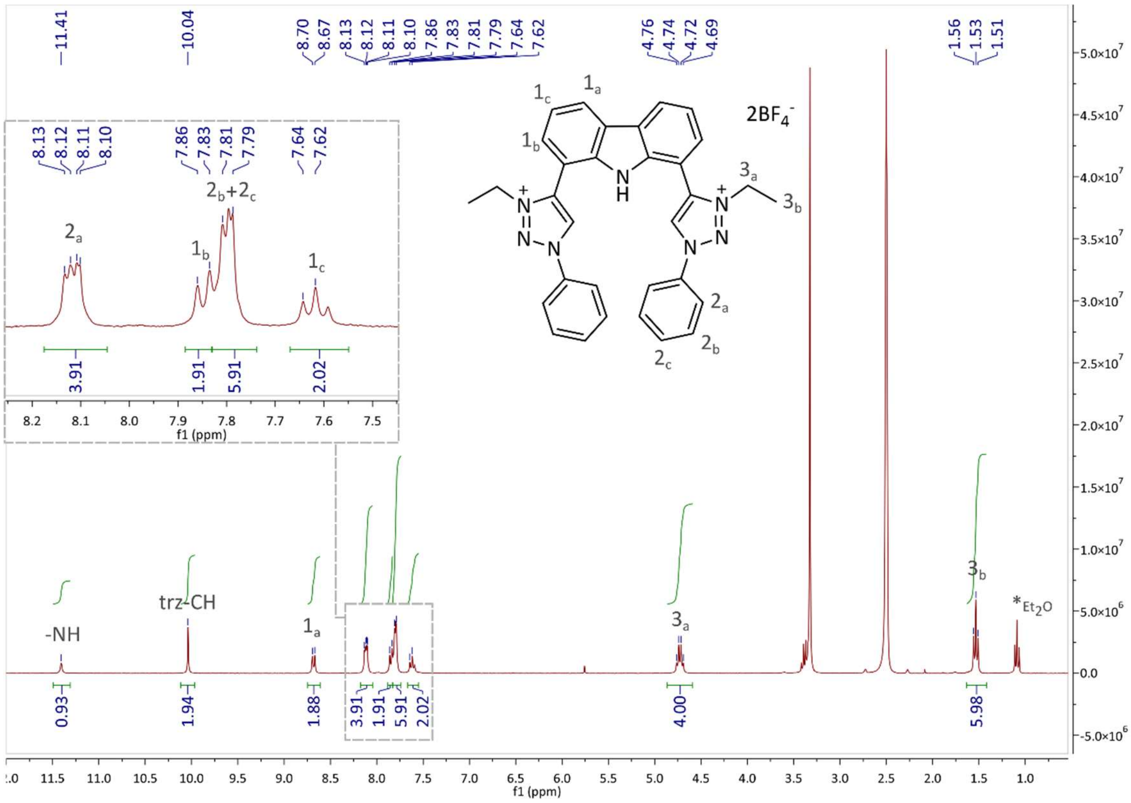

Figure S11. ^{1}H NMR spectrum of **L2** in $(CD_3)_2SO$.

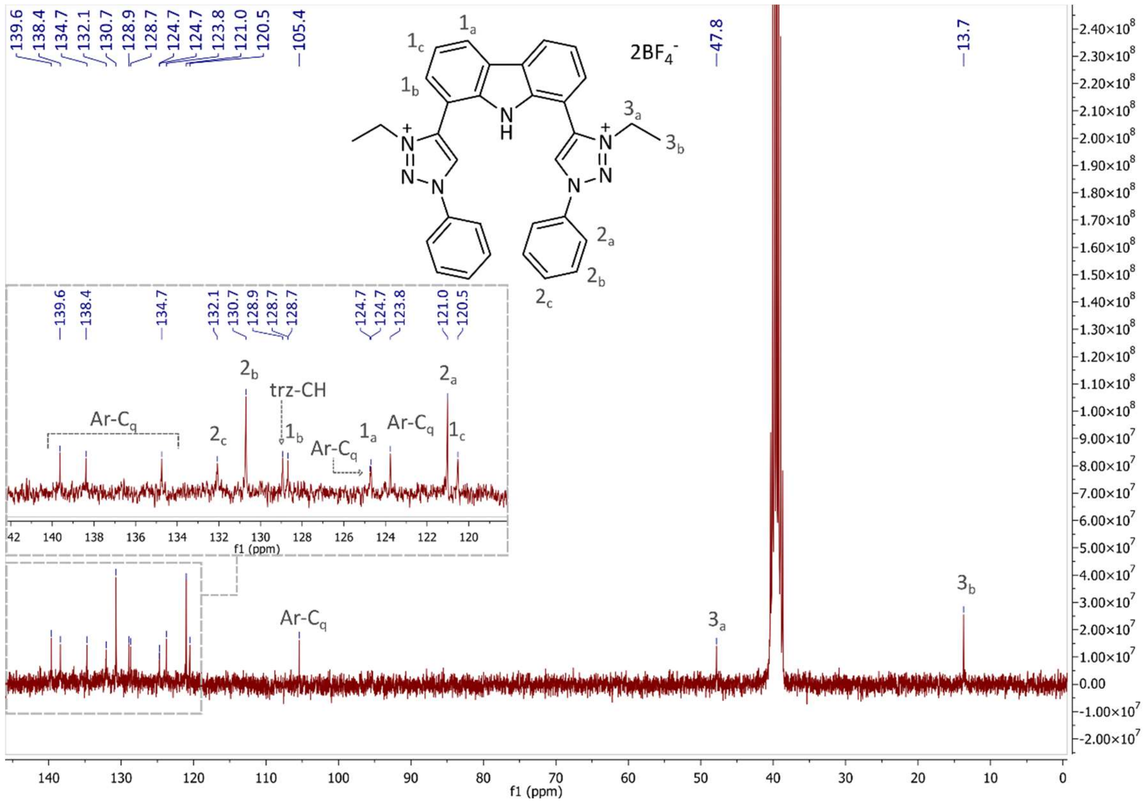

Figure S12. The ^{13}C {^{1}H} NMR spectrum of **L2** in $(CD_3)_2SO$.

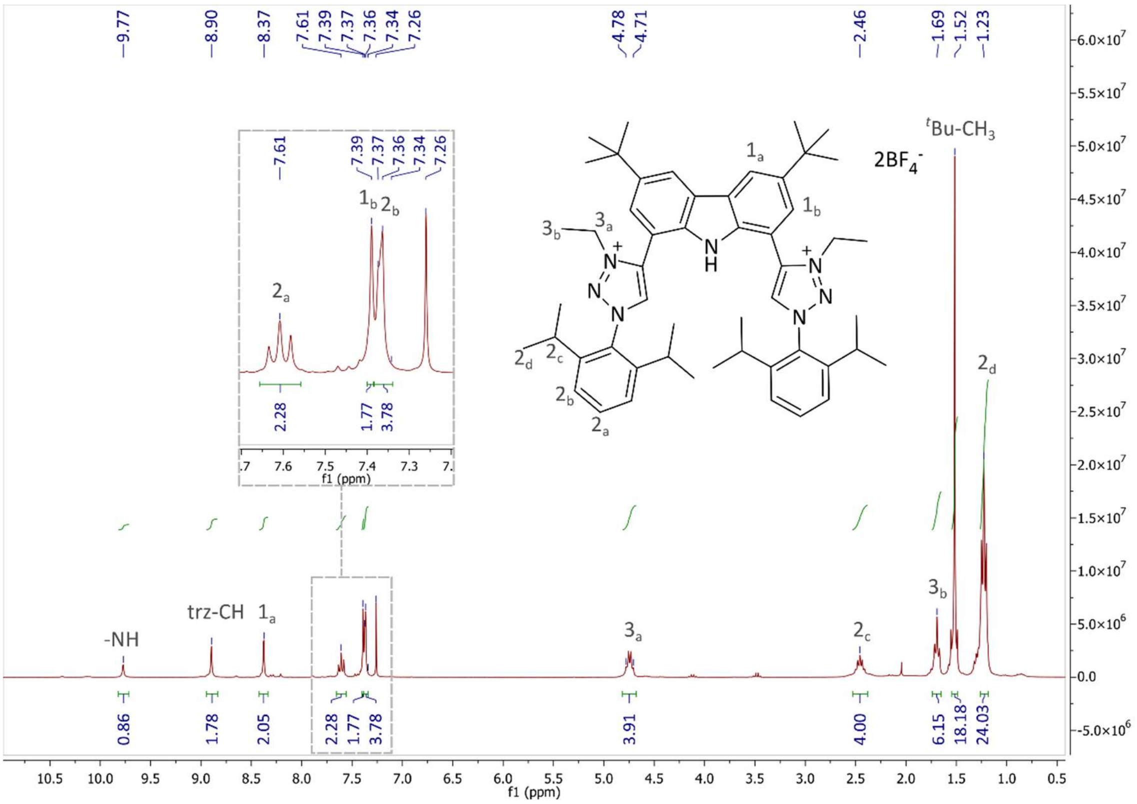

Figure S13. ^{1}H NMR spectrum of **L5** in $CDCl_3$.

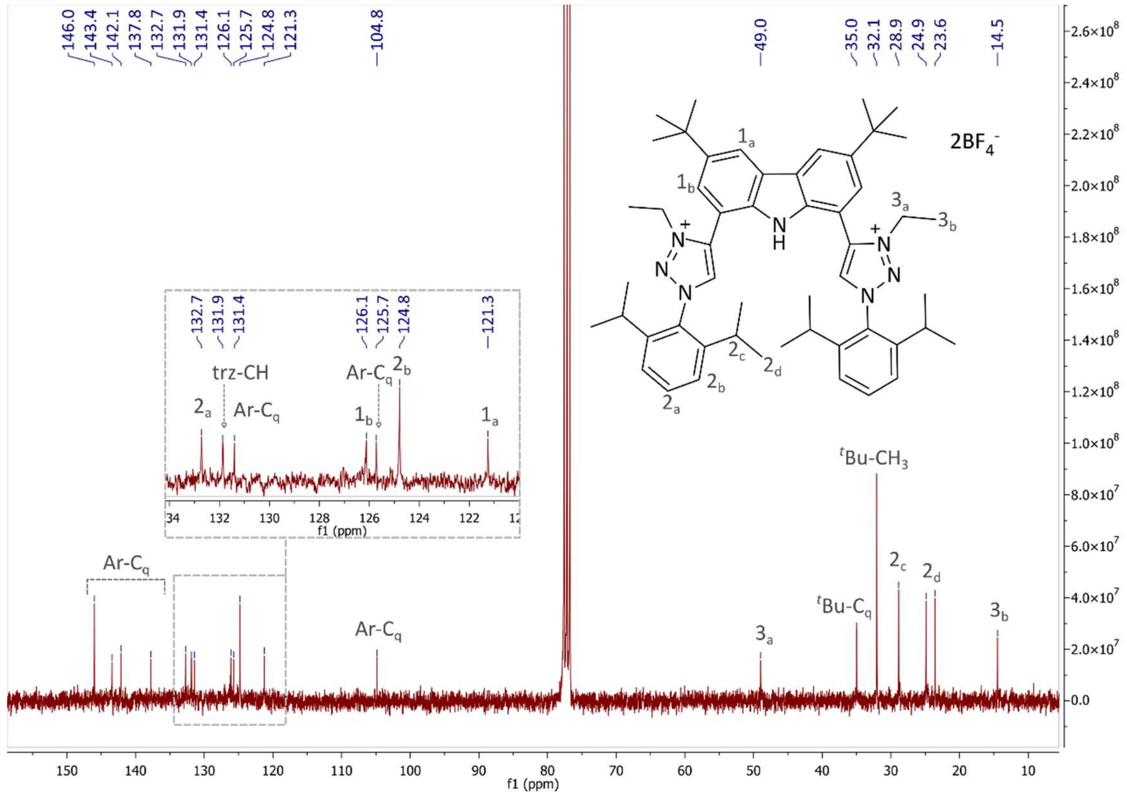

Figure S14. The ^{13}C {^{1}H} NMR spectrum of **L5** in $CDCl_3$.

Synthesis of the triazolium salt, *1,8-bis-(1,3-bis-(2,6-diisopropylphenyl)-1,2,3-triazolium)-9H-carbazole*, L4.

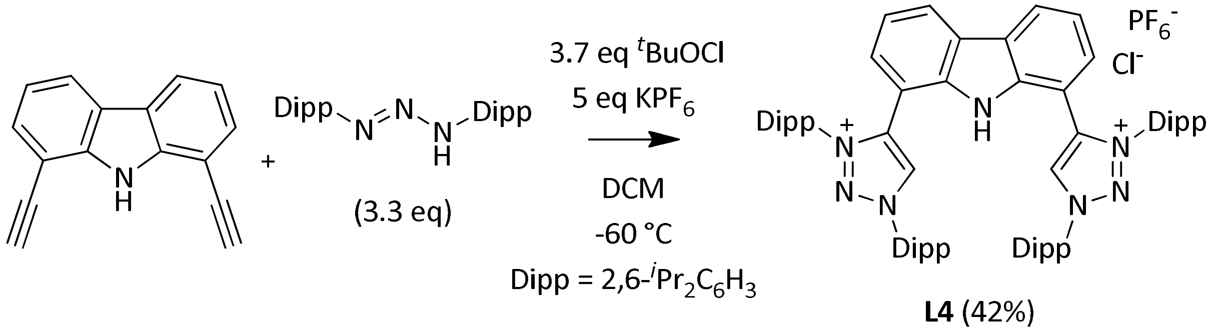

Scheme S7. Synthesis of 1,8-bis-(1,3-bis-(2,6-diisopropylphenyl)-1,2,3-triazolium)-9*H*-carbazole, L4.

An adapted procedure of the previously reported method for a diarylated triazolium salt synthesis was followed.[11,23] 1,8-diethynyl-9*H*-carbazole (0.9 g, 4.18 mmol, 1 eq), 1,3-bis(2,6-diisopropylphenyl)triaz-1-ene[5,6] (5.1 g, 13.96 mmol, 3.3 eq) and KPF_6 (3.85 g, 20.92 mmol, 5 eq) were dissolved in anhydrous CH_2Cl_2 and cooled to −60 °C. In the dark, ^{t}BuOCl (1.51 g, 15.36 mmol, 3.7 eq) was added dropwise and the reaction was left overnight to warm up to room temperature. The reaction mixture was filtered, and the dark red filtrate was evaporated under reduced pressure. The crude solid product was subsequently triturated with hexane and diethyl ether affording the triazolium salt as a white solid. Yield: 2.20 g (42 %). M.p. >300 °C. ^{1}H NMR (300 MHz, CD_3CN) δ 13.46 (s, 1H, NH), 10.82 (s, 2H, trz-CH), 8.33 (d, *J* = 7.7 Hz, 2H, Ar-$CH_{carbazole}$, H-1_a), 7.75 (t, *J* = 7.9 Hz, 2H, Ar-CH_{Dipp}, H-2_c), 7.73 (t, *J* = 7.9 Hz, 2H, Ar-CH_{Dipp}, H-2_c), 7.52 (d, *J* = 7.9 Hz, 4H, Ar-CH_{Dipp}, H-2_b), 7.45 (d, *J* = 7.8 Hz, 4H, Ar-CH_{Dipp}, H-2_b), 7.20 (dd, *J* = 7.8, 7.8 Hz, 2H, Ar-$CH_{carbazole}$, H-1_c), 7.04 (d, *J* = 7.8 Hz, 2H, Ar-$CH_{carbazole}$, H-1_b), 2.73 (sept, *J* = 6.7 Hz, 4H, ^{i}Pr-CH, H-2_c), 2.63 (sept, *J* = 6.6 Hz, 4H, ^{i}Pr-CH, H-2_c), 1.10 (d, *J* = 6.8 Hz, 24H), 0.99 (d, *J* = 6.7 Hz, 12H), 0.88 (d, *J* = 6.7 Hz, 12H) (all ^{i}Pr-CH_3). ^{13}C {^{1}H} NMR (75 MHz, CD_3CN) δ 146.7 (Ar-C_q), 146.6 (Ar-C_q), 134.3 (Ar-CH_{Dipp}, C-2_a), 134.1 (Ar-CH_{Dipp}, C-2_a), 131.8 (trz-CH), 130.9 (Ar-C_q), 128.0 (Ar-$CH_{carbazole}$, C-1_b), 127.4 (Ar-C_q), 126.7 (Ar-CH_{Dipp}, C-2_b), 126.1 (Ar-CH_{Dipp}, C-2_b), 125.5 (Ar-$CH_{carbazole}$, C-1_a), 121.6 (Ar-$CH_{carbazole}$, C-1_c), 108.2 (Ar-C_q), 29.9, 29.6 (all ^{i}Pr-CH, C-2_c), 25.4, 25.0, 23.8, 22.5 (all ^{i}Pr-CH_3, C-2_d). ^{19}F {^{1}H} NMR (470 MHz, CD_3CN) δ -67.63 (d, *J* = 707.7 Hz, PF_6). ^{31}P {^{1}H} NMR (202 MHz, CD_3CN) δ -139.9 (sept, *J* = 706.5 Hz, PF_6). ESI-(+)MS (Q-TOF) (positive mode, m/z): calcd for $[M{-}Cl{-}PF_6]^{2+}$: 471.8116. Found: 471.8117.

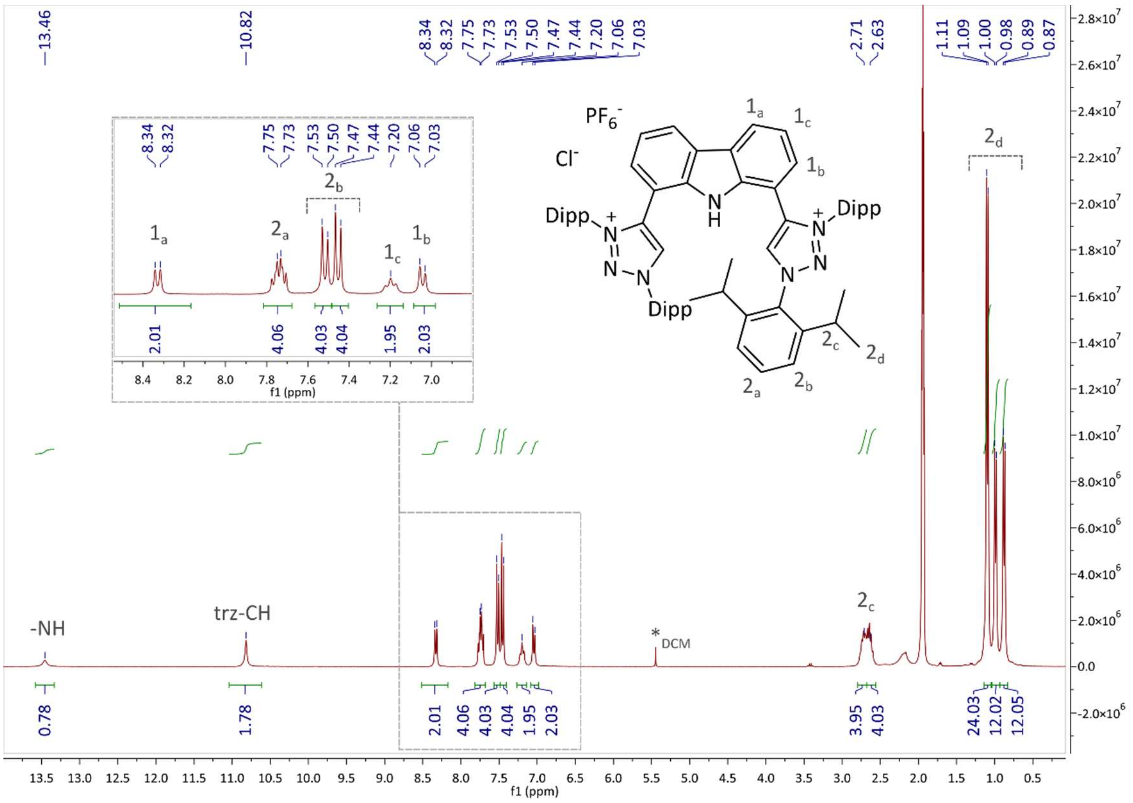

Figure S15. The ^{1}H NMR spectrum of **L4** in CD_3CN.

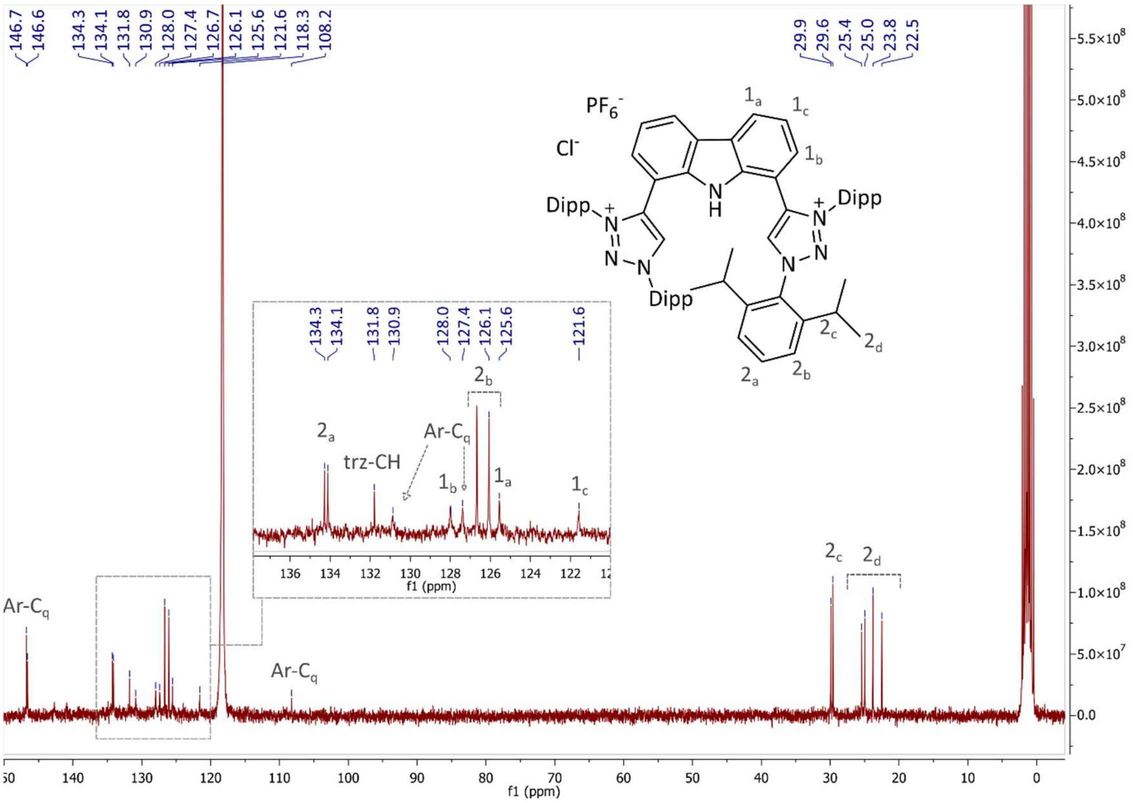

Figure S16. The ^{13}C {^{1}H} NMR spectrum of **L4** in CD_3CN.

1.4 Synthesis of metal complexes

Synthesis of *bis-((3,6-tert-butyl)-1,8-bis(1-ethyl-3-phenyl-1,2,3-triazol-5-ylidene gold(I)-9-silver(I) chlorido carbazolide),* 1b and *bis-((3,6-tert-butyl)-1,8-bis(1-ethyl-3-phenyl-1,2,3-triazo-5-lylidene gold(I)-9H-carbazolide dichloride-argentate(I),* 1c

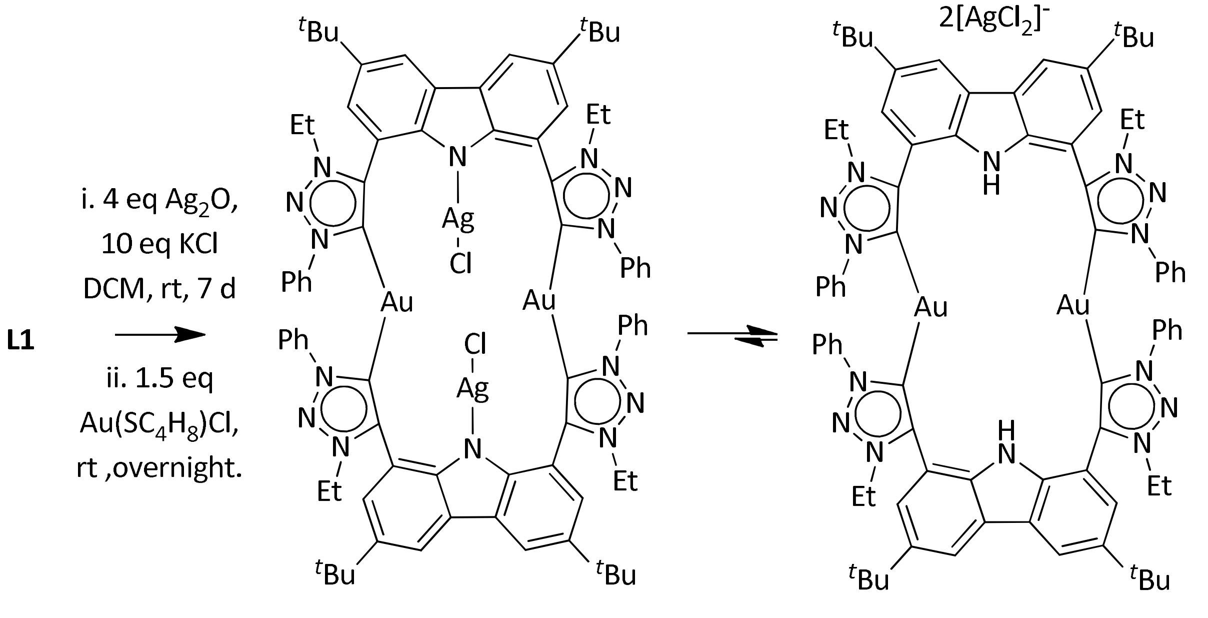

1b **1c** (28%)

Scheme S8. Synthesis of **1a and 1b**.

L1 (0.15 g, 0.20 mmol), 10 eq KCl (0.14 g, 1.9 mmol) and 4 eq Ag_2O (0.18 g, 0.75 mmol) were dissolved in anhydrous CH_2Cl_2 (10 mL). The reaction was stirred for one week in the dark at room temperature. The solvent was extracted via cannula filtration and 1.5 eq Au(tht)Cl (0.90 mg, 0.28 mmol) was added. An immediate precipitate was observed, and the reaction was stirred overnight. The solvent was evaporated, and the crude was extracted with CH_2Cl_2, followed by washing with hexane. Crystals suitable for XRD analysis were grown from a concentrated CH_2Cl_2 solution, layered with pentane. Yellow powder. Yield: 96 mg (28%). ^1H NMR (500 MHz, $CDCl_3$) δ 8.24 (d, J = 2.0 Hz, 2H, Ar-$CH_{carbazole}$, H-1_a), 8.19 (d, J = 2.0 Hz, 2H, Ar-$CH_{carbazole}$, H-1_a'), 7.66 (d, J = 8.3 Hz, 2H, Ar-CH_{Ph}, H-2_a), 7.66' (d, J = 8.7 Hz, 2H, Ar-CH_{Ph}, H-2_a), 7.62 (d, J = 7.3 Hz, 2H, Ar-CH_{Ph}, H-2_a), 7.62' (d, J = 8.0 Hz, 2H, Ar-CH_{Ph}, H-2_a), , 7.70 (dd, J = 7.6, 7.6 Hz, 4H, Ar-CH_{Ph}, H-2_b), 7.35 (t, J = 7.3 Hz, 2H, Ar-CH_{Ph}, H-2_c), 7.18 (t, J = 7.5 Hz, 2H, Ar-CH_{Ph}, H-2_c'), 7.09 (d, J = 2.0 Hz, 2H, Ar-$CH_{carbazole}$, H-1_b), 7.07 (m, 4H, Ar-CH_{Ph}, H-2_b'), 7.05 (d, J = 1.9 Hz, 2H, Ar-$CH_{carbazole}$, 2H, H-1_b'), 4.22 (dq, J = 14.0, 7.1 Hz, 2H, Et-CH_2, H-3_a), 4.16 (dq, J = 14.1, 7.1 Hz, 2H, Et-CH_2, H-3_a), 3.55 (dq, J = 14.1, 7.1 Hz, 2H, Et-CH_2, H-3_a'), 3.13 (dq, J = 14.1, 7.1 Hz, 2H, Et-CH_2, H-3_a'), 1.51 (s, 18H, ^{t}Bu-CH_3,), 1.41 (s, 18H, ^{t}Bu-CH_3'), 1.36 (t, J = 7.3 Hz, 6H, Et-CH_3, H-3_b), 0.90 (t, J = 7.3 Hz, 6H, Et-CH_3, H-3_b'). ^{13}C {^1H} NMR (125 MHz, $CDCl_3$) δ 157.08 (Au-$C_{carbene}$), 157.06 (Au-$C_{carbene'}$), 148.6, 148.5, 148.3(3), 148.3, 148.0, 147.9, 146.4, 139.1, 138.4, 137.8, 137.4 (all Ar-C_q), 129.8 (Ar-CH_{Ph}, C-2_b), 129.6 (Ar-CH_{Ph}, C-2_c), 129.4 (Ar-CH_{Ph}, C-2_c), 128.8 (Ar-CH_{Ph}, C-2_b), 126.6, 126.5, 125.7, 125.6 (all Ar-C_q), 124.4 (Ar-$CH_{carbazole}$, C-1_b), 123.6 (Ar-$CH_{carbazole}$, C-1_b), 123.2 (Ar-CH_{Ph}, C-2_a), 123.1 (Ar-CH_{Ph}, C-2_a), 120.4 (Ar-$CH_{carbazole}$, C-1_b), 118.4

(Ar-$CH_{carbazole}$, C-1_b), 110.8 (Ar-C_q), 110.3 (Ar-C_q), 46.4 (Et-CH_2, C-3_a), 45.1 (Et-CH_2, C-3_a), 34.7 (^{t}Bu-C_q), 34.6 (^{t}Bu-C_q), 32.6 (^{t}Bu-CH_3), 32.3 (^{t}Bu-CH_3), 16.2 (Et-CH_3, C-3_b), 15.4 (Et-CH_3, C-3_b). Anal. Calcd for $C_{80}H_{86}Au_2N_{14}Ag_2Cl_4$ (+ 1.5 eq acetone): C 48.74, H 4.60, N 9.42. Found (avg): C 48.52, H 4.51, N 9.04. ESI-(+)MS (Q-TOF) (positive mode, m/z): calcd for $[M+2H-2AgCl_2]^{2+}$: 818.3240. Found: 818.3253.

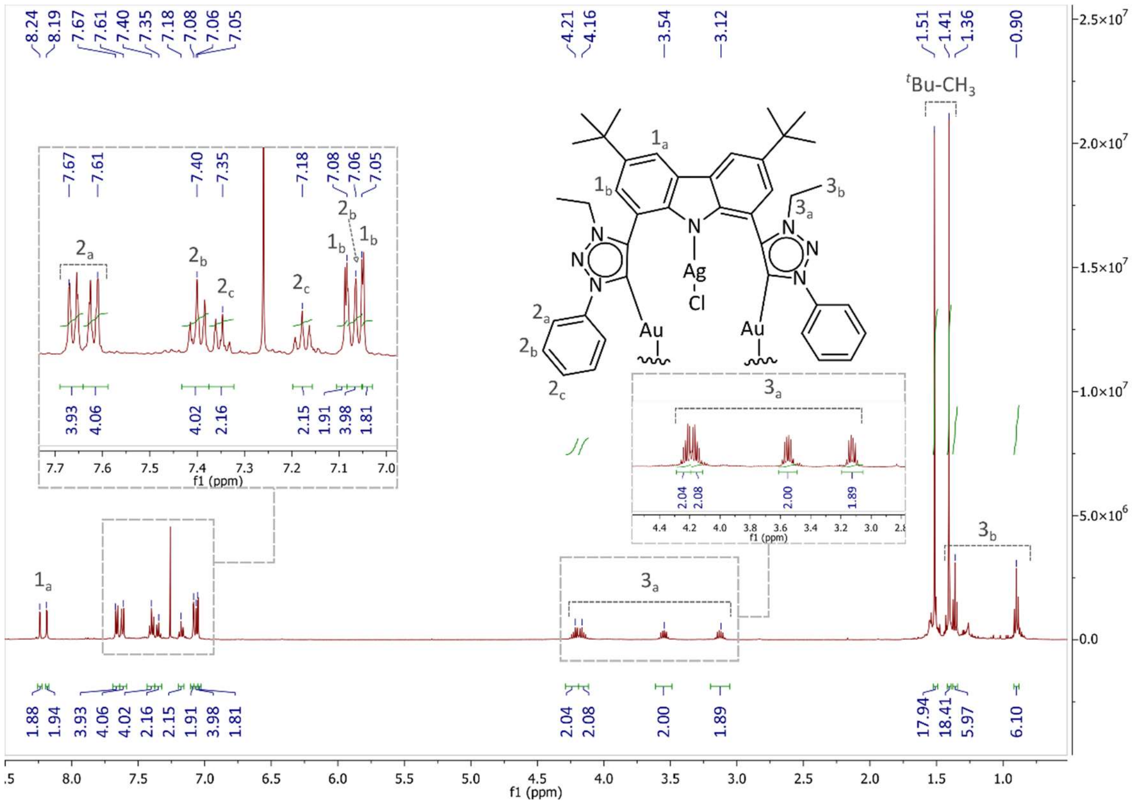

Figure S17. ^{1}H NMR spectrum of **1b** in $CDCl_3$.

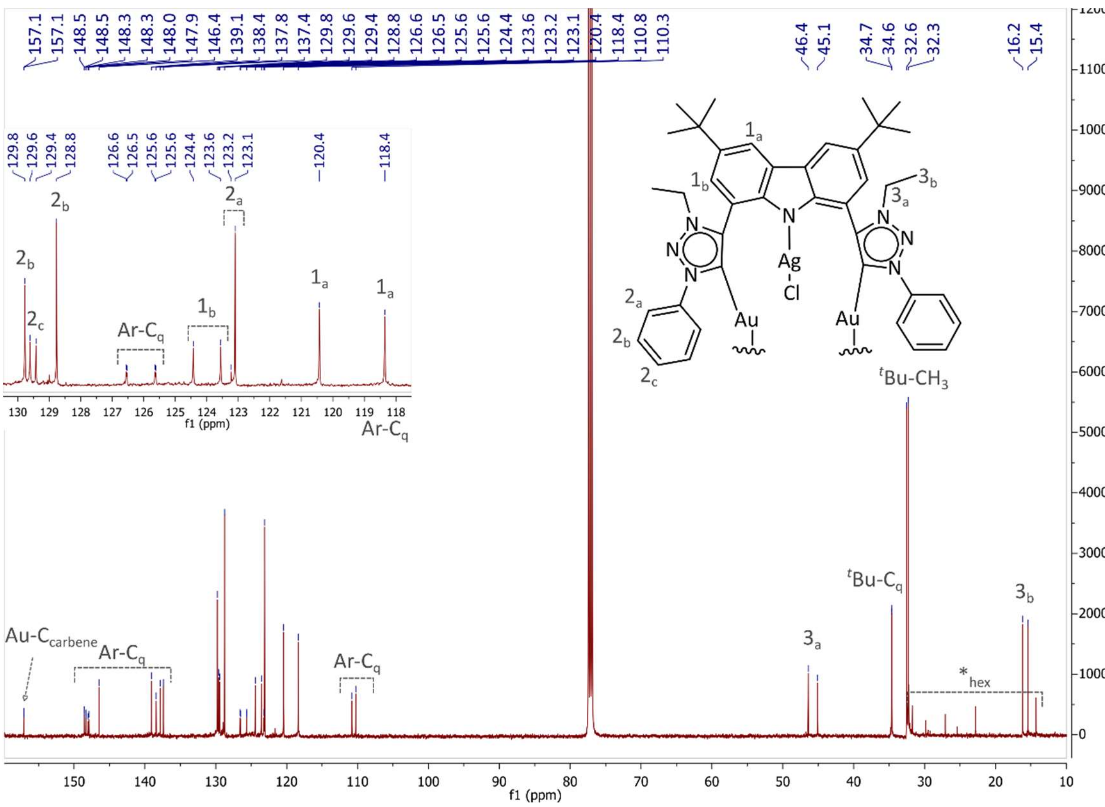

Figure S18. The ^{13}C {^1H} NMR spectrum of **1b** in $CDCl_3$.

Synthesis and characterization of *bis-((3,6-tert-butyl)-1-(1-ethyl-3-phenyl-1,2,3-triazo-5-lylidene silver(I) chloride)-8-(1-ethyl-3-phenyl-1-2,3-triazol-5-ylidene silver(I))-9-carbazolide)*, 1a .

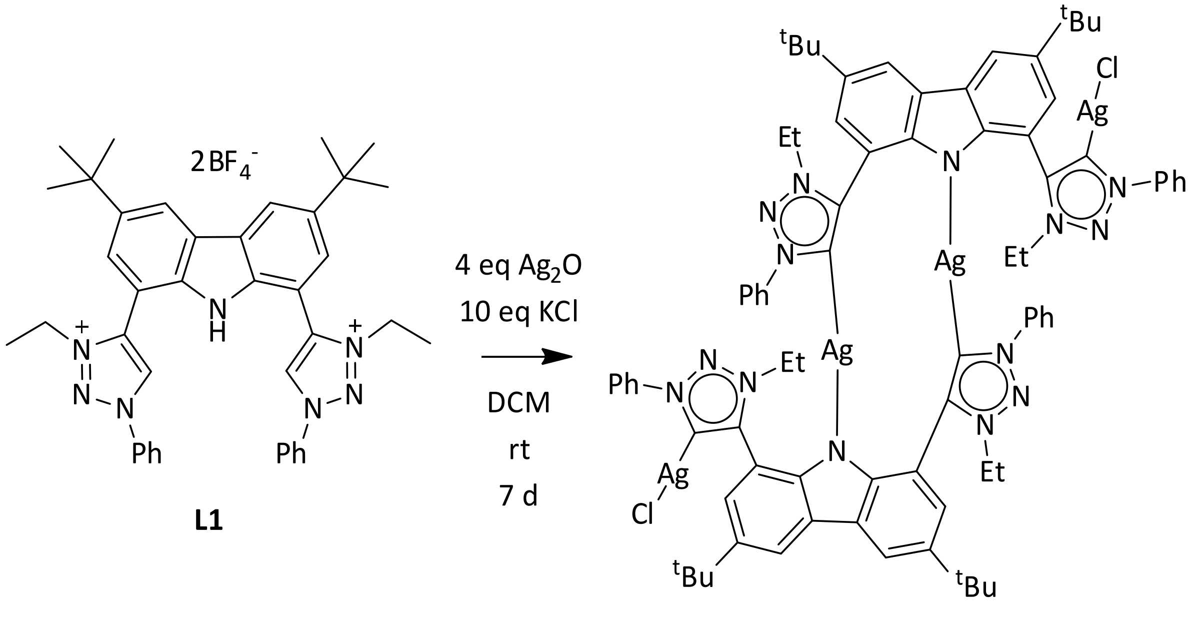

1c (14%)

Scheme S9. Synthesis of **1a**.

To a flame dried Schlenk was added **L1** (100 mg, 0.13 mmol), KCl (90 mg, 1.3 mmol) and Ag_2O (11 mg, 0.50 mmol) and dissolved in anhydrous CH_2Cl_2 (10 mL). The reaction was stirred for one week in the dark at room temperature. The solvent was evaporated, and the crude product was extracted with CH_2Cl_2 and washed with Et_2O, affording **1a** as a bright yellow powder. Crystals suitable for XRD analysis were grown from slow evaporation of diethyl ether in a concentrated CH_2Cl_2 solution. Yield: 30 mg (14%). ^{1}H NMR (300 MHz, $CDCl_3$) δ 8.23 (d, *J* = 2.0 Hz, 2H, Ar-$CH_{carbazole}$, H-1$_a$), 8.17 (d, *J* = 2.0 Hz, 2H, Ar-$CH_{carbazole}$, H-1$_a$′), 7.49 (d, *J* = 7.9 Hz, 4H, Ar-CH_{Ph}, H-2$_b$), 7.40 (d, *J* = 4.2 Hz, 8H, Ar-CH_{Ph}, H-2$_a$), 7.33–7.30 (m, 2H, Ar-CH_{Ph}, H-2$_c$), 7.16–7.12 (m, 2H, Ar-CH_{Ph}, H-2$_c$′), 7.11 (d, *J* = 2.0 Hz, 2H, Ar-$CH_{carbazole}$, H-1$_b$), 7.08 (d, *J* = 2.0 Hz, 2H, Ar-$CH_{carbazole}$, H-1$_b$′) 7.04 (dd, *J* = 7.8, 7.8 Hz, 4H, Ar-CH_{Ph}, H-2$_b$′), 4.37 (dq, *J* = 14.4, 7.3 Hz, 2H, Et-CH_2, H-3$_a$), 4.19 (dq, *J* = 14.4, 7.2 Hz, 2H, Et-CH_2, H-3$_a$), 3.64 (dt, *J* = 14.4, 7.3 Hz, 2H, Et-CH_2, H-3$_a$′), 3.25 (dq, *J* = 14.5, 7.3 Hz, 2H, Et-CH_2, H-3$_a$′), 1.53 (s, 18H, ^{t}Bu-CH_3), 1.47 (t, *J* = 7.3 Hz, 6H, Et-CH_3, H-3$_b$), 1.41 (s, 18H, ^{t}Bu-CH_3′), 0.91 (t, *J* = 7.3 Hz, Et-CH_3, H-3$_b$′). ^{13}C {^{1}H} NMR (100 MHz, $CDCl_3$) δ n.o. (Ag-$C_{carbene}$,), 148.7, 148.6, 148.5, 148.4, 148.1, 147.9, 147.7, 147.6, 139.7, 138.4, 138.0, 137.4, 130.0 (all Ar-C_q), 129.9 (Ar-CH_{Ph}, C-2$_a$),129.5 (Ar-CH_{Ph}, C-2$_c$), 129.4 (Ar-CH_{Ph}, C-2$_c$),129.0 (Ar-CH_{Ph}, C-2$_b$), 126.8, 126.7, 125.8, 125.7 (all Ar-C_q), 124.5 (Ar-$CH_{carbazole}$, C-1$_b$), 123.3 (Ar-$CH_{carbazole}$, C-1$_b$′), 122.1 (Ar-CH_{Ph}, C-2$_b$′), 120.9 (Ar-C_q), 120.1 (Ar-CH_{Ph}, C-2$_a$′), 118.5 (Ar-$CH_{carbazole}$, C-1$_a$), 118.2 (Ar-$CH_{carbazole}$, C-1$_a$′), 111.1 (Ar-C_q), 110.9 (Ar-C_q), 46.6 (Et-CH_2, C-3$_a$), 44.7 (Et-CH_2, C-3$_a$′), 34.7 (^{t}Bu-C_q), 34.6 (^{t}Bu-C_q′), 32.6 (^{t}Bu-CH_3), 32.3 (^{t}Bu-CH_3′), 16.2 (Et-CH_3, C-3$_b$), 15.5 (Et-CH_3, C-3$_b$′). Anal. Calcd for $C_{80}H_{84}N_{14}Ag_4Cl_2$ (+ 1.0 eq tetrahydrothiophene): C 55.19, H 4.85, N 10.73 S 1.75. Found: C 55.67, H 4.51, N 11.05 S 1.78.

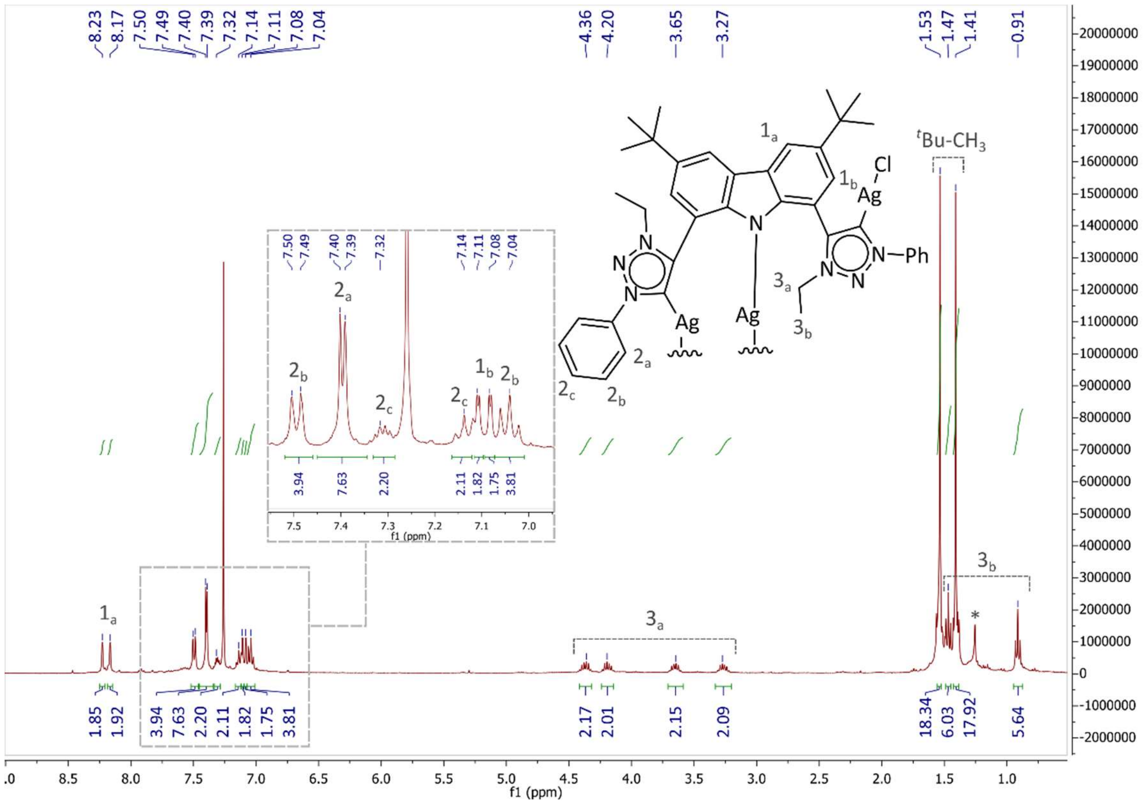

Figure S19. ^{1}H NMR spectrum of **1a** in $CDCl_3$ (*grease).

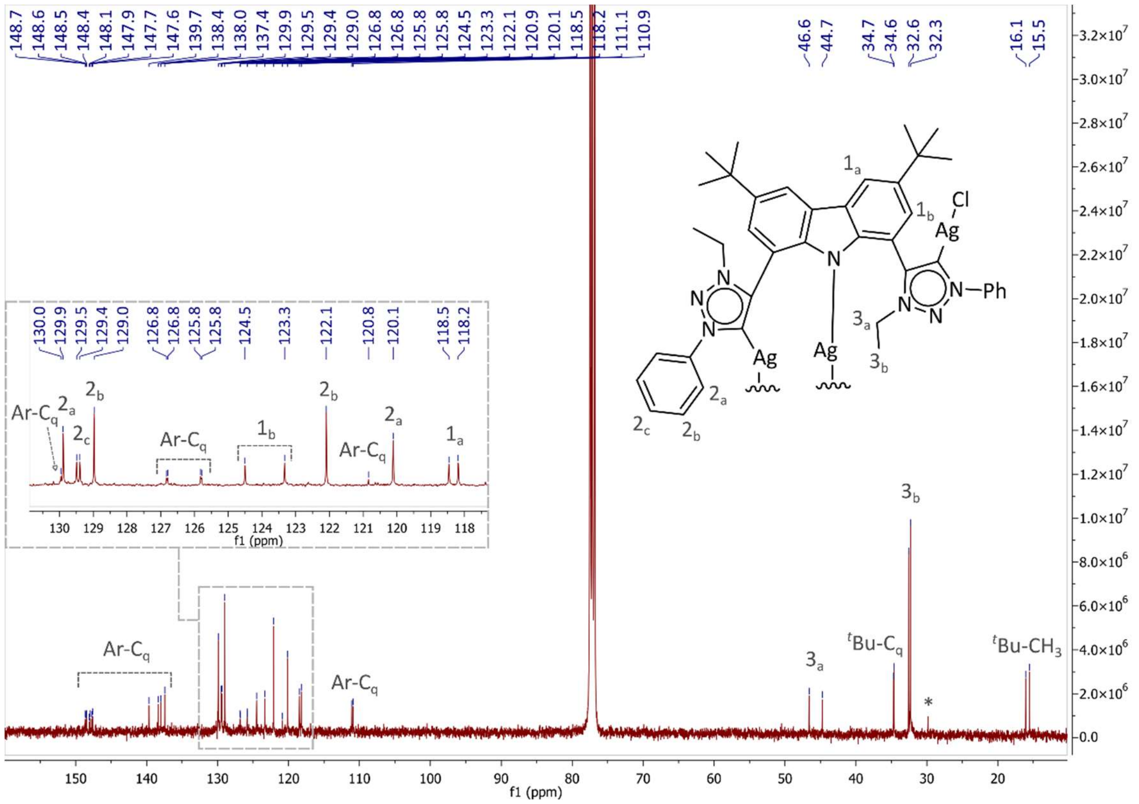

Figure S20. The ^{13}C {^{1}H} NMR spectrum of **1a** in $CDCl_3$ (*grease).

Synthesis of 2

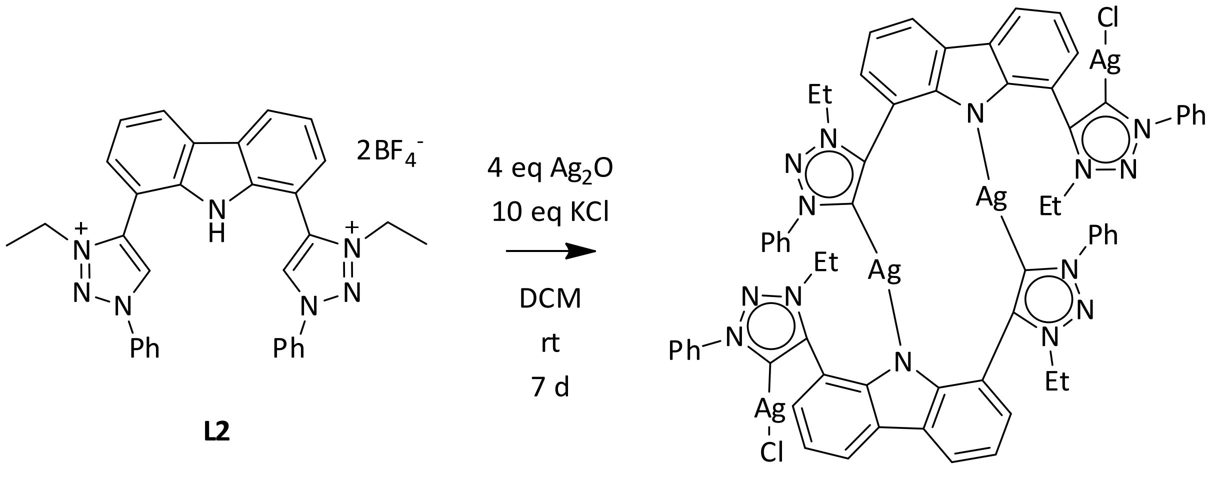

2 (27%)

Scheme S10. Synthesis of **2**.

To a flame dried Schlenk was added **L2** (50 mg, 0.073 mmol), 10 eq KCl (55 mg, 0.73 mmol) and 4 eq Ag_2O (68 mg, 0.29 mmol) and dissolved in anhydrous CH_2Cl_2 (10 mL). The reaction was stirred for one week in the dark at room temperature. The solvent was evaporated, and the crude product was extracted with CH_2Cl_2 and washed with Et_2O, affording a light orange powder. Yield: 30 mg (27 %). ^{1}H NMR (500 MHz, $CDCl_3$) δ 8.22 (d, J =7.6 Hz, 1H, Ar-CH$_{carbazole}$, H-1$_a$), 8.22′ (d, J =7.6 Hz, 1H, Ar-CH$_{carbazole}$, H-1$_a$), 8.19 – 8.16 (m, 2H, Ar-CH$_{carbazole}$, H-1$_a$′), 7.46 – 7.44 (m, 4H, Ar-CH$_{Ph}$, H-2$_a$), 7.39 – 7.35 (m, 8H, Ar-CH$_{Ph}$, H-2$_b$), 7.30 –7.28 (m, 2H, Ar-CH$_{Ph}$,

H-2$_c$), 7.17 (dd, *J* = 7.4, 7.4 Hz, Ar-CH$_{carbazole}$, H-1$_c$), 7.12 – 7.10 (m, 4H, overlapping Ar-CH$_{carbazole}$, H-1$_c$′ (2H) + Ar-CH$_{Ph}$, H-2$_c$′(2H)), 7.06 – 7.04 (m, 4H, Ar-CH$_{carbazole}$, H-1$_b$), 7.04 – 7.00 (m, 4H, Ar-CH$_{Ph}$, H-2$_a$′), 4.41 (dq, *J* = 14.5, 7.2 Hz, 2H, Et-CH$_2$, H-3$_a$), 4.18 (dq, *J* = 14.5, 7.3 Hz, 2H, Et-CH$_2$, H-3$_a$), 3.77 (dq, *J* = 13.5, 7.4 Hz, 2H, Et-CH$_2$, H-3$_a$′), 3.26 (dq, *J* = 14.4, 7.3 Hz, 2H, Et-CH$_2$, H-3$_a$′), 1.48 (t, *J* = 7.3 Hz, 6H, Et-CH$_3$, H-3$_b$), 0.92 (t, *J* = 7.4 Hz, 6H, Et-CH$_3$, H-3$_b$′). ^{13}C {^{1}H} NMR (125 MHz, CDCl$_3$) δ 164.5 (dd, *J* = 239.9, 17.5 Hz, Ag-C$_{carbene}$), 164.4 (dd, *J* = 240.6, 16.4 Hz, Ag-C$_{carbene}$), 149.9(3), 149.9(1), 149.8(5), 149.8(2), 147.5, 147.4, 147.1, 147.0, 139.5, 139.1, 130.2 (all Ar-C$_q$), 129.9 (Ar-CH$_{Ph}$, C-2$_b$), 129.6 (Ar-CH$_{Ph}$, C-2$_c$), 129.4 (Ar-CH$_{Ph}$, C-2$_c$), 129.0 (Ar-CH$_{Ph}$, C-2$_a$), 126.9 (Ar-CH$_{carbazole}$, C-1$_b$), 126.0(3) (Ar-C$_q$), 126.0 (Ar-C$_q$), 125.5 (Ar-CH$_{carbazole}$, C-1$_b$), 122.4 (Ar-CH$_{carbazole}$, C-1$_a$), 122.1(3) (Ar-CH$_{Ph}$, C-2$_a$), 122.1 (Ar-CH$_{carbazole}$, C-1$_a$), 119.9 (Ar-CH$_{Ph}$, C-2$_b$), 115.6 (Ar-CH$_{carbazole}$, C-1$_a$), 115.0 (Ar-CH$_{carbazole}$, C-1$_a$), 111.9 (Ar-C$_q$), 111.7 (Ar-C$_q$), 46.8 (Et-CH$_2$, C-3$_a$), 45.0 (Et-CH$_2$, C-3$_a$), 15.9 (Et-CH$_3$, C-3$_b$), 14.7 (Et-CH$_3$, C-3$_b$). ESI-(+)MS (Q-TOF) (positive mode, m/z): calcd for $[M+2H]^{2+}$: 617.1451. Found: 617.1380, where M = $C_{64}H_{54}Ag_2N_{14}$.

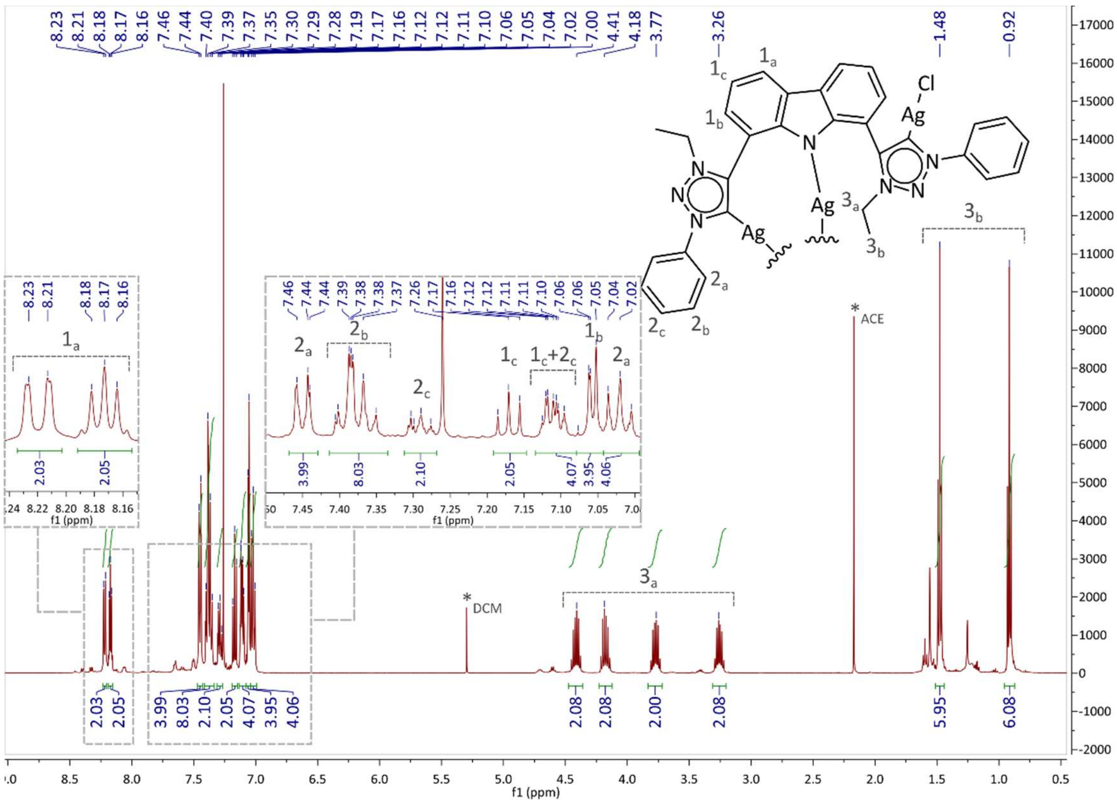

Figure S21. ^{1}H NMR spectrum of **2** in CDCl$_3$.

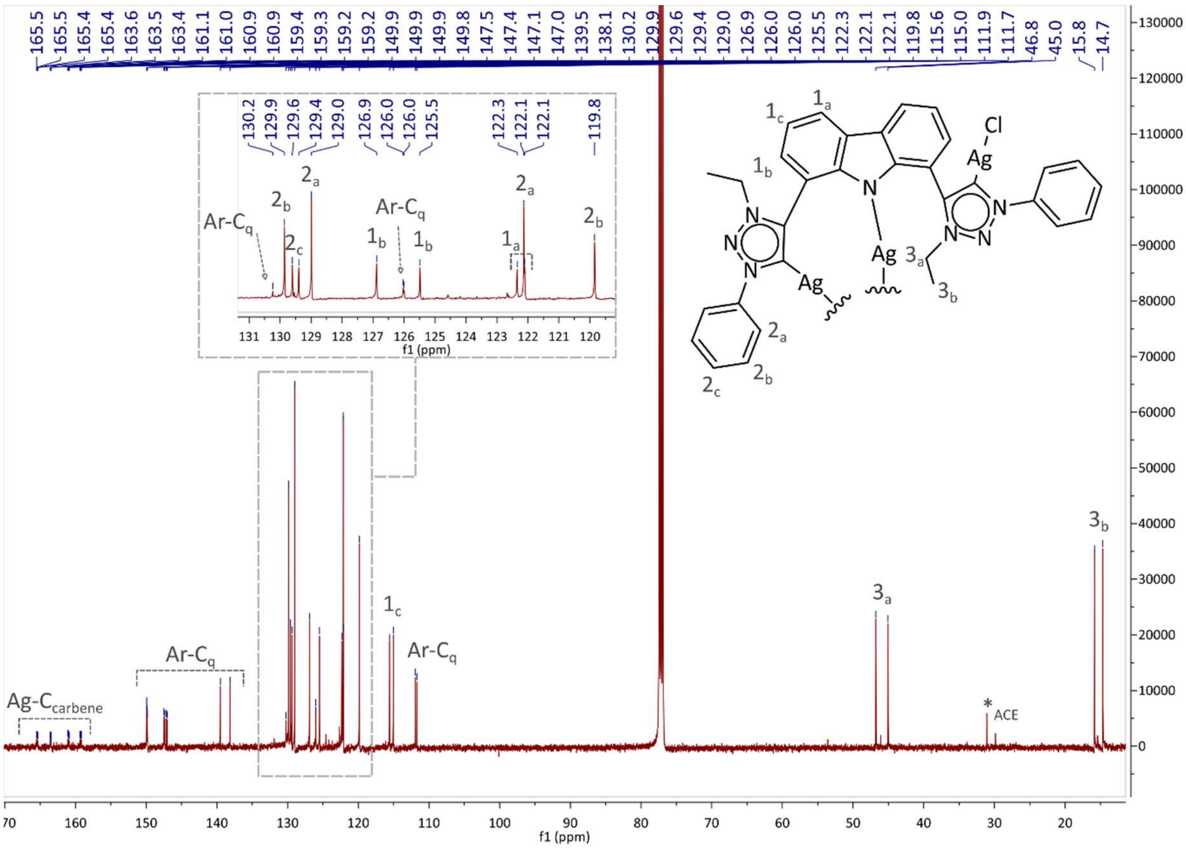

Figure S22. The ^{13}C {^{1}H} NMR spectrum of **2** in $CDCl_3$

Synthesis of the *gold(I) pincer complex, 1,8-(bis(1,3-bis(2,6-diisopropylphenyl)-1,2,3-triazol-5-ylidene)carbazolide-C^N^C-gold(I)*, 4a.

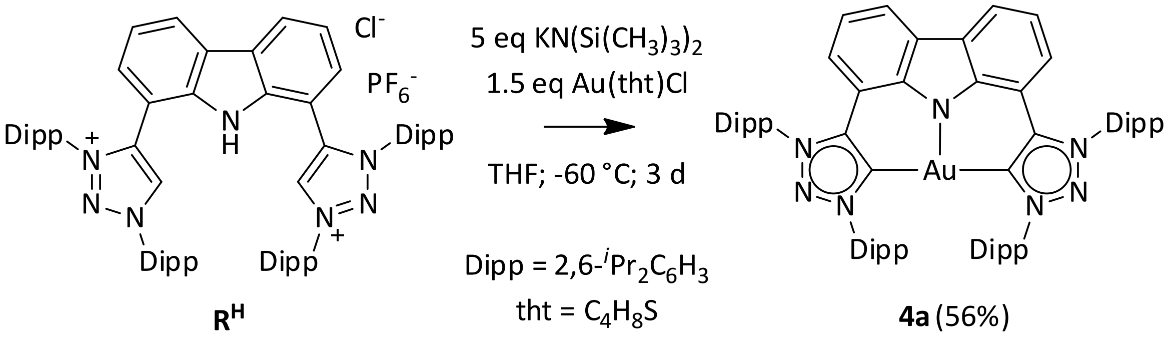

Scheme S11. Synthesis of **4a**.

The ligand precursor salt **L4** (0.30 g, 0.24 mmol, 1 eq), $KN(Si(CH_3)_3)_2$ (0.24 g, 1.2 mmol, 5 eq) and Au(tht)Cl (0.17 g, 0.36 mmol, 1.5 eq) were weighed off into a flame-dried Schlenk flask inside the glovebox. Once removed from the glovebox and in the dark, the solids were dissolved in anhydrous THF at −60 °C and the dark red reaction mixture was left to slowly warm up to room temperature and continued for two more days. After the solvent was evaporated, the crude product was extracted with CH_2Cl_2 and dried. The crude product was subsequently resuspended and stirred overnight in diethyl ether. The solvent was evaporated, the product was washed with hexane and dried in vacuo, affording **4a** as a red solid. Crystals suitable for XRD analysis and in vitro cell culture studies were grown from a

concentrated solution of CH_2Cl_2, layered with pentane at −20 °C. Yield: 0.16 g (56 %). M.p. >300 °C. ^{1}H NMR (400 MHz, CD_2Cl_2) δ 8.13 (d, *J* = 7.4 Hz, 2H, Ar-$CH_{carbazole}$, H-1_a), 7.71 (d, *J* = 7.8 Hz, 2H, Ar-CH_{Dipp}, H-2_a), 7.51–7.47 (m, 6H, Ar-CH_{Dipp}, H-2_a (2H) + H-2_b (4H)), 7.15 (d, *J* = 7.9 Hz, 4H, Ar-CH_{Dipp}, H-2_b), 6.95 (d, *J* = 8.0 Hz, 2H, Ar-$CH_{carbazole}$, H-1_b), 6.65 (t, *J* = 7.7 Hz, 2H, Ar-$CH_{carbazole}$, H-1_c), 2.59 (sept, *J* = 6.8 Hz, 4H, ^{i}Pr-CH, H-2_c), 2.51 (sept, *J* = 6.9 Hz, 4H, ^{i}Pr-CH, H-2_c), 1.13 (d, *J* = 6.8 Hz, 12H, ^{i}Pr-CH_3, H-2_d), 1.07 (d, *J* = 7.2 Hz, 12H, ^{i}Pr-CH_3, H-2_d), 1.06 (d, *J* = 6.6 Hz, 12H, ^{i}Pr-CH_3, H-2_d), 0.98 (d, *J* = 6.8 Hz, 12H, ^{i}Pr-CH_3, H-2_d). ^{13}C {^{1}H} NMR (100 MHz, CD_2Cl_2) δ 175.0 (Au-$C_{carbene}$), 146.7, 146.6, 146.0, 145.3, 137.7, 135.4 (all Ar-C_q), 132.2 (Ar-CH_{Dipp}, C-2_a), 130.5 (Ar-CH_{Dipp}, C-2_a), 127.1 (Ar-C_q), 125.8 (Ar-CH_{Dipp}, C-2_b), 124.1 (Ar-CH_{Dipp}, C-2_b), 122.7 (Ar-$CH_{carbazole}$, C-1_b), 122.0 (Ar-$CH_{carbazole}$, C-1_a), 113.7 (Ar-$CH_{carbazole}$, C-1_c), 112.2 (Ar-C_q), 29.5, 29.2 (^{i}Pr-CH, C-2_c), 25.5, 25.0, 24.2, 23.4 (all ^{i}Pr-CH_3, C-2_d). ^{1}H NMR (400 MHz, C_6D_6) δ 8.33 (d, *J* = 7.8 Hz, 2H, Ar-$CH_{carbazole,}$ H-1_a), 7.36 (t, *J* = 7.8 Hz, 2H, Ar-CH_{Dipp}, H-2_a), 7.27 (m, 4H, (Ar-CH_{Dipp}, H-2_a (2H) + (Ar-$CH_{carbazole}$, H-1_b (2H)), 7.12 (d, *J* = 7.8 Hz, 4H, Ar-CH_{Dipp}, H-2_b), 7.10 (d, *J* = 7.8 Hz, 4H, Ar-CH_{Dipp}, H-2_b), 6.87 (dd, *J* = 7.7, 7.7 Hz, 2H, Ar-$CH_{carbazole}$, H-1_c), 2.94 (sept, *J* = 7.0 Hz, 4H, ^{i}Pr-CH, H-2_c), 2.68 (sept, *J* = 6.8 Hz, 4H, ^{i}Pr-CH, H-2_c), 1.15 (d, *J* = 6.7 Hz, 12H, ^{i}Pr-CH_3, H-2_d) 1.16 (d, *J* = 6.6 Hz, 12H, ^{i}Pr-CH_3, H-2_d), 1.05 (d, *J* = 6.8 Hz, 12H, ^{i}Pr-CH_3, H-2_d), 0.83 (d, *J* = 6.8 Hz, 12H, ^{i}Pr-CH_3, H-2_d). ^{13}C {^{1}H} NMR (100 MHz, C_6D_6) δ 175.9 (Au-$C_{carbene}$), 147.7, 147.3, 145.9, 145.3, 138.1, 135.6 (all Ar-C_q), 131.7 (Ar-CH_{Dipp}, C-2_a), 130.0 (Ar-CH_{Dipp}, C-2_a), 125.3 (Ar-CH_{Dipp}, C-2_b), 123.8 (Ar-CH_{Dipp}, C-2_b), 122.6 (Ar-$CH_{carbazole}$, C-1_b), 122.5 (Ar-$CH_{carbazole}$, C-1_a), 113.5 (Ar-$CH_{carbazole}$, C-1_c), 112.1 (Ar-C_q), 29.3, 29.1 (^{i}Pr-CH, C-2_c), 25.2, 24.7, 24.2, 23.0 (all ^{i}Pr-CH_3, C-2_d). Anal. Calcd for $C_{64}H_{74}N_7Au$ (+ 1.5 eq CH_2Cl_2): C 62.16, H 6.13, N 7.75. Found: C 62.22, H 6.37, N 7.35. ESI-(+)MS (Q-TOF) (positive mode, m/z): calcd for $[M+H]^+$: 1138.5743. Found: 1138.5786.

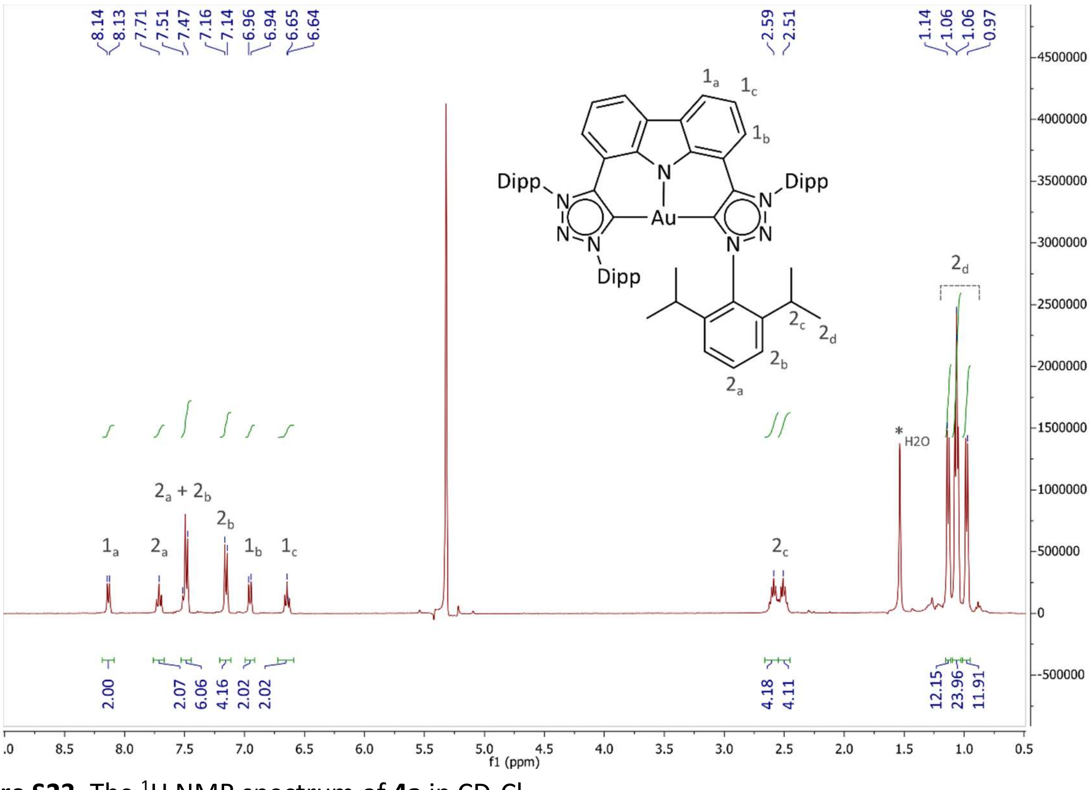

Figure S23. The ^{1}H NMR spectrum of **4a** in CD_2Cl_2.

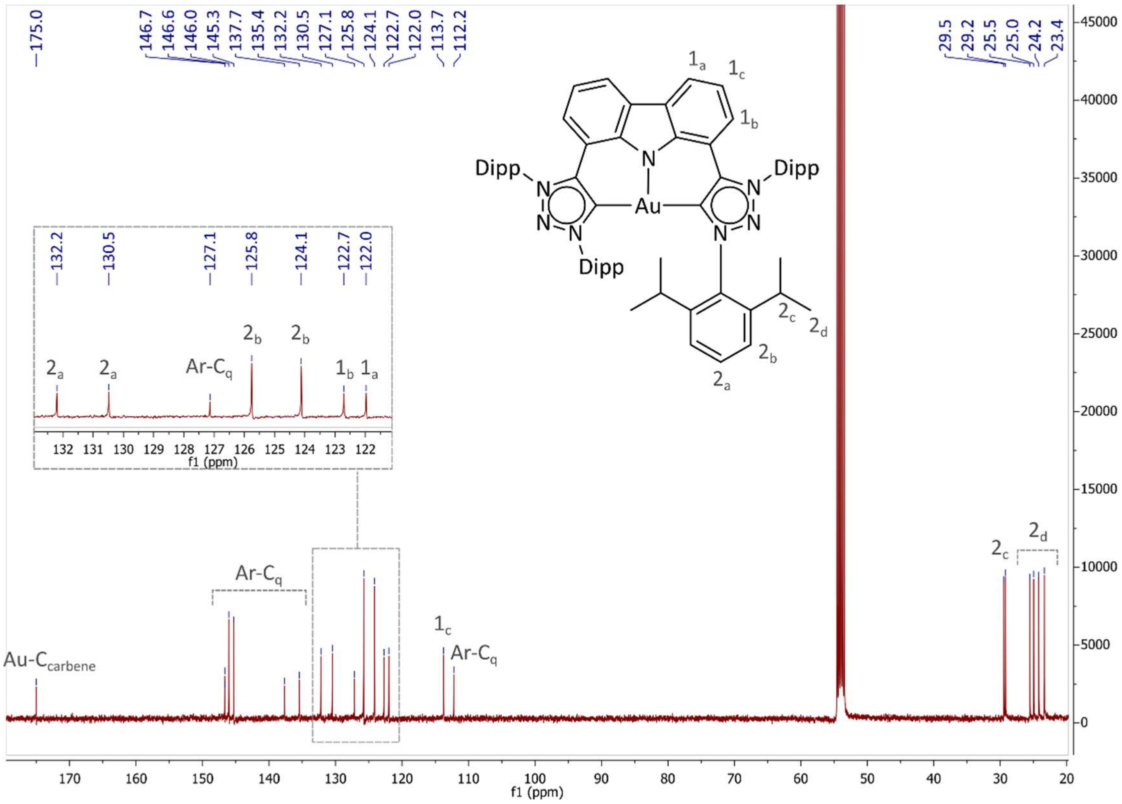

Figure S24. The $^{13}C\{^1H\}$ NMR spectrum of **4a** in CD_2Cl_2.

Synthesis of *[1,8-(bis(1,3-bis(2,6-diisopropylphenyl)-1,2,3-triazol-5-ylidene) carbazolide-CNC-chlorido-gold(III)] chloride*, 4b

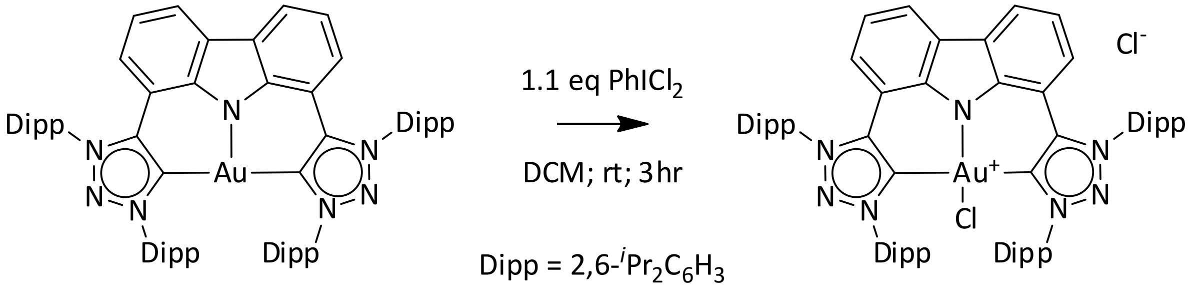

Scheme S12. Synthesis of **4b**.

To a solution of **4a** (0.10 g, 87.9 µmol, 1 eq) in anhydrous CH_2Cl_2, was added $PhICl_2$ (0.03 g, 94.6 µmol, 1.1 eq) and the red solution immediately turned light orange. Stirring was continued for 3 hours, after which the solvent was evaporated. The crude residue was washed with pentane and extracted with CH_2Cl_2, affording a light orange solid. Crystals suitable for XRD and in-vitro cell culture procedures were grown from a concentrated CH_2Cl_2 solution layered with toluene at room temperature. Yield: 87 mg (82%). M.p. 270 °C. ^1H NMR (300 MHz, $(CD_3)_2SO$) δ 8.60 (d, *J* = 7.6 Hz, 2H, Ar-$CH_{carbazole}$, H-1_a), 7.92 (t, *J* = 7.8 Hz, 2H, Ar-CH_{Dipp}, H-2_a), 7.73 (d, *J* = 7.9 Hz, 4H, Ar-CH_{Dipp}, H-2_b), 7.57 (t, *J* = 7.8 Hz, 2H, Ar-CH_{Dipp}, H-2_a), 7.35 (d, *J* = 7.8 Hz, 4H, Ar-CH_{Dipp}, H-2_b), 7.25 (dd, *J* = 7.8, 7.8 Hz, 2H, Ar-$CH_{carbazole}$, H-1_c), 6.97 (d, *J* = 7.9 Hz, 2H, Ar-$CH_{carbazole}$, H-1_b), 2.29 (sept, *J* = 6.8 Hz, 4H, ^{i}Pr-CH, H-2_c), 2.19 (sept, *J* = 7.0 Hz, 4H,

^{i}Pr-CH, H-2$_{c}$), 1.14 (d, *J* = 6.8 Hz, 12H, ^{i}Pr-CH$_3$, H-2$_{d}$), 1.10 (d, *J* = 6.8 Hz, 12H, ^{i}Pr-CH$_3$, H-2$_{d}$), 1.03 (d, *J* = 6.7 Hz, 12H, ^{i}Pr-CH$_3$, H-2$_{d}$), 0.93 (d, *J* = 6.7 Hz, 12H, ^{i}Pr-CH$_3$, H-2$_{d}$). ^{13}C {^{1}H} NMR (100 MHz, $(CD_3)_2SO$) δ 144.6, 143.9, 139.1, 137.2, 136.8, 134.9 (all Ar-C$_{q}$), 133.8 (Ar-CH$_{Dipp}$, C-2$_{a}$), 131.5 (Ar-CH$_{Dipp}$, C-2$_{a}$), 131.2 (Ar-C$_{q}$), 126.2 (Ar-CH$_{Dipp}$, C-2$_{b}$), 124.6 (Ar-C$_{q}$), 124.3 (Ar-CH$_{carbazole}$, C-1$_{a}$), 124.2 (Ar-CH$_{Dipp}$, C-2$_{b}$), 121.6 (Ar-CH$_{carbazole}$, C-1$_{b}$), 119.8 (Ar-CH$_{carbazole}$, C-1$_{c}$), 108.2 (Ar-C$_{q}$), 28.9 (^{i}Pr-CH, C-2$_{c}$), 28.7 (^{i}Pr-CH, C-2$_{c}$), 24.9, 24.1, 22.9, 22.7 (all ^{i}Pr-CH$_3$, C-2$_{d}$). ^{1}H NMR (400 MHz, CD_2Cl_2) δ 8.37 (d, *J* = 7.5 Hz, 2H, Ar-CH$_{carbazole}$, H-1$_{a}$), 7.84 (t, *J* = 7.9 Hz, 2H, Ar-CH$_{Dipp}$, H-2$_{a}$), 7.57 (d, *J* = 7.9 Hz, 4H, Ar-CH$_{Dipp}$, H-2$_{b}$), 7.54 (t, *J* = 7.7 Hz, 2H, Ar-CH$_{Dipp}$, H-2$_{a}$), 7.25 (d, *J* = 7.8 Hz, 4H, Ar-CH$_{Dipp}$, H-2$_{b}$), 7.18 (dd, *J* = 7.8, 7.8 Hz, 2H, Ar-CH$_{carbazole}$, H-1$_{c}$), 7.05 (d, *J* = 8.0 Hz, 2H, Ar-CH$_{carbazole}$, H-1$_{b}$), 2.29 (sept, *J* = 6.7 Hz, 4H, ^{i}Pr-CH, H-2$_{c}$), 2.22 (sept, *J* = 6.7 Hz, 4H, ^{i}Pr-CH, H-2$_{c}$), 1.17 (d, *J* = 6.9 Hz, 12H, ^{i}Pr-CH$_3$, H-2$_{d}$), 1.18 (d, *J* = 6.8 Hz, 12H, ^{i}Pr-CH$_3$, H-2$_{d}$), 1.09 (d, *J* = 6.9 Hz, 12H, ^{i}Pr-CH$_3$, H-2$_{d}$), 0.99 (d, *J* = 6.7 Hz, 12H, ^{i}Pr-CH$_3$, H-2$_{d}$). ^{13}C {^{1}H} NMR (100 MHz, CD_2Cl_2) δ 145.7, 144.9, 140.1, 138.3, 138.0, 136.0 (all Ar-C$_{q}$), 134.1 (Ar-CH$_{Dipp}$, C-2$_{a}$), 132.4 (Ar-CH$_{Dipp}$, C-2$_{a}$), 132.1 (Ar-C$_{q}$), 126.5 (Ar-CH$_{Dipp}$, C-2$_{b}$), 125.6 (Ar-C$_{q}$), 124.7 (Ar-CH$_{carbazole}$, C-1$_{a}$), 124.3 (Ar-CH$_{Dipp}$, C-2$_{b}$), 122.8 (Ar-CH$_{carbazole}$, C-1$_{b}$), 120.6 (Ar-CH$_{carbazole}$, C-1$_{c}$), 109.1 (Ar-C$_{q}$), 30.0 (^{i}Pr-CH, C-2$_{c}$), 29.9 (^{i}Pr-CH, C-2$_{c}$), 25.6, 24.9, 23.6, 23.4 (all ^{i}Pr-CH$_3$, C-2$_{d}$). Anal. Calcd for $C_{64}H_{74}N_7AuCl_2$ (+ 0.2 eq CH_2Cl_2) C 62.89, H 6.12, N 8.00. Found: C 62.90, H 6.08, N 7.73. ESI-(+)MS (Q-TOF) (positive mode, m/z): calcd for $[M+H-Cl]^+$: 1172.5354. Found: 1172.5390.

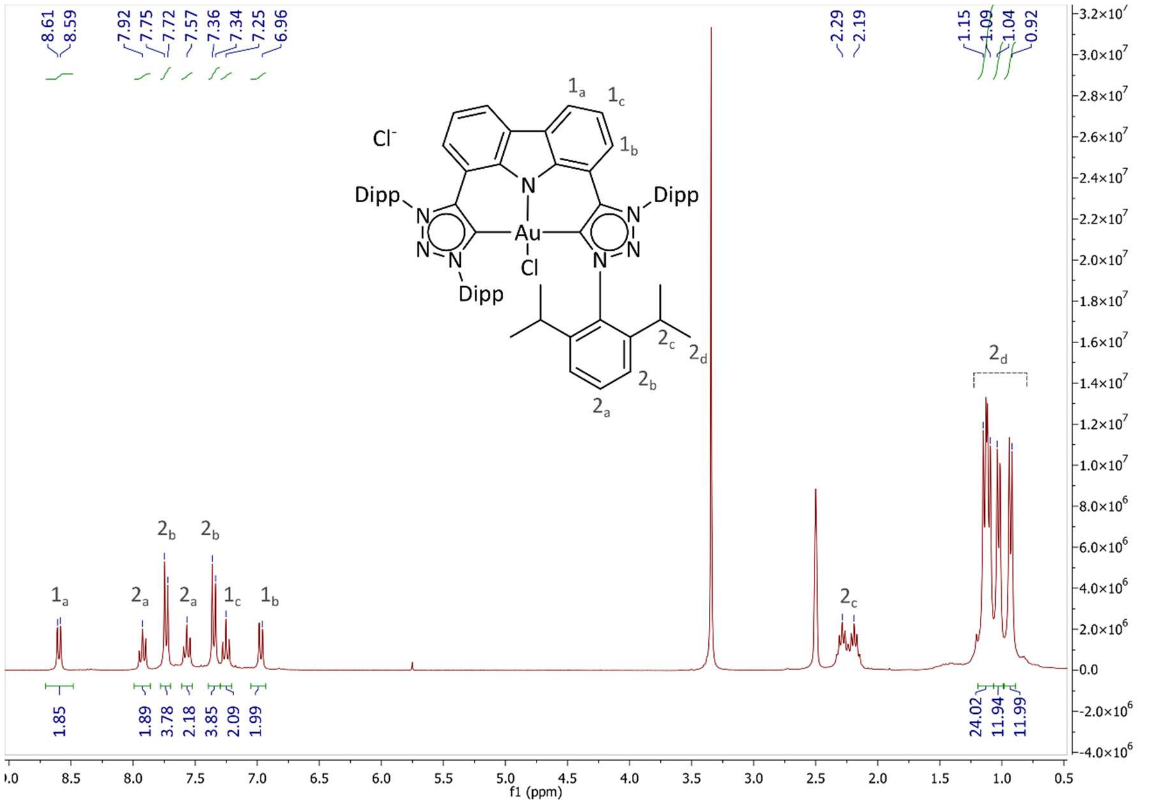

Figure S25. The ^{1}H NMR spectrum of **4b** in $(CD_3)_2SO$.

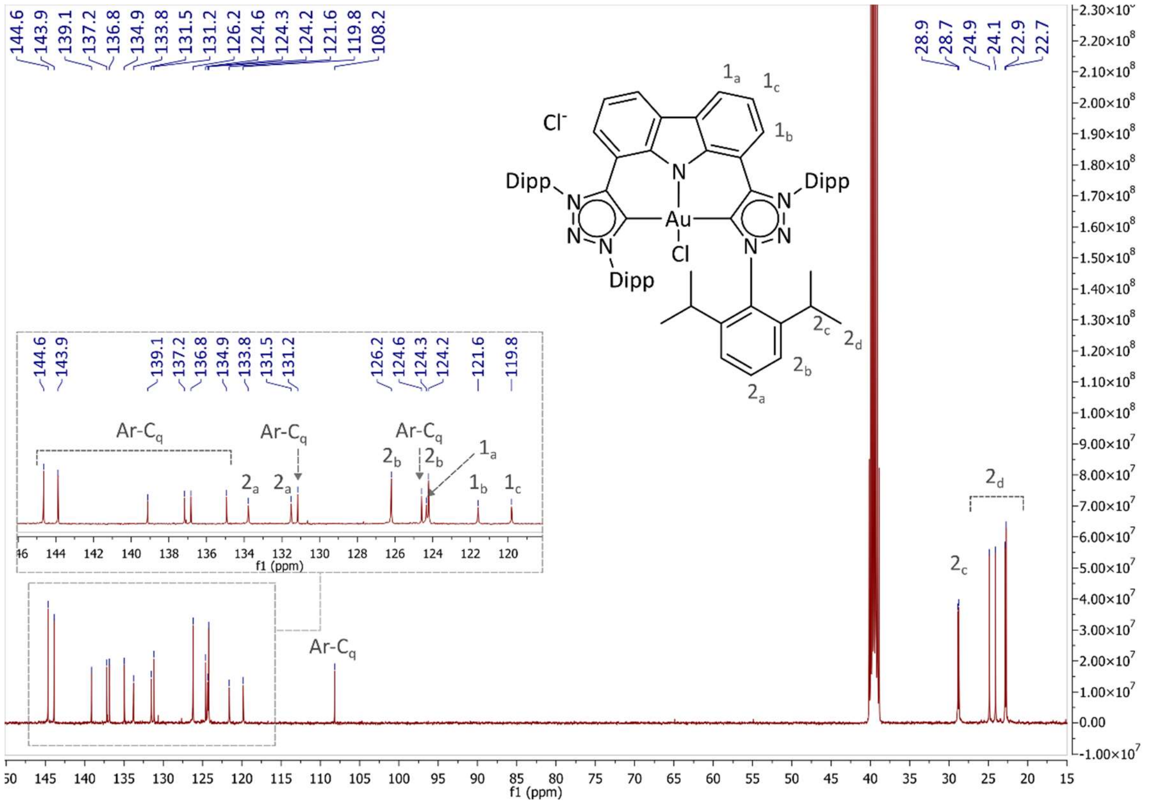

Figure S26. The $^{13}C\{^1H\}$ NMR spectrum of **4b** in $(CD_3)_2SO$.

Synthesis of *[3,6-di-tert-butyl-1,8-(bis(1,3-bis(2,6-diisopropylphenyl)-1,2,3-triazol-5-ylidene) carbazolide-CNC-chlorido-gold(III)] chloride*, 3b

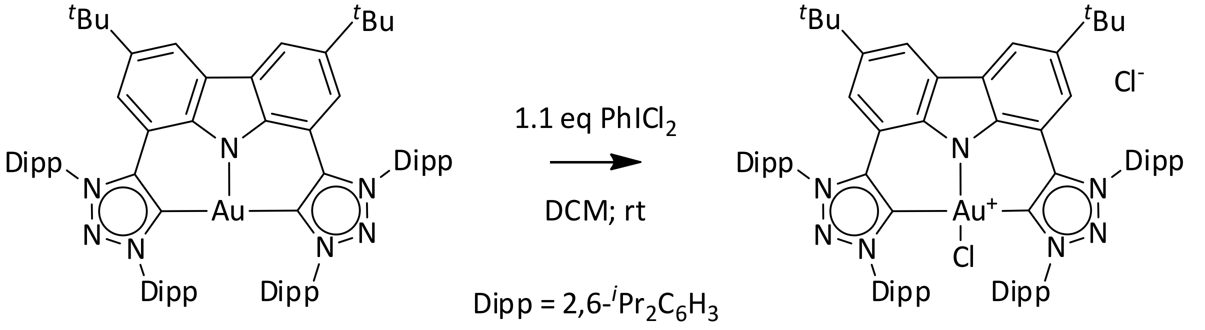

3a **3b** (71%)

Scheme S13. Synthesis of **3b**.

To a solution of **3a** (0.020 g, 15.9 µmol, 1 eq) in anhydrous CH_2Cl_2, was added $PhICl_2$ (4.84 mg, 17.6 µmol, 1.1 eq) and the red solution immediately turned light orange. Stirring was continued for 5 minutes, after which the solvent was evaporated. The crude residue was washed with pentane and extracted with CH_2Cl_2, affording a light orange solid. Crystals suitable for XRD were grown from a concentrated CH_2Cl_2 solution layered with toluene. Yield: 15 mg (71 %). ^1H NMR (500 MHz, $(CD_3)_2SO$) δ 8.74 (d, *J* = 1.6 Hz, 2H, Ar-$CH_{carbazole}$, H-1_a), 7.90 (t, *J* = 7.9 Hz, 2H, Ar-CH_{Dipp}, H-2_a), 7.77 (d, *J* = 7.9 Hz, 4H, Ar-CH_{Dipp}, H-2_b), 7.56 (t, *J* = 7.8 Hz, 2H, Ar-CH_{Dipp}, H-2_a), 7.33 (d, *J* = 7.8 Hz, 4H, Ar-CH_{Dipp}, H-2_b), 7.21 (d, *J* = 1.7 Hz, 2H, Ar-$CH_{carbazole}$,

H-1b), 2.27 (sept, J = 6.7 Hz, 4H, ^{i}Pr-CH, H-2c), 2.21 (sept, J = 6.8 Hz, 4H, ^{i}Pr-CH, H-2c), 1.14 (d, J = 6.7 Hz, 12H, ^{i}Pr-CH_3, H-2d), 1.11 (d, J = 6.8 Hz, 12H, ^{i}Pr-CH_3, H-2d), 1.09 (s, 18H, ^{t}Bu-CH_3), 1.03 (d, J = 6.8 Hz, 12H, ^{i}Pr-CH_3, H-2d), 0.93 (d, J = 6.7 Hz, 12H, ^{i}Pr-CH_3, H-2d). ^{13}C {^{1}H} NMR (125 MHz, $(CD_3)_2SO$) δ 144.7, 143.8, 142.3, 138.9, 139.1 (all Ar-C_q), 137.1 (Ar-CH_{Dipp}, C-2a), 137.0, 135.8, 135.1 (all Ar-C_q), 133.6 (Ar-CH_{Dipp}, C-2a), 131.5 (Ar-CH_{Dipp}, C-2a), 131.4 (Ar-C_q), 130.6 (Ar-CH_{Dipp}, C-2b), 127.7 (Ar-CH_{Dipp}, C-2b), 126.4 (Ar-CH_{Dipp}, C-2b), 124.6 (Ar-C_q), 124.1 (Ar-CH_{Dipp}, C-2b), 121.6 (Ar-$CH_{carbazole}$, C-1a), 119.1 (Ar-$CH_{carbazole}$, C-1b), 107.0 (Ar-C_q), 34.3 (^{t}Bu-C_q), 31.1 (^{t}Bu-CH_3), 28.9 (^{i}Pr-CH, C-2c), 28.8 (^{i}Pr-CH, C-2c), 24.9, 24.2, 22.9, 22.7 (all ^{i}Pr-CH_3, C-2d). ESI-(+)MS (Q-TOF) (positive mode, m/z): calcd for $[M-Cl]^+$: 1284.6606. Found: 1284.6627.

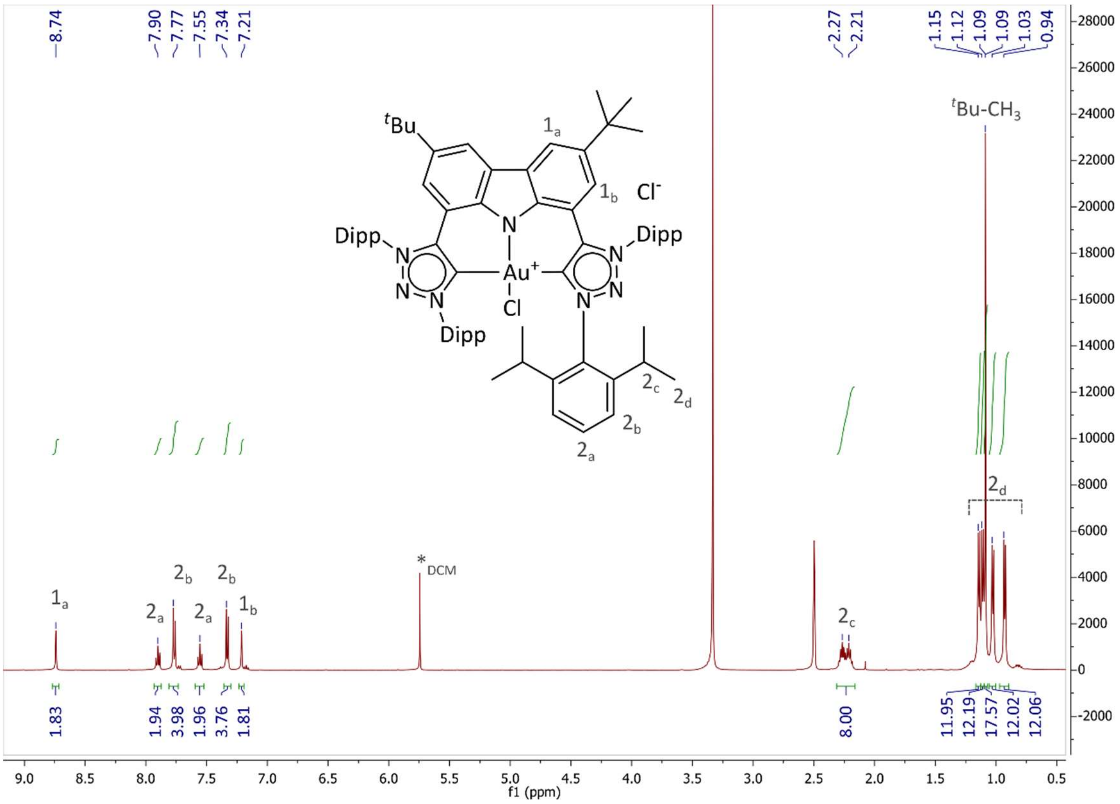

Figure S27. The ^{1}H NMR spectrum of **3b** in $(CD_3)_2SO$.

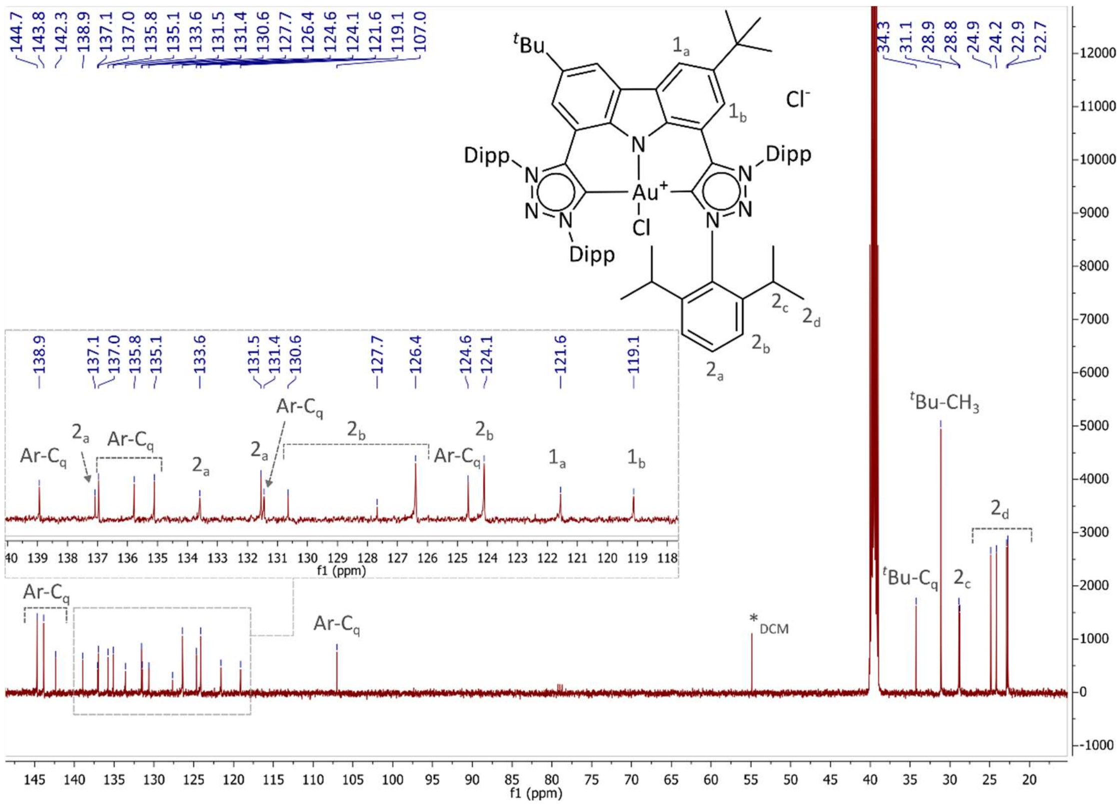

Figure S28. The $^{13}C\ \{^1H\}$ NMR spectrum of **3b** in $(CD_3)_2SO$.

Single-crystal X-ray diffraction data

1.5 Crystal structure data for the triazoles, P1, P4 and the triazolium salt, L2

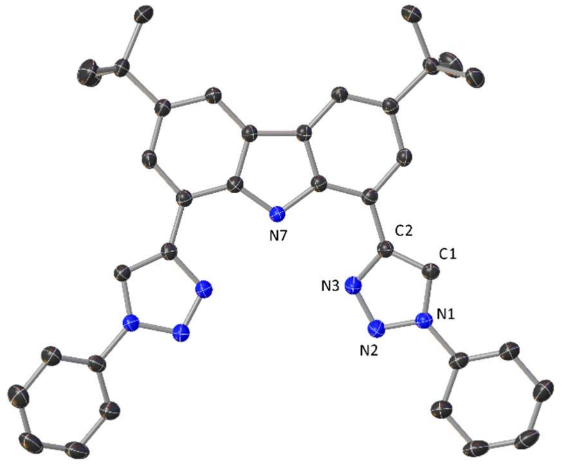

P1

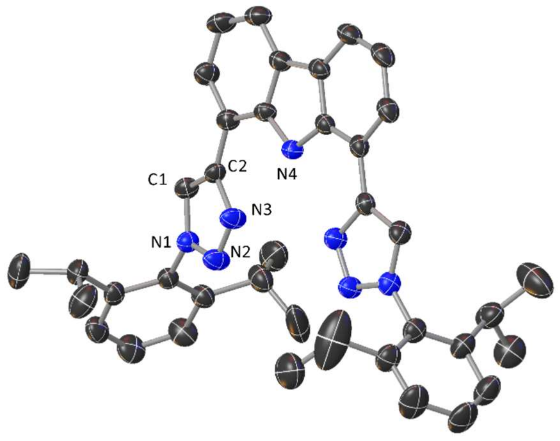

P4

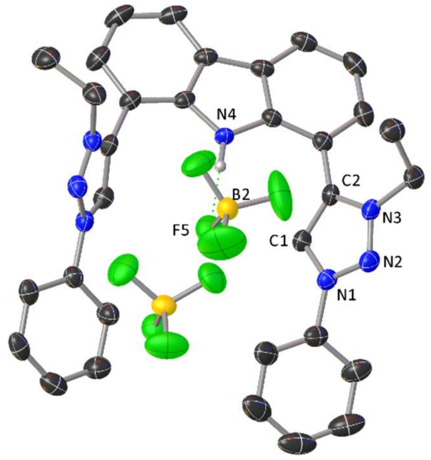

L2

Figure S29. Molecular structures of the triazoles **P1**, **P4** and the triazolium salt, **L2**, showing 50% probability ellipsoids and partial atom-numbering scheme. Hydrogens are omitted for clarity. Selected bond lengths (Å) and angles (°) for $\mathbf{K}^{\mathbf{\textit{t}Bu}}$: C1–C2 1.370(2), C2–N3 1.368(2), N2–N3 1.309(2), N1–N2 1.351(2), N1–C1 1.352(2), N1–C1–C2 105.07(15); $\mathbf{K}^{\mathbf{H}}_{\mathbf{dipp}}$: C1–C2 1.369(6), C2–N3 1.377(5), N2–N3 1.302(5), N1–N2 1.350(5), N1–C1 1.349(5), N1–C1–C2 105.3(4); $\mathbf{L}^{\mathbf{H}}$: C1–C2 1.364(3), C2–N3 1.364(2), N2–N3 1.316(2), N1–N2 1.322(2), N1–C1 1.351(2), N1–C1–C2 106.03(17).

Crystallographic Data:

P1: $C_{38}H_{37}Cl_6N_7$ (M = 804.44 g/mol): orthorhombic, space group *Pbca* (no. 61), a = 23.608(2) Å, b = 12.0460(12) Å, c = 27.425(3) Å, V = 7799.3(13) Å^3, Z = 8, T = 173.15 K, μ(MoKα) = 0.479 mm^{-1}, *Dcalc* = 1.370 g/cm^3, 110764 reflections measured (2.97° ≤ 2Θ ≤ 56.478°), 9591 unique (R_{int} = 0.0377, R_{sigma} = 0.0176) which were used in all calculations. The final R_1 was 0.0480 (I > 2σ(I)) and wR_2 was 0.1285 (all data). **CCDC 2048088**.

P4: $C_{83}H_{90}Cl_8N_{14}$ (M = 1567.28 g/mol): orthorhombic, space group $P2_12_12_1$ (no. 19), a = 12.6789(4) Å, b = 13.3047(4) Å, c = 48.1202(15) Å, V = 8117.3(4) Å^3, Z = 4, T = 173.15 K, μ(MoKα) = 0.331 mm^{-1}, $Dcalc$ = 1.282 g/cm^3, 80420 reflections measured (3.176° ≤ 2Θ ≤ 56.638°), 20135 unique (R_{int} = 0.0425, R_{sigma} = 0.0385) which were used in all calculations. The final R_1 was 0.0643 (I > 2σ(I)) and wR_2 was 0.1801 (all data). **CCDC 2048091**.

L2: $C_{32}H_{29}B_2F_8N_7$ (M = 685.24 g/mol): monoclinic, space group $P2_1$/n (no. 14), a = 14.5087(3) Å, b = 12.1465(3) Å, c = 17.8754(4) Å, β = 102.9728(12)°, V = 3069.78(12) Å^3, Z = 4, T = 173.15 K, μ(MoKα) = 0.124 mm^{-1}, $Dcalc$ = 1.483 g/cm^3, 37863 reflections measured (3.278° ≤ 2Θ ≤ 56°), 7404 unique (R_{int} = 0.0391, R_{sigma} = 0.0436) which were used in all calculations. The final R_1 was 0.0524 (I > 2σ(I)) and wR_2 was 0.1476 (all data). **CCDC 2048097**.

1.6 Crystal structures of the silver(I) complexes 1a and 2

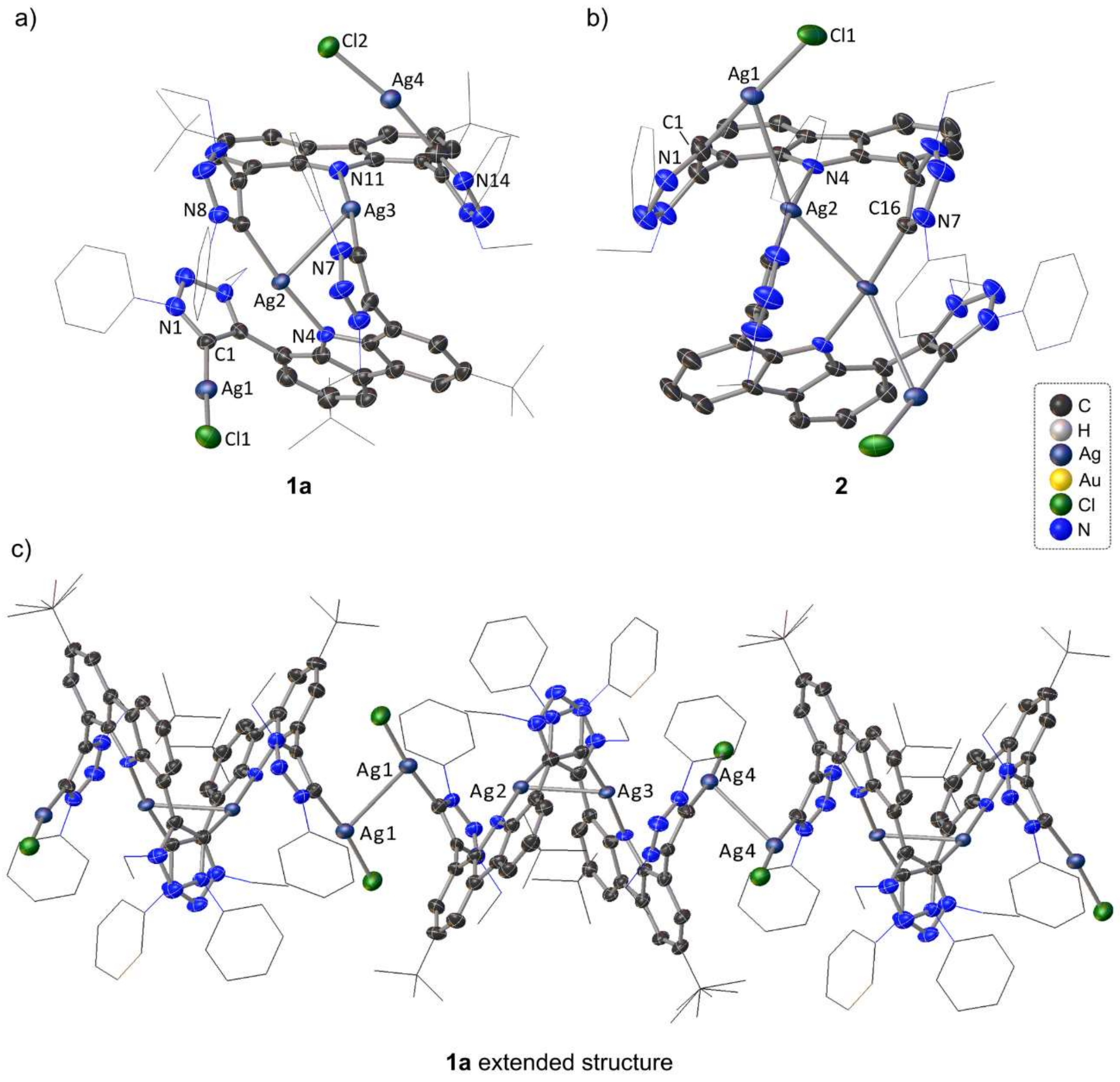

Figure S30. Molecular structures of a) **1a** and b) **2** showing 50% probability ellipsoids and partial atom-numbering schemes. Hydrogen atoms are omitted and the wingtip functionalities are displayed in wireframe format for clarity. Selected bond lengths (Å) and angles (°) for **1a**: Ag1–C1 2.065(7), Ag1–Cl1 2.328(2), Ag2–C41 2.061(6), Ag2–N4 2.099(5), Ag2–Ag3 2.9382(7), Ag1–Ag1′ 3.2811(12), Ag4–Ag4′ 3.2489(11), C1–Ag1–Cl1 173.1(2), N4–Ag2–C41 172.5(2). For **2**: Ag1–Ag2 3.201(6), Ag2–Ag2′ 2.872(8), Ag1–C1 2.090(5), Ag1–Cl1 2.337(18), Ag2–C16 2.075(5), Ag2–N4 2.099(4), C1–Ag1–Cl1 177.36(16), N4–Ag2–C16 172.93(19). (c) Extended structure of **1a** showing the polymeric nature of **1a** due to the metal-metal interactions between the Ag atoms of the triazolylidene Ag^{I}-chlorido between neighbouring structures.

Comments on the Synthesis and Structures of 1a and 2:

L1 was treated with silver(I) oxide and excess potassium chloride and the reaction terminated. A yellow powder was obtained in low yield (step a, Scheme 1, main paper). Complex formation was confirmed by the disappearance of both the triazolium (C5-H) and carbazole

(N-H) proton resonances in the ^{1}H NMR spectrum (Figure S19). The resonance signals for **1a** are doubled, as in the ^{1}H spectrum of **1b**. Single crystal XRD analysis (Figure S30a) revealed that **1a** is a tetranuclear monocarbene complex where all the Ag^{I} ions are bound to four triazolylidenes but two Ag^{I}-carbenes have chlorido ancillary ligands and the other two Ag^{I}-carbenes are bound to the nitrogen of the opposite carbazole ligand. The two carbazole moieties of **1a** are orientated in the same direction but twisted (by 131.8(6)°) and folded towards one another with a plane fold angle of 45.5(2)°. There is a slight difference in the length of the average Ag–$C_{carbene}$ bonds in **1a**, depending on whether the ancillary ligand is chloride (2.085(8) Å) or a N atom (2.067(6) Å). The average Ag–$C_{carbene}$ bond length of 2.076(8) Å is comparable to the related N3-alkylated Ag^{I} bis(trz) complexes (2.075(7) Å) reported by Keske *et al.*,[24] and a N3-alkylated Ag^{I} trz with a pyrrole as ancillary ligand (averaged 2.074 Å), described by Cai *et al*.[25] Notably, the solid state structure revealed both supported argentophilic interactions (enabled by linker ligands) as well as unsupported interactions between neighboring molecules that result in one-dimensional polymers (see ESI, Figure S30).[26]

The solid-state structure of **1a** revealed strong Ag–Ag interactions of 2.9381(7) Å (compared to the van der Waals radii of 3.440 Å for Ag(0)).[27] The metal-metal interactions of **1a** extend to the Ag-atoms of the neighbouring structures, specifically to the silver(I)-chlorido moiety resulting in a polymeric structure (Figure S30c). Although these intermolecular Ag–Ag interactions are markedly longer (averaged 3.265 Å) than the intramolecular Ag–Ag bond length (2.9381(7) Å), they are still considered significant, albeit weaker, as they are shorter than 3.440 Å but longer than the Ag–Ag distance of 2.88 Å in metallic silver.[27] Further differences in the metal–metal interaction between the Ag atoms of **2** compared to **1a** are also noted. For instance, the Ag1–Ag2 (Ag–Cl vs. Ag–N) bond length of 3.201(6) Å is considerably longer than the Ag2–Ag2 distance (both Ag–N) of 2.872(8) Å in **2**, whereas in **1a** there was no noticeable interaction between the Ag1 and Ag2 atoms, but rather to the neighbouring Ag atom of the $C_{carbene}$–Ag–Cl moiety (Ag1–Ag1 interaction). Metal–metal interactions between neighbouring structures are not observed for **2**.

The Ag^{I} complex of **L2** was also synthesized, i.e., compound **2**, and is similar in structure to **1a** (Figure S30b), except for intermolecular interactions (*vide infra*). The carbene resonances for **2** appear as two sets of doublets of doublets at δ_C 164.5 and δ_C 164.4 ppm (Figure S22). The carbene carbon chemical shift for **2** is slightly upfield compared to Crudden's Ag^{I} bis(trz) derivative (δ_C 172 ppm)[27] and the N1,N3-diarylated T-shaped Ag^{I} complex (δ_C 177.4 ppm),[27] but is comparable to the carbene chemical shift of a ferrocenyl N1,N3-diarylated Ag^{I} bis(trz) derivative (167.5 ppm).[27] Complex **2** is surprisingly unstable compared to **1a** and decomposes within a few weeks at room temperature, changing from a light-orange powder to a dark brown oil. No change in appearance was observed for **1a** over longer periods of time.

Crystals of **2** were grown from a concentrated CH_2Cl_2 solution layered with toluene. Like **1a**, the molecular structure of **2** consists of four trz Ag^I moieties whereby two Ag^I ions have ancillary chlorido ligands, while the remaining two are coordinated to the carbazole amido group of the opposite ligand. The two carbazole moieties are orientated in the same direction with a plane angle of 49.5(2)° and twisted (149.9(6)°) relative to one another. The average $Ag–C_{carbene}$ bond length of **2** (2.083(5) Å) is similar to that of **1a** (2.076(8) Å). The $Ag–C_{carbene}$ bond length where the chloride ion is an ancillary ligand is slightly longer (2.090(5) Å) than the $Ag–C_{carbene}$ bond length when the nitrogen is the ancillary ligand (2.075(5) Å). The $C_{carbene}$–Ag–X bond angle is also different: for X = Cl, the bond angle approaches linearity at 177.36(16)°, while for X = N, the bond angle (172.93(19)°) is akin to that observed for **1a** (171.9(2)°). Metal–metal interactions between crystal lattice neighbors are absent for **2**.

Crystallographic Data:

1a: $C_{87}H_{97}Ag_4Cl_{11}N_{14}O$ (M = 2176.21 g/mol): triclinic, space group *P*-1 (no. 2), *a* = 15.3749(6) Å, *b* = 17.8182(7) Å, *c* = 19.1815(7) Å, *α* = 97.825(2)°, *β* = 112.556(2)°, *γ* = 90.804(2)°, *V* = 4795.0(3) Å3, *Z* = 2, *T* = 173.15 K, μ(MoKα) = 1.162 mm-1, *Dcalc* = 1.507 g/cm3, 86531 reflections measured (2.876° ≤ 2Θ ≤ 56.95°), 23946 unique (R_{int} = 0.0506, R_{sigma} = 0.0432) which were used in all calculations. The final R_1 was 0.0728 (I > 2σ(I)) and wR_2 was 0.2092 (all data). **CCDC 2048090**.

2: $C_{71}H_{60}Ag_4Cl_2N_{14}$ (*M* = 1611.71 g/mol): tetragonal, space group $P4_32_12$ (no. 96), *a* = 14.6293(6) Å, *c* = 30.1142(14) Å, *V* = 6444.9(6) Å^3, *Z* = 4, *T* = 173.15 K, μ(MoKα) = 1.336 mm^{-1}, *Dcalc* = 1.661 g/cm^3, 91680 reflections measured (5.57° ≤ 2Θ ≤ 56.654°), 8030 unique (R_{int} = 0.0454, R_{sigma} = 0.0239) which were used in all calculations. The final R_1 was 0.0357 (I > 2σ(I)) and wR_2 was 0.0750 (all data). **CCDC 2048092**.

1.7 Crystal structures of the gold(I) complexes 1b, 1c and 4a

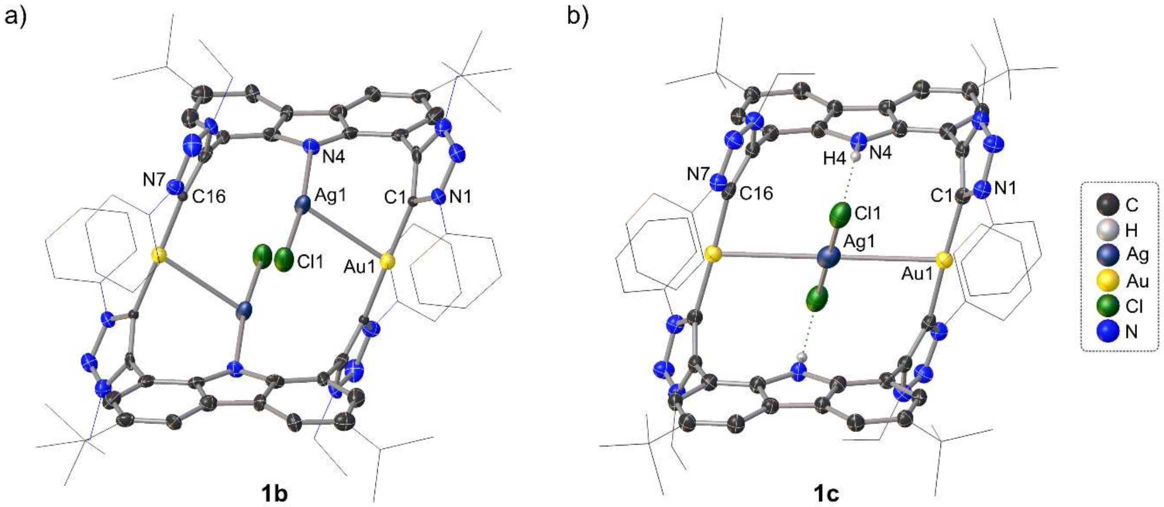

Figure S31. Molecular structures of a) **1b** and b) **1c** showing 50% probability ellipsoids and partial atom-numbering schemes. Hydrogens (except for the carbazole-NH in (b)) are omitted and the wingtip functionalities are displayed in wireframe format for clarity. Selected bond lengths (Å) and angles (°) for **1b**: Ag1–Au1 2.924 (11), Au1–C1 1.965(9), Au1–C16 1.964(9), Ag1–N4 2.119(9), C1–Au1–C16 176.7(4). For **1c**: Ag1—Au1 3.2209(9), Au1–C1 2.024(4), Au1–C16 2.019(4), C1–Au1–C16 176.4(17).

Comments on the Structures of 1b and 1c:

Single crystal X-ray diffraction (XRD) studies on yellow crystals grown from a concentrated CH_2Cl_2 solution layered with pentane revealed the structure of the zwitterionic Au^I bis(carbene) complex **1b** with Ag^I chloride bound to the carbazole amide (Figure S31a). The average Au–$C_{carbene}$ bond length of 1.965(9) Å is in the range of previously reported N3-alkylated Au^I bis(triazolylidenes) with an expected near-linear $C_{carbene}$–Au–$C_{carbene}$ angle of 176.7(4)°.[28] The distance between the Ag and Au atom is 2.924(11) Å and is considered a short interaction since the sum of the covalent radii of Au and Ag is 2.89 Å. The interaction might explain the slight deviation in the linear geometry of the Au^I ion.[29] Complex **1b** is, however, not stable in solution and within a week, decomposes to crystallize as the dicationic Au^I bis(carbene) complex **1c** (route c, Scheme 1, main paper) with protonated carbazoles and $[AgCl_2]^-$ counterions. Attempts to record the NMR spectra of these crystals were futile as the $[AgCl_2]^-$ counterion significantly reduced the solubility of this complex. Both mass spectrometry and CHN microanalysis confirmed the eventual formation of **1c** in the solid state. Compared to **1b**, the average Au–$C_{carbene}$ bond length of **1c** (Figure 1b) slightly increased to 2.022(4) Å. There is no significant deviation in the $C_{carbene}$–Au–$C_{carbene}$ bond angle of 176.4(17)° compared to the same bond angle reported for **1b** (176.7(4)°). The average Ag–Au

bond length between the Au atoms of the carbenes and the Ag atom of the dichlorosilver(I) counterion have significantly increased to 3.2209(19) Å compared to the bond lengths of 2.924(11) Å in **1b**. Species **1b** presumably represents a stable intermediate during the carbene transfer from Ag^{I} to Au^{I} and indicates that the Ag^{I}–$C_{carbene}$ bonds are more labile than the Ag^{I}–N_{amido} bond. The presence of the metal–metal interaction between Ag and Au in **1b** might aid in the stabilization of this intermediate and possibly facilitates the abstraction of the Ag^{I} ion from the carbazole nitrogen to form **1c**.

Crystallographic Data:

1b: $C_{83.5}H_{93}Ag_2Au_2Cl_{11}N_{14}$ (M = 2292.34 g/mol): triclinic, space group P-1 (no. 2), a = 13.3806(3) Å, b = 15.7343(4) Å, c = 22.0730(5) Å, α = 79.6820(10)°, β = 77.5350(10)°, γ = 85.1820(10)°, V = 4459.23(18) $Å^3$, Z = 2, T = 173.15 K, μ(MoKα) = 4.094 mm^{-1}, $Dcalc$ = 1.707 g/cm^3, 164663 reflections measured (2.99° ≤ 2Θ ≤ 56.832°), 22046 unique (R_{int} = 0.1075, R_{sigma} = 0.0688) which were used in all calculations. The final R_1 was 0.0369 (I > 2σ(I)) and wR_2 was 0.0955 (all data). **CCDC 2048095**.

1c: $C_{41.5}H_{46}AgAuCl_4N_7$ (M = 1089.49 g/mol): monoclinic, space group $C2/c$ (no. 15), a = 20.4625(11) Å, b = 18.4479(12) Å, c = 22.5829(13) Å, β = 91.071(4)°, V = 8523.3(9) $Å^3$, Z = 8, T = 173.15 K, μ(MoKα) = 4.188 mm^{-1}, $Dcalc$ = 1.698 g/cm^3, 48833 reflections measured (2.972° ≤ 2Θ ≤ 53.112°), 8787 unique (R_{int} = 0.2369, R_{sigma} = 0.2648) which were used in all calculations. The final R_1 was 0.0666 (I > 2σ(I)) and wR_2 was 0.1362 (all data). **CCDC 2048089**

4a: $C_{65}H_{76}AuCl_2N_7$ (M = 1223.19 g/mol): tetragonal, space group P-42$_1c$ (no. 114), a = 21.9064(10) Å, c = 24.9397(12) Å, V = 11968.3(12) $Å^3$, Z = 8, T = 173(2) K, μ(MoKα) = 2.593 mm^{-1}, $Dcalc$ = 1.358 g/cm^3, 178479 reflections measured (5.88° ≤ 2Θ ≤ 55.998°), 14430 unique (R_{int} = 0.0538, R_{sigma} = 0.0250) which were used in all calculations. The final R_1 was 0.0232 (I > 2σ(I)) and wR_2 was 0.0508 (all data). **CCDC 2048093**.

1.8 Crystal structure data for the gold(III) complexes, 3b and 4b

4b: $C_{163}H_{188}Au_2Cl_4N_{14}$ (*M* = 2879.00 g/mol): monoclinic, space group *P*2$_1$/n (no. 14), *a* = 15.2299(5) Å, *b* = 22.0990(8) Å, *c* = 21.7606(7) Å, β = 91.4767(11)°, *V* = 7321.4(4) Å^3, *Z* = 2, *T* = 173(2) K, μ(MoKα) = 2.130 mm^{-1}, *Dcalc* = 1.306 g/cm^3, 96491 reflections measured (2.626° ≤ 2Θ ≤ 56.808°), 18313 unique (R_{int} = 0.0786, R_{sigma} = 0.0757) which were used in all calculations. The final R_1 was 0.0396 (I > 2σ(I)) and wR_2 was 0.0914 (all data). **CCDC 2048096**.

3b: $C_{72}H_{90}AuCl_2N_7$ (*M* =1321.37 g/mol): monoclinic, space group *C*2/*c* (no. 15), *a* = 28.613(3) Å, *b* = 18.9492(18) Å, *c* = 19.3194(19) Å, β = 118.325(2)°, *V* = 9220.6(16) Å^3, *Z* = 4, *T* = 173.15 K, μ(MoKα) = 1.687 mm^{-1}, *Dcalc* = 0.952 g/cm^3, 25765 reflections measured (3.048° ≤ 2Θ ≤ 49.998°), 8102 unique (R_{int} = 0.0727, R_{sigma} = 0.0836) which were used in all calculations. The final R_1 was 0.0631 (I > 2σ(I)) and wR_2 was 0.1628 (all data). **CCDC 2048094**.

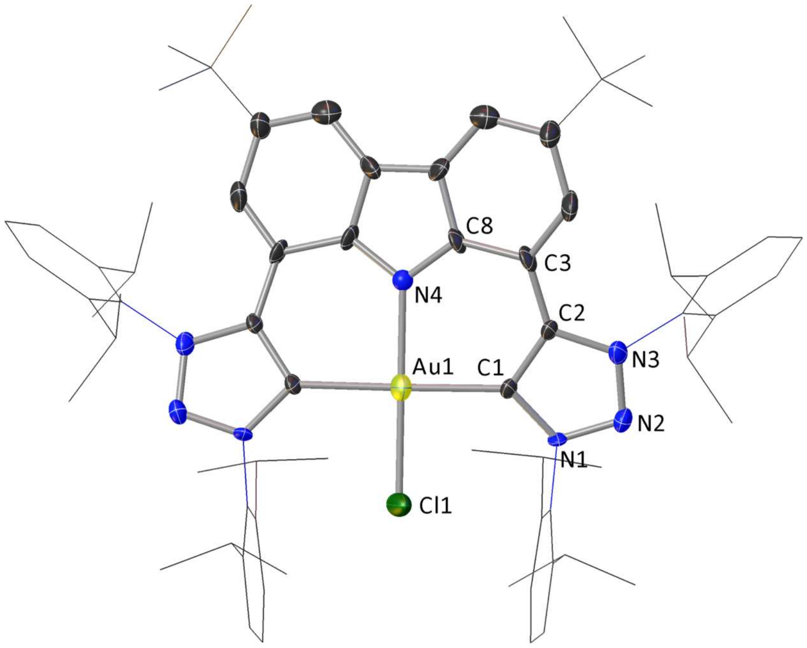

Figure S32. The molecular structure of **3b** showing 50% probability ellipsoids and partial atom-numbering scheme. Hydrogen atoms and counterions are omitted for clarity. Selected bond lengths (Å) and angles (°) for **3b**: Au1–C1, 2.066(5); Au1–N4, 2.045(7); Au1–Cl1, 2.235(3); C1–Au1–C1’, 179.0(4); N4–Au1–Cl1, 180.0; C1–Au1–Cl1, 90.5(2); C1–Au1–N4, 89.5(2); C1–C2–C3–C8, −11.7(10).

Comment on X-ray Structure:

3b is also square planar with the associated bond angles around the Au^{III} ion close to 90° ($C_{carbene}$–Au–N_{amido}, 89.5(2)°; $C_{carbene}$–Au–Cl, 90.5(2)°) and 180° ($C_{carbene}$–Au-$C_{carbene}$, 179.0(4)°; N–Au–Cl, 180.0°). The asymmetric unit of the molecular structure is half of that shown in where the plane of symmetry is down the N4–Au1–Cl1 plane. The Au–$C_{carbene}$ bond length of 2.066(5) Å and the Au–N_{amido} bond length of 2.045(7)° are both longer than that reported for **4b** (Au–$C_{carbene}$, 2.047 Å; Au-N, 2.010(3) Å) and are within the range reported previously for Au–$C_{carbene}$ and Au–N_{amido} bond lengths.[12,30] Similar to **4b**, the $C_{carbene}$–Au–$C_{carbene}$ plane is twisted in relation to the carbazole moiety. Due to the symmetry in the solid-state structure,

both triazolylidene rings are orientated 11.7(10)° above and below the plane of the carbazole moiety, respectively.

Cell maintenance and in-vitro procedures

Distilled water, general glassware and pipette tips were sterilized via autoclave sterilization (120 °C at 15 psi for 20 min) and solutions were filtered-sterilized (0.22 µM pore size). The incubator and laboratory surfaces were regularly disinfected with 1% SDS (sodium dodecyl sulphate), 10% bleach and 70% ethanol.

In vitro studies were carried out on the human breast cancer cell line, MDA-MB-231[31] and the non-tumourigenic endothelial cell line, EA.hy926. Both cell lines are commercially available from the American Type Culture Collection Cell Line Bank. Both cell lines were grown and maintained in sterile 25 or 75 cm^2 tissue culture flasks in a humidified atmosphere at 37 °C and 5% CO_2 using Iscove's Modified Dulbecco's Medium (IMDM) supplemented with 10% heat inactivated foetal calf serum, 100 U/mL penicillin G, 100 µg/mL streptomycin and 250 µg/L fungizone. Growth medium was replaced every two to three days and if confluent, the cells were trypsinized; cells were incubated with trypsin (1–3 min at 37 °C) following the removal of the growth medium and several washes with 0.01 M (1x) PBS (phosphate buffered solution). Thereafter the flasks were tapped against gloved hands and inspected under the microscope to observe if the cells were rounded and detached from the cell culture flask surface. The trypsinization was quenched with the addition of medium and collected in 15 mL tubes. The suspension was centrifuged at 3000 rpm for 5 minutes and the supernatant was removed. The pellet was resuspended in fresh medium and was either divided into subcultures, counted for seeding purposes or frozen away in cryotubes at −78 °C (1 000 000 cells/mL in 10% DMSO in IMDM).

1.9 Seeding for in-vitro procedures

The pellet obtained from a trypsinized cell suspension was resuspended in fresh medium and 20 µL of this suspension was added to 60 µL medium and 20 µL trypan blue dye. The stained cells (10 µL) were counted on a haemocytometer and the average count of 5 or 10 squares were taken, multiplied by the dilution factor (×5) and 10 000 to obtain the number of viable cells per millilitre. The volume of cell suspension required to ensure the right number of cells per experiment is obtained by dividing the required number of cells by the cells counted per millilitre

1.10 Crystal violet assay

Stock solutions (50 mM) of the compounds were made in DMSO and test concentrations (20 µM–0.3125 µM) for the CVS assay were prepared by diluting the compounds in cell culture medium. The vehicle control represented the highest DMSO concentration (never exceeding 0.04%) relevant to the test conditions. Cells were seeded at 5000 cells per well in a 96 well plate and allowed to adhere to the plate overnight in a humidified incubator at 37 °C and 5% CO_2. The attachment medium was discarded, and the cells were exposed to incremental

concentrations of the compounds for 48 hrs in a humidified incubator at 37 °C and 5% CO_2. After 48 hrs, the medium was removed and 100 µL of 1% glutaraldehyde was added to each well and incubated for 15 min at room temperature, followed by the removal of glutaraldehyde and the addition of 0.1% crystal violet solution (0.1 g of gentian violet dissolved in 1% ethanol solution). The plates were incubated for 30 min at room temperature. The excess dye was removed and after the plates were dried, the dye was dissolved by the addition of 200 µL 0.2% Triton-X and allowed to incubate on a plate shaker for 60–90 min, after which 100 µL of the solubilised dye was transferred to a 96-well reading plate. The absorbances of each well were measured at 570 nm using a ELX800 Universal Microplate Reader. Six data points were obtained for a single concentration and three replicate experiments were performed. The absorbance values were corrected against the background (absorbance of empty wells) and were expressed as percentage cell viability. The percentage cell viability is calculated by expressing the absorbance of the treated cells as a percentage of the untreated cells. The IC_{50} values were obtained by using the non-linear regression analysis of *GraphPad Prizm 5* software. The IC_{50} values are reported with an associated SEM (standard error from the mean), which indicates the uncertainty of the mean measurement and takes into account both the standard deviation and sample size.[32]

1.11 Preliminary screening of L3, L4, 3a, 3b , 4a and 4b

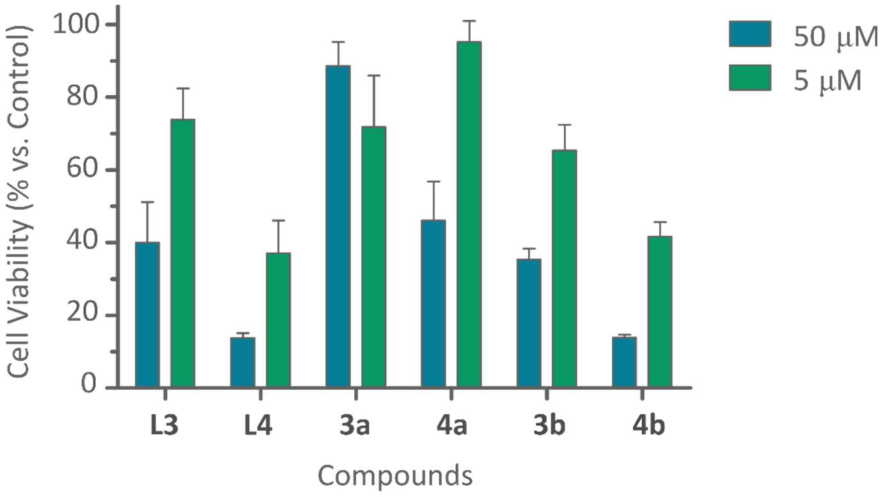

Figure S33. The percentage cell viability (determined by CVS assay) of MDA-MB-231 cells treated against the CNC gold(I/III) pincer complexes and their corresponding precursor ligand salts at 5 µM and 50 µM after 48 hrs of drug exposure compared to the control (0.1% DMSO). Data represent the average ± standard error from mean for 6 replicates of one experiment.

1.12 Dose-response curves of L4 and 4b

a)

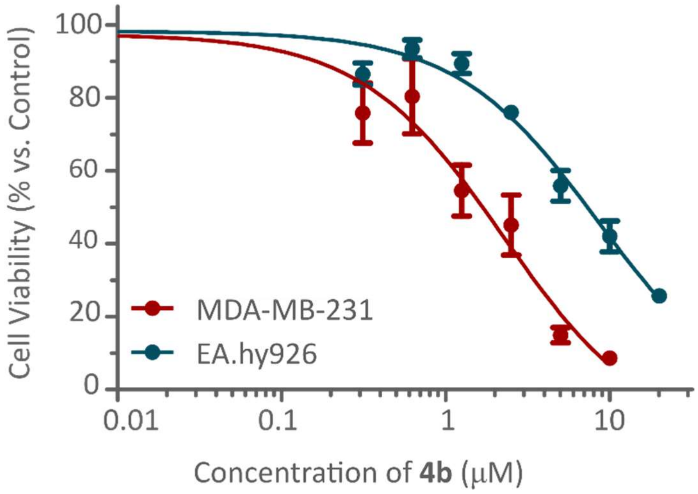

b)

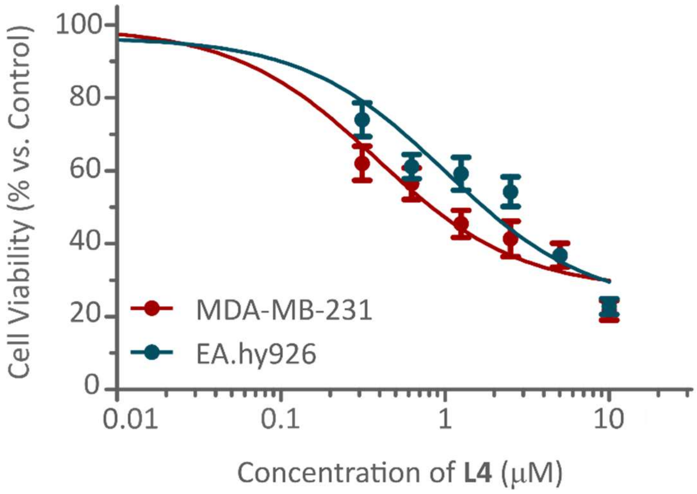

Figure S34. Dose-response curve of (top) **4b** and (bottom) **L4** against MDA-MB-231 cells (red) and EA.hy926 (blue) cells. The graphs show the percentage cell viability compared to the control (0.02% DMSO except for **4b** and EA.hy926, which had 0.04% DMSO as vehicle control) as response to increasing concentrations of **4b** and **L4**. Data represent the average ± standard error from mean (SEM) for three independent experiments, each with 6 replicates.

1.13 Stability of 4b in DMSO-d6

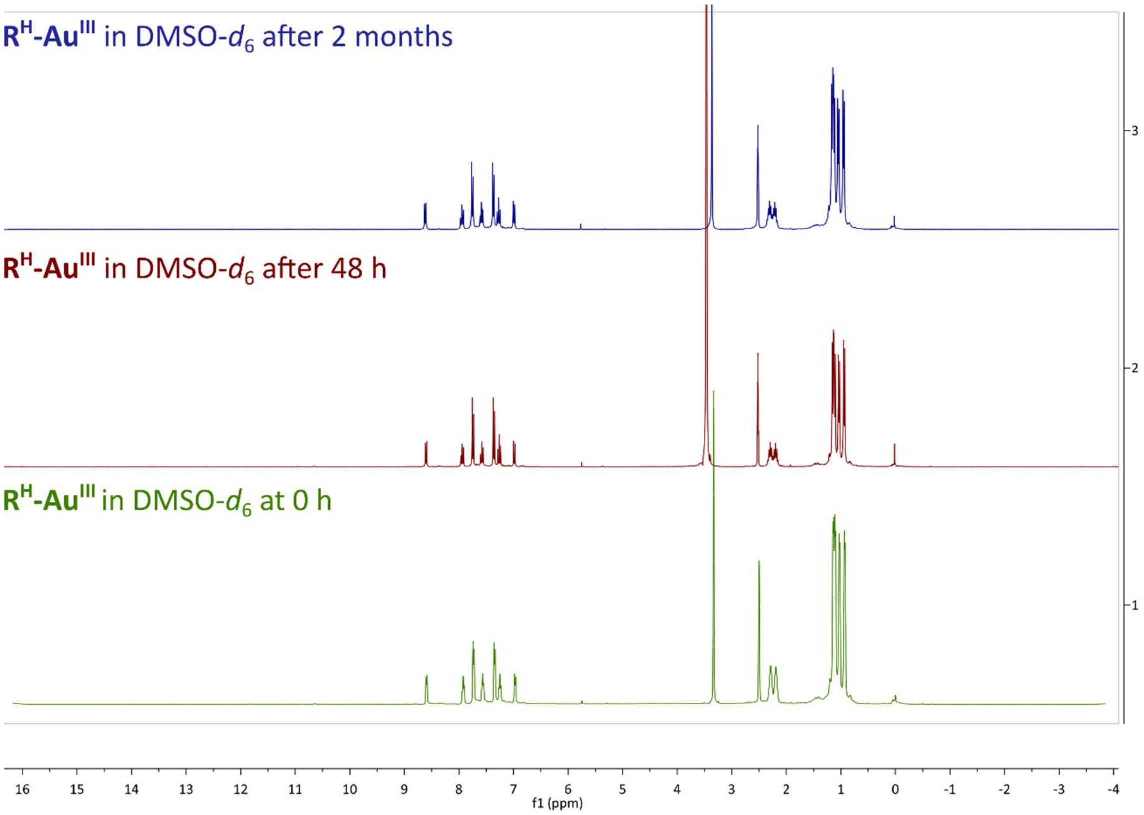

Figure S35. The ^{1}H NMR spectra of **4b** in $(CD_3)_2SO$ at 0 hr (green), 48 hr (red) and 2 months (blue).

Bioinorganic methodologies

1.14 Electronic structure of 4b

The molar absorptivities at the wavelength maxima of **4b** in DMSO and in 1x PBS (10% DMSO) were determined according to the Beer-Lambert Law. For the Beer-Lambert titration a 2.0×10^{-4} M **4b** sample was prepared in a quartz cuvette and subsequently diluted to known lower concentrations of 1.0×10^{-4} M, 5×10^{-5} M, 2.5×10^{-5} M, 1.0×10^{-5} M and 5×10^{-6} M. The absorbances at 303, 336, 363 and 398 nm were plotted as a function of the concentration of **4b** and the slopes (molar absorptivity) were calculated with linear regression analysis.

The TD-DFT calculations were done according to the computational details reported for the previously reported CNC gold(III) fluorido complex.[30] The **4b** structure was optimized from the crystal structure data at the B3LYP-D3[33–36] level of theory using def2-SVP[37] basis set for all atoms in Gaussian 09 revision E.[38] At the same DFT level, TD-DFT calculations were conducted using the polarizable continuum model (PCM)[39–41] for DMSO and the first 60 excited states were considered. The DFT-calculated spectrum was visualised with GaussSum 3.0.[42]

Table S1. Experimental UV-vis absorption maxima and DFT-calculated absorbance data with major contributions to key transitions for **4b** in DMSO.

Electronic absorption bands (nm) (ε / M^{-1} cm^{-1})	Calculated electronic absorption bands (λ_{max}/nm) (f, oscillator strength)	Major Contributions (> 20%) to electronic transitions
n.o[a]	270 (0.1293)	HOMO−1 → LUMO+4
303 (14036)	288 (0.1875)	HOMO−11 → LUMO<br>HOMO−9 → LUMO
336 (8699)	325 (0.0725)<br>338 (0.0693)	HOMO−1 → LUMO+2<br>HOMO−1 → LUMO+1
363 (8798)	374 (0.1467)	HOMO → LUMO+2
398 (7494)	390 (0.0571)	HOMO → LUMO+1

[a] Falls outside the measured window

The higher energy transitions of 270 nm and 288 nm (the latter corresponding to the experimental peak observed at 303 nm) involve the excitation of an electron from HOMO-1 to LUMO+4 and from both HOMO−9 and HOMO−11 to the frontier LUMO, respectively. More specifically, the transition at 271 nm is attributed to the transfer of an electron from a π bonding orbital localized on the carbazole moiety to an antibonding (π*) orbital localized on the carbazole ring system and triazolylidene rings (ILCT $\pi \rightarrow \pi$*). The main transition contributing to the peak at 303 nm involves the excitation from the Dipp wingtip groups (HOMO−9) and the conjugated ring system spanning across the carbazole and triazolylidenes (HOMO−11) to the LUMO located on the Au^{III} ion (σ* MO derived from the $5dx^2 - y^2$ atomic orbital) and the nearby bonding atoms i.e., the nitrogen from the carbazole amido, the two carbenes and the chloride ancillary ligand. The band is therefore assigned as a ligand to metal charge transfer (LMCT $\pi \rightarrow \sigma$*).

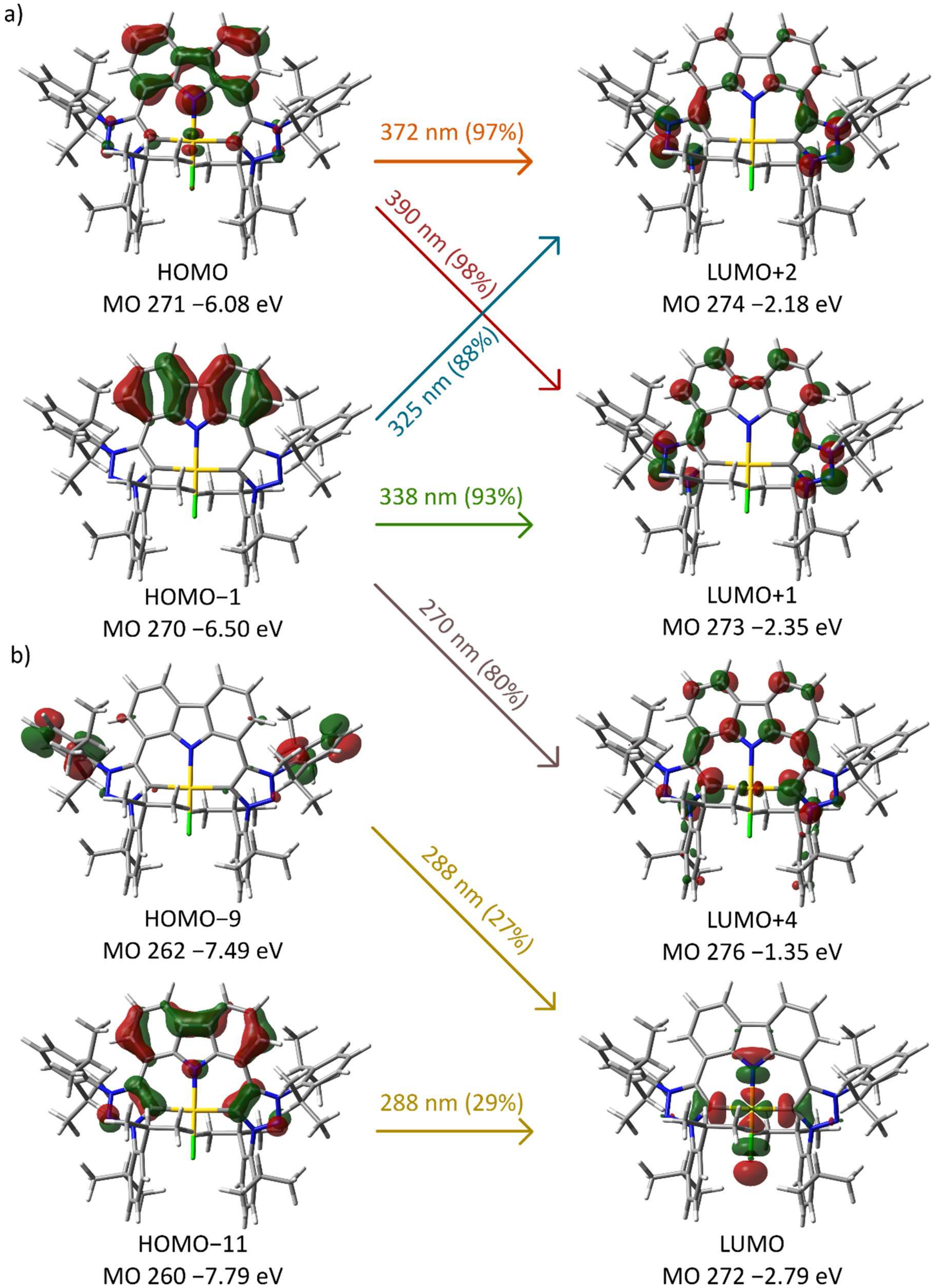

Figure S36. Views of the molecular orbitals for **4b** that contribute mainly to the predicted a) lower and b) higher energy transitions in the UV-vis spectrum as calculated by Gaussian 09 revision E at B3LYP-D3/Def-2-SVP level of theory in DMSO.

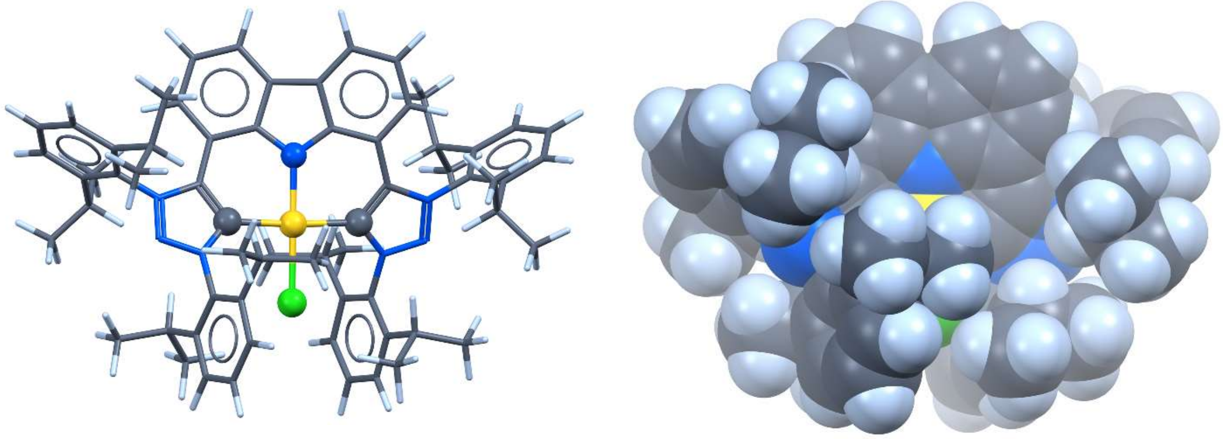

Figure S37. Views of the DFT-calculated C_2 symmetry structure of the **4b** cation illustrating distinct steric shielding of the chloride ligand in stick (left) and space-fill (right) renderings of the structure. The image also shows that only the carbazole ring system is appropriately exposed for potential π-stacking interactions with DNA bases.

1.15 Coordinates and single point energy of 4b

4b : E = -3460.469733 a.u.

**Au**	0.0001	0.1868	-0.0004	**H**	-5.9547	-1.7811	4.1418
**C**	-2.0769	0.1238	-0.1188	**H**	-4.6906	-0.5918	4.5463
**C**	-2.8717	-0.9717	0.2385	**H**	-4.3359	-1.9206	3.4124
**C**	-2.4367	-2.3305	0.5312	**C**	-6.5901	0.7392	3.0326
**C**	-3.3039	-3.3819	0.8907	**H**	-6.9143	1.3775	2.1972
**H**	-4.3369	-3.1646	1.1460	**H**	-6.1454	1.3863	3.8048
**C**	-2.8885	-4.7193	0.9009	**H**	-7.4835	0.2647	3.4688
**H**	-3.6029	-5.4948	1.1830	**C**	5.4178	-1.2673	-0.1872
**C**	-1.5912	-5.0637	0.5179	**C**	6.0992	-1.1431	-1.4113
**H**	-1.2774	-6.1092	0.4755	**C**	7.2981	-1.8593	-1.5381
**C**	-0.6921	-4.0412	0.2036	**H**	7.8614	-1.8010	-2.4713
**C**	-1.0864	-2.6729	0.2714	**C**	7.7802	-2.6438	-0.4890
**C**	1.0868	-2.6728	-0.2725	**H**	8.7164	-3.1938	-0.6106
**C**	0.6925	-4.0411	-0.2052	**C**	7.0789	-2.7302	0.7153
**C**	1.5917	-5.0635	-0.5199	**H**	7.4750	-3.3431	1.5273
**H**	1.2779	-6.1089	-0.4779	**C**	5.8722	-2.0413	0.8965
**C**	2.8890	-4.7188	-0.9028	**C**	5.5660	-0.3051	-2.5666
**H**	3.6035	-5.4942	-1.1852	**H**	4.6836	0.2488	-2.2135
**C**	3.3043	-3.3814	-0.8921	**C**	5.1068	-1.2019	-3.7302
**H**	4.3374	-3.1640	-1.1472	**H**	5.9506	-1.7800	-4.1401
**C**	2.4371	-2.3302	-0.5321	**H**	4.6887	-0.5879	-4.5435
**C**	2.8720	-0.9715	-0.2388	**H**	4.3324	-1.9156	-3.4088
**C**	2.0770	0.1238	0.1185	**C**	6.5928	0.7394	-3.0329
**C**	-2.7901	2.3608	-1.1544	**H**	6.9200	1.3771	-2.1982
**C**	-2.3422	2.4097	-2.4847	**H**	6.1481	1.3875	-3.8044
**C**	-2.2111	3.6835	-3.0567	**H**	7.4844	0.2630	-3.4708
**H**	-1.8653	3.7743	-4.0885	**C**	5.1081	-2.1307	2.2112
**C**	-2.5184	4.8336	-2.3298	**H**	4.1408	-1.6221	2.0861
**H**	-2.4138	5.8154	-2.7980	**C**	4.7960	-3.5854	2.5969
**C**	-2.9520	4.7401	-1.0056	**H**	4.2427	-4.0975	1.7946
**H**	-3.1761	5.6509	-0.4471	**H**	4.1802	-3.6093	3.5097
**C**	-3.0878	3.4953	-0.3795	**H**	5.7147	-4.1582	2.8006
**C**	-1.9940	1.1662	-3.2938	**C**	5.8641	-1.3901	3.3275
**H**	-2.1121	0.2839	-2.6493	**H**	6.8410	-1.8608	3.5237
**C**	-0.5240	1.1862	-3.7476	**H**	5.2820	-1.4090	4.2621
**H**	-0.3323	2.0079	-4.4559	**H**	6.0410	-0.3383	3.0540
**H**	-0.2690	0.2413	-4.2532	**C**	2.7898	2.3606	1.1551
**H**	0.1551	1.3143	-2.8912	**C**	3.0879	3.4951	0.3805
**C**	-2.9553	0.9869	-4.4802	**C**	2.9519	4.7398	1.0067
**H**	-4.0004	0.9341	-4.1394	**H**	3.1763	5.6507	0.4484
**H**	-2.7258	0.0555	-5.0223	**C**	2.5178	4.8332	2.3307
**H**	-2.8713	1.8223	-5.1940	**H**	2.4131	5.8150	2.7991
**C**	-3.5096	3.3938	1.0811	**C**	2.2099	3.6830	3.0574

**H**	-3.3226	2.3640	1.4215	**H**	1.8637	3.7737	4.0890
**C**	-2.6715	4.3088	1.9885	**C**	2.3412	2.4092	2.4852
**H**	-1.5968	4.1112	1.8581	**C**	3.5101	3.3937	-1.0800
**H**	-2.9319	4.1312	3.0440	**H**	3.3216	2.3641	-1.4207
**H**	-2.8559	5.3744	1.7787	**C**	5.0168	3.6617	-1.2319
**C**	-5.0159	3.6640	1.2337	**H**	5.2665	4.6843	-0.9052
**H**	-5.2642	4.6872	0.9075	**H**	5.3256	3.5529	-2.2835
**H**	-5.3245	3.5553	2.2853	**H**	5.6049	2.9553	-0.6267
**H**	-5.6052	2.9587	0.6284	**C**	2.6737	4.3101	-1.9874
**C**	-5.4175	-1.2676	0.1877	**H**	1.5987	4.1140	-1.8576
**C**	-5.8726	-2.0405	-0.8966	**H**	2.9343	4.1323	-3.0429
**C**	-7.0792	-2.7294	-0.7155	**H**	2.8596	5.3753	-1.7774
**H**	-7.4757	-3.3415	-1.5279	**C**	1.9924	1.1656	3.2939
**C**	-7.7800	-2.6441	0.4892	**H**	2.1106	0.2835	2.6492
**H**	-8.7162	-3.1941	0.6106	**C**	2.9533	0.9858	4.4806
**C**	-7.2973	-1.8608	1.5388	**H**	3.9985	0.9328	4.1400
**H**	-7.8602	-1.8033	2.4723	**H**	2.7234	0.0544	5.0224
**C**	-6.0984	-1.1447	1.4123	**H**	2.8694	1.8212	5.1944
**C**	-5.1089	-2.1289	-2.2116	**C**	0.5223	1.1859	3.7472
**H**	-4.1418	-1.6200	-2.0866	**H**	0.3306	2.0075	4.4556
**C**	-4.7963	-3.5833	-2.5979	**H**	0.2670	0.2410	4.2526
**H**	-4.2424	-4.0955	-1.7961	**H**	-0.1565	1.3143	2.8906
**H**	-4.1809	-3.6067	-3.5111	**Cl**	0.0003	2.5246	-0.0010
**H**	-5.7149	-4.1564	-2.8013	**N**	-2.9830	1.0614	-0.5466
**C**	-5.8656	-1.3883	-3.3274	**N**	-4.2321	0.6595	-0.4585
**H**	-6.8423	-1.8593	-3.5234	**N**	-4.1698	-0.5589	0.0261
**H**	-5.2838	-1.4066	-4.2622	**N**	0.0002	-1.8574	-0.0004
**H**	-6.0429	-0.3366	-3.0534	**N**	4.1700	-0.5588	-0.0256
**C**	-5.5647	-0.3077	2.5682	**N**	4.2320	0.6594	0.4596
**H**	-4.6806	0.2441	2.2162	**N**	2.9829	1.0613	0.5471
**C**	-5.1091	-1.2051	3.7326				

1.16 Stability studies with GSH

The stability of **4b** in the presence of GSH was evaluated with ^{1}H NMR. A solution of GSH (100 mM, D_2O) was added to an NMR sample of **4b** (10 mg, DMSO-d_6) to obtain a final GSH concentration of 20 mM in the sample. The ^{1}H NMR spectra were measured at certain time intervals for 48 hrs. After 48 hrs, 10 µL of the NMR sample was added to 990 µL acetonitrile (HPLC grade) for MS analysis. The interaction of GSH with **4b** as a function of time was studied by UV-vis spectroscopy at a molar ratio of 1:7 (**4b**:GSH) in DMSO (5% V/V H_2O). The reaction was monitored for 24 hours at 15 min intervals for the first 3 hours, 30-minute intervals until 10 hours, and hourly until 24 hours.

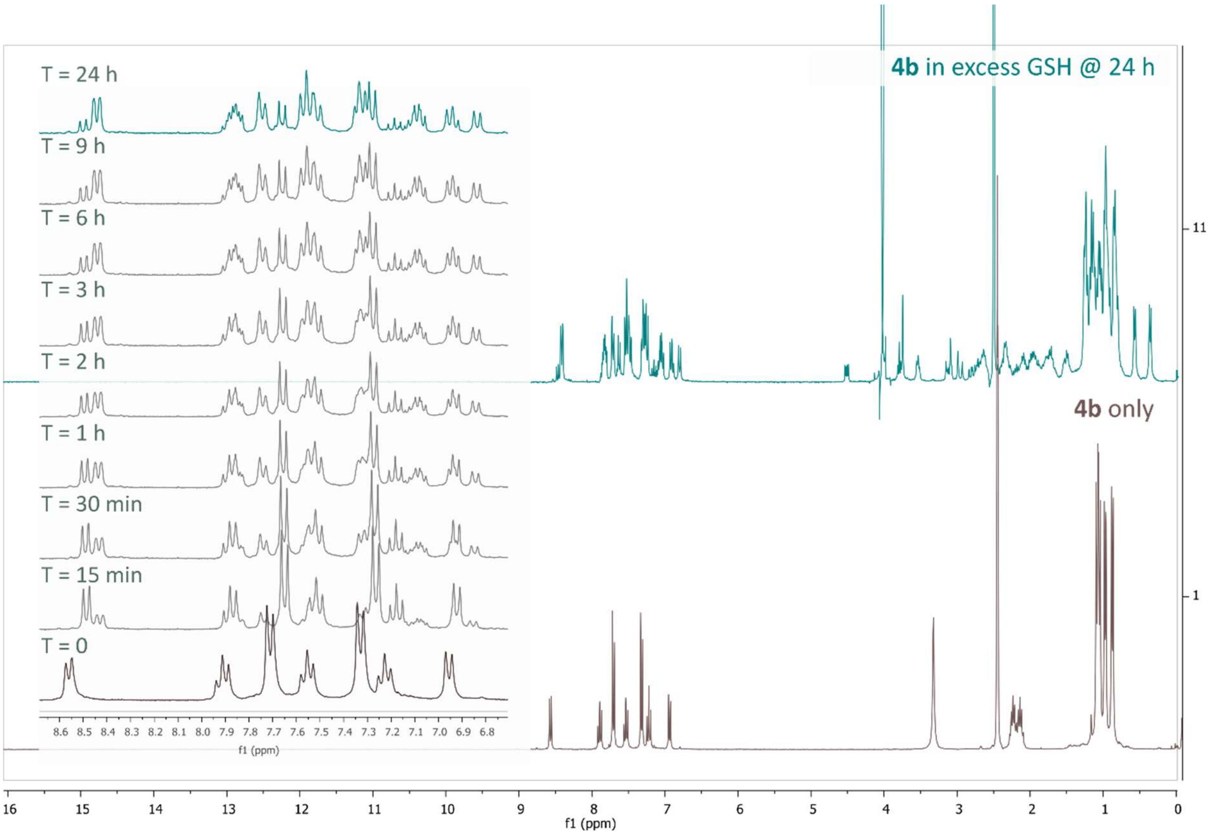

Figure S38. The ^{1}H NMR spectra of **4b** in the presence of excess GSH at T = 24 hr (top, blue) and **4b** before addition of GSH for comparison (bottom spectrum, brown) in $(CD_3)_2SO$ at room temperature. The inset magnifies the changes in the aromatic region upon the addition of GSH at certain time intervals.

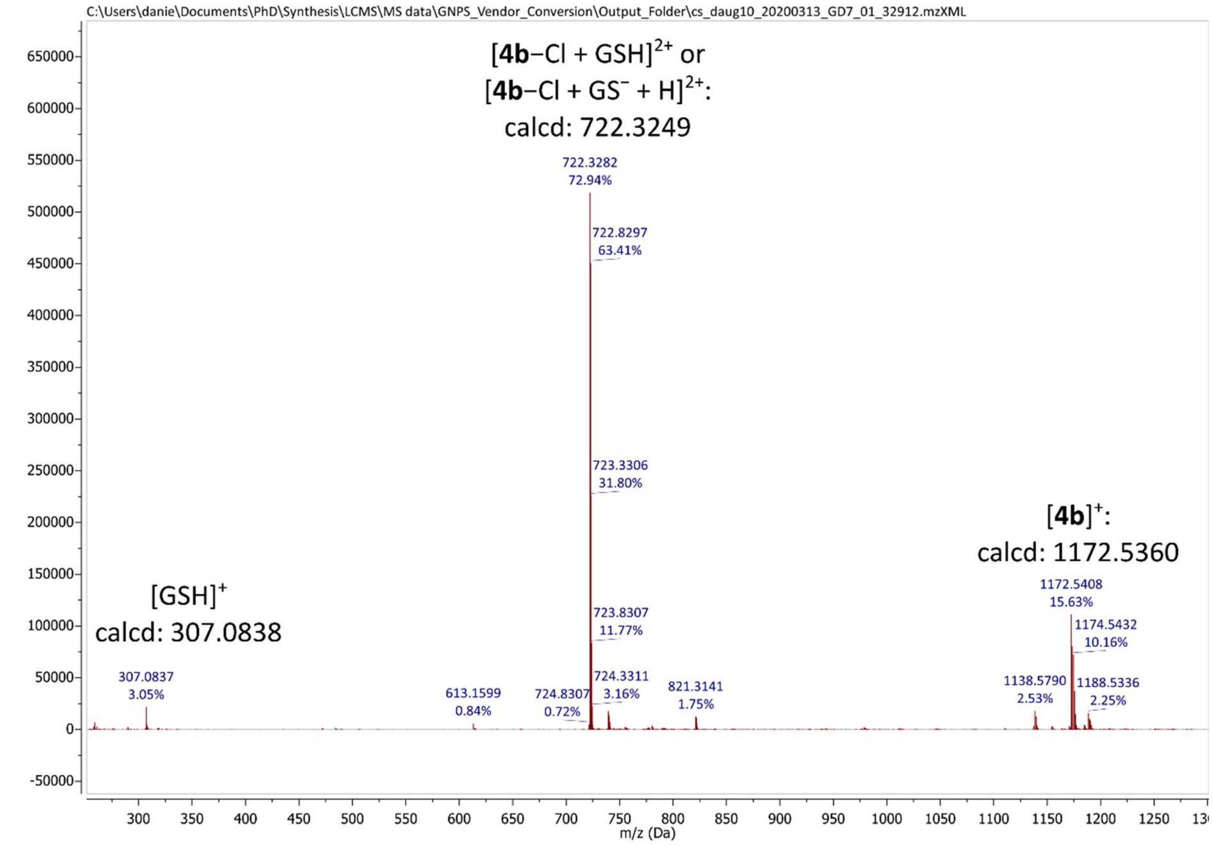

Figure S39. The ESI-MS spectrum of **4b** in the presence of excess GSH after 48 hours.

1.17 UV-vis spectroscopy

The absorption titration studies were performed at 28 °C (± 2 °C) in 1x PBS (0.01 M, phosphate buffered saline solution) and 10% (V/V) DMSO. Samples were prepared with a fixed concentration of **4b** (3.08×10^{-5} – 3.16×10^{-5} M) to which incremental amounts (5–50 µL) of ctDNA (0.0014 M bp^{-1}, ε_{260} = 13 200 bp M^{-1} cm^{-1}) were added until 1/3 ratio of [**4b**]/[ctDNA] was obtained. The samples were incubated for 10 minutes prior to the measurement of the absorption spectrum. Experiments were repeated in triplicate. Melting curves were recorded in 1x PBS (10% V/V DMSO) at a ctDNA concentration of 75 µM bp^{-1}. The melting profile of ctDNA in the presence of EB and **4b** (both at 35 µM) were measured on a Jasco J-1500 CD spectrometer. The change in absorbance at 260 nm was monitored as samples were continuously heated at a rate of 2 °C/min in 2 °C intervals over a temperature range of 40–105 °C.

1.18 Electrophoretic mobility studies

Stock solutions of each test compound were prepared in DMSO. For **4b** the concentrations were 1000, 500, 100, 50, 10, 5 and 1 µM. From the stock solutions, 1 µL was added to 1 µL pUC57 in 1 µL 10× TAE (40 mM Tris-acetate and 1 mM EDTA, pH 8.3) and 7 µL Type 1 ultrapure water. These samples were incubated at 37 °C for 30 minutes before each successive EMSA experiment. Meanwhile, 0.437 g of agarose gel was added to 37 mL 1× TAE and brought to the boil. The gel solution was cooled down 60 °C, poured into the electrophoretic mould and allowed to set for at least 30 minutes. After incubation, 2 µL of loading dye (bromophenol blue and xylene cyanol FF) was added and 6 µL of each sample was loaded into a well. The gels were placed in the Mini-Sub-Cell GT® Agarose Gel Electrophoresis System (Bio-Rad) with 1× TAE and ran for 120 minutes at 65 mV. After the run was completed, the gels were stained with ethidium bromide (5 mg/L) for at least 30 minutes and de-stained with Type 1 ultrapure water for at least 10 minutes. The gels were visualized using a G:Box Chemi XRQ gel doc system (Syngene) with mid-wave UV transillumination and a UV filter (GeneSys 1.4.6.0). Experiments were repeated at least three times.

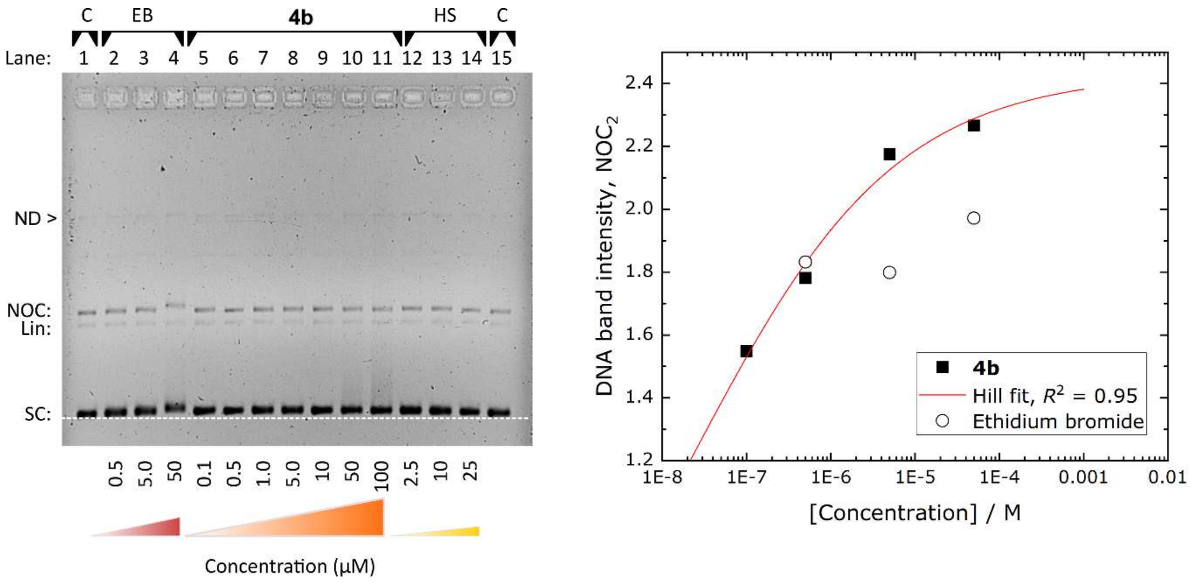

Figure S40. Left: Full EMSA gel of **4b** and with pUC57 plasmid DNA (12.5 ng/well, 1× TAE buffer (10% DMSO) that have super-coiled (SC), nicked-open circular (NOC) and linear (Lin) DNA. The lanes contain pUC57 DNA only (C, lanes and 15), increasing concentrations of ethidium bromide (EB, lanes 2–4), **4b** (lanes 5–11) and Hoechst 33258 (HS, lanes 12–14). Experiments were done in triplicate and the representative assay of each is shown. Right: densitometry analysis of the DNA concentration present in the high MW NOC dimer bands (ND) for EB in lanes 2–4 and **4b** in lanes 5–11. The data suggest that the gold(III) complex facilitates dimerization of NOC pUC57 plasmid DNA in a dose-dependent manner.

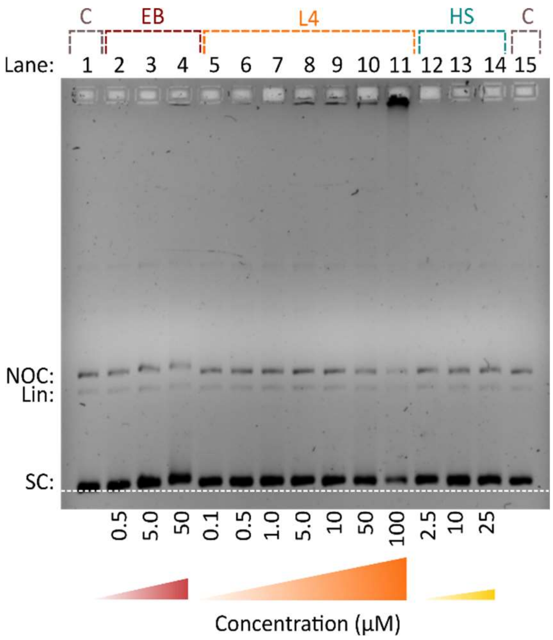

Figure S41. EMSA of **L4** with pUC57 plasmid DNA (12.5 ng/well, 1x TAE buffer, 5 % DMSO) that have super-coiled (SC), nicked-open circular (NOC) and linear (Lin) DNA. The lanes contain pUC57 DNA only (C, lanes and 15), increasing concentrations of ethidium bromide (EB, lanes 2–4), **L4** (lanes 5–11) and Hoechst 33258 (HS, lanes 12–14). Experiments were done in triplicate and the representative assay of each is shown.

From the electrophoresis gel of **L4** and pUC57 plasmid DNA, precipitation of the DNA is evident in wells at higher compound concentrations (lanes 8–11). Excessive precipitation in the well of lane 11 (**L4** concentration = 100 µM) prevented the migration of most of the DNA. The precipitation of DNA in the presence of **L4** in buffered solutions was particularly problematic in subsequent mechanistic studies such as UV-vis titrations, DNA melt curve analysis, and viscometry; unambiguous results with clean fits to binding isotherm models were not obtained.

1.19 Viscosity measurements

The change in viscosity of a buffered solution of ctDNA was evaluated as a function of the concentration of **4b**, ethidium bromide (EB), and Hoechst 33258 (HS) added to a fixed concentration of sheared ctDNA at 37 °C in 1x PBS (10% V/V DMSO). Compounds were incubated with the DNA target for 30–60 min at 37 °C prior to performing viscosity measurements. The ctDNA concentrations were typically 1.0, 5.0, 50, or 100 μM for these experiments, which were performed in a PCTFE capillary with a diameter of 1.62 mm containing 100 μL aliquots of solution in a rolling ball Lovis 2000 M microviscometer (Anton Paar) at 37 °C. All measurements were performed with a 1.5 mm diameter gold-coated steel ball (4 μm thick layer) in triplicate (the minimum number of repetitions). After recording the dynamic viscosity (mPa s) of ctDNA alone in the DMSO-buffer solution (η_0), the dynamic viscosity for each analyte-ctDNA mixture (η) was measured. Analyte compound concentrations ranged from 0.50 μM to 1000 μM, depending on solubility and whether DNA precipitation was evident or not. Hoechst 33258, for example, is limited to a maximum concentration of ~ 30 μM in the buffer system used with DNA precipitation occurring at higher concentrations. Data from triplicate measurements were averaged and used to calculate a dimensionless relative viscosity for the solution, $\{(\eta - \eta_0)/\eta_0\}^{(1/3)}$, which was plotted as the relative change in viscosity, $\Delta\{(\eta - \eta_0)/\eta_0\}^{(1/3)}$, against *r*, where *r* is the analyte to ctDNA base pair mole ratio (i.e., [analyte]/[ctDNA base pairs]).

1.20 CD and LD spectra

LD and CD spectra were recorded concurrently (two detection channels) on a Jasco J-1500 CD spectrometer at 37 °C using a thermostatted cylindrical quartz Couette cell accessory in the range of 200–650 nm at a rotation rate of 900 rpm. The concentration of ctDNA in 1x PBS solution (10% V/V DMSO) was kept constant at 10 μM bp^{-1}, while varying the molar concentration ratio (*r*) of **4b** relative to ctDNA to give solutions with *r* = 0.25, 0.5, 1.0, 2.0 and 4.0. The CD spectra were also measured separately using a 10 mm pathlength quartz semi-micro cuvette with the same solutions to confirm the CD measurements from the Couette cell.

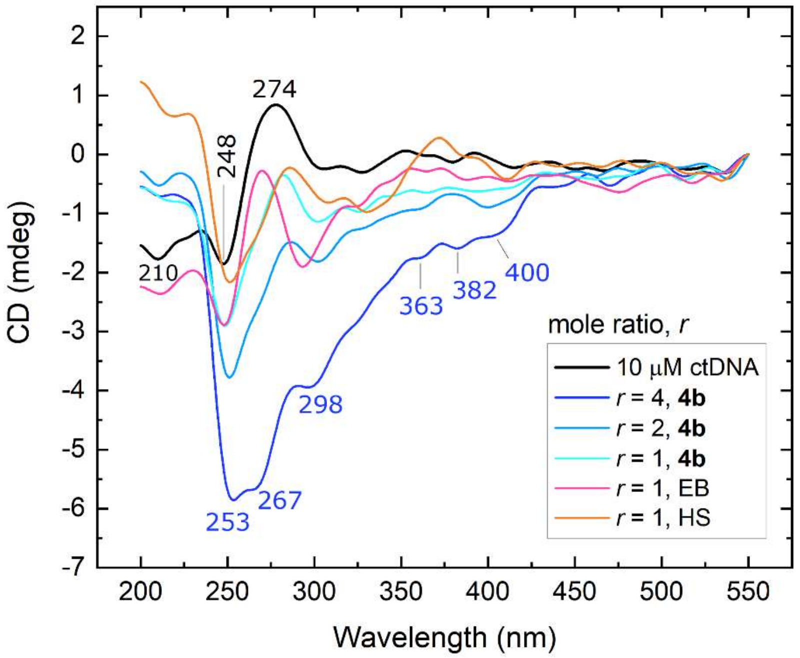

Figure S42. Circular dichroism (CD) spectra delineating the interaction of **4b** with ctDNA. All spectra are smoothed (8-point fast Fourier transform). CD spectra recorded as a function of the mole ratio *r* of the gold complex to ctDNA base pairs (10 μM throughout) in PBS-DMSO (10% V/V) buffer at 37 °C are shown. Spectra for ethidium bromide (EB) and Hoechst 33258 (HS) are included at *r* = 1 to exemplify the induced CD (ICD) spectra from a standard intercalator (EB) and minor groove binder (HS). The ICD spectrum for the achiral gold complex, **4b**, is especially pronounced when *r* = 4 and the binding equilibrium is closer to saturation. The spectra were recorded in CD mode within a microvolume Couette flow cell to orient (align) the DNA target along the flow direction (axis).

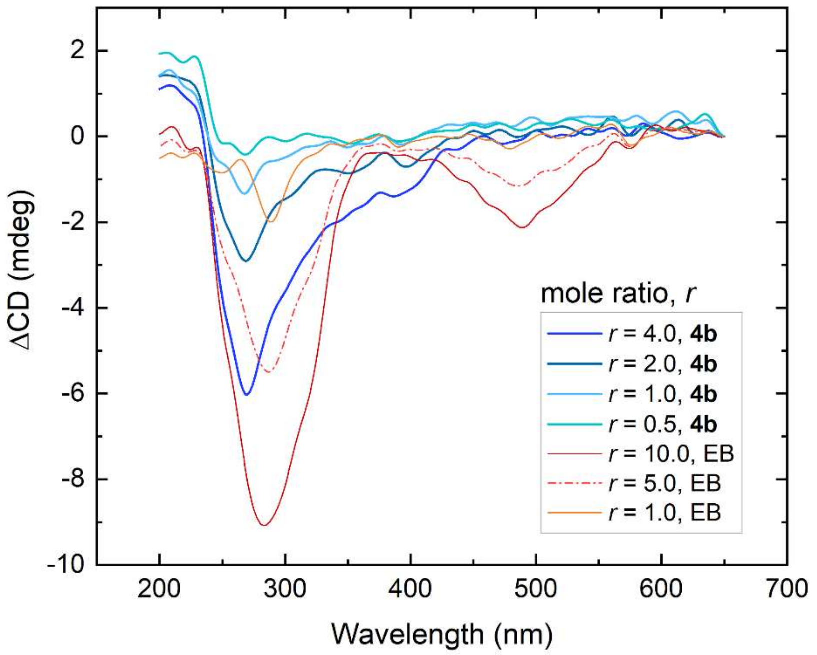

Figure S43. Difference circular dichroism (ΔCD) spectra delineating the interaction of **4b** with ctDNA. All spectra are smoothed (8-point fast Fourier transform) and were produced by subtracting the CD spectrum of ctDNA from the data. ΔCD spectra recorded as a function of the mole ratio *r* of the gold complex to ctDNA base pairs (10 μM throughout) in PBS-DMSO (10% V/V) buffer at 37 °C are shown. Spectra for ethidium bromide (EB) are included at several mole ratios to exemplify the induced CD (ICD) spectra from a standard DNA intercalator. The spectra were recorded in CD mode within a microvolume Couette flow cell to orient (align) the DNA target along the flow direction (axis).

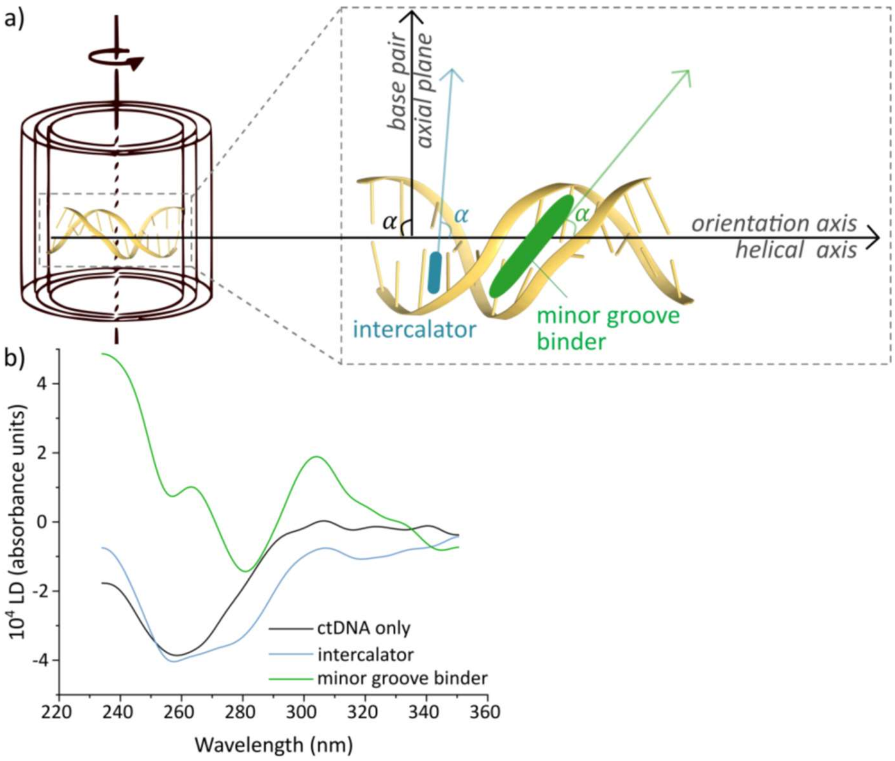

Figure S44. (a) A schematic illustration of a Couette cell (used for linear dichroism, LD, spectroscopy) aligning DNA molecules so that the orientation axis is equivalent to the helical axis of the DNA and the plane of the base pairs are orientated ~90° relative to the orientation axes. (b) The LD spectrum of ctDNA alone (black) is negative (peak at ~260 nm) due to the perpendicular orientation of the base pair plane and the helical axis. In the presence of the intercalator (EB, [compound]:[ctDNA] = 1), the LD spectrum of the system is of the same sign and magnitude as native ctDNA (i.e., negative). In the presence of a minor groove binder Hoechst 33258 (HS, [compound]:[ctDNA] = 1), the LD spectrum of the system is positive.

Comment on Figure S44. With the above method, the DNA helix axis is oriented colinear with the shear flow axis at sufficiently high rotational velocities in the microvolume Couette cell.[43] Planar aromatic DNA intercalators such as ethidium bromide (EB) bound within the base stack will therefore have their molecular plane oriented perpendicular to the DNA helix and shear flow axis.[44] Transition dipoles polarized in-plane ($\pi \rightarrow \pi^*$) lie perpendicular to the flow axis and are excited by the perpendicular ($\perp$), but not the parallel (||), linearly polarized incident light beam. By definition LD = $A_{||} - A_{\perp}$,[45] so absorption bands due to these transitions give a negative LD signal, which permits straightforward assignment of intercalative binding for planar aromatic ligands. In contrast, minor groove binders such as Hoechst 33258 (HS) or DAPI (4',6-diamidinophenyl-2-indole) have their in-plane $\pi \rightarrow \pi^*$ transition dipole moments lying colinear with the minor groove axis and thus at ~45° relative to the DNA and shear flow axes.[44,46] This leads to greater absorption of the parallel linearly polarized incident light beam such that $A_{||} > A_{\perp}$; minor groove binders are thus distinguished by positive bands in the flow LD spectrum.

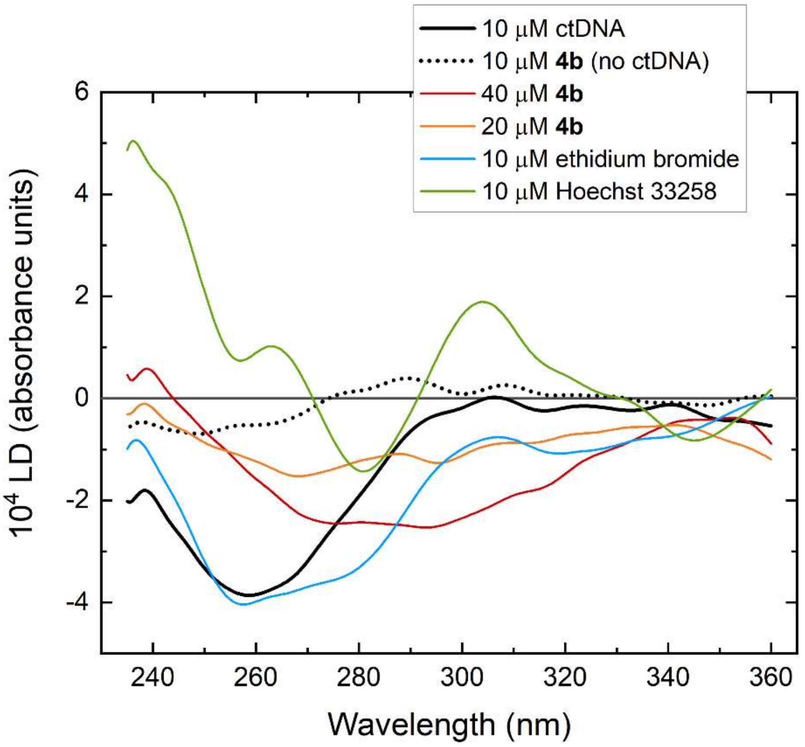

Figure S45. Linear dichroism (LD) spectra delineating the interaction of **4b** with ctDNA. All spectra are smoothed (8-point fast Fourier transform) and were recorded in PBS-DMSO (10% V/V) buffer at 37 °C. The spectrum for ethidium bromide (EB) exemplifies the induced LD (ILD) spectrum for the chromophore and is representative of the negative intensity $\pi \rightarrow \pi^*$ bands expected from a standard DNA intercalator. The spectra were recorded in LD mode within a microvolume Couette flow cell to orient (align) the DNA target along the flow direction (axis).

1.21 Macromolecular simulations (docking with *Glide*)

Docking studies were carried out with Schrödinger 2020-3 using *Glide* 5.0 SP.[47,48] Grid files for DNA targets were first created from the X-ray structures of interest downloaded from the RCSB PDB (Table S2, ESI). Macromolecules were prepared for simulations by calculating protonation and metal charge states for pH 7.0 ± 2.0 with Epik,[49,50] assigning bond orders automatically, and refining all added H atom positions. Crystal mates were used to create complete macromolecular assemblies for simulations. Grid files for docking were produced using standard settings and box sizes for ligand location ranging from 12 Å^3 (ordinary DNA intercalation sites) to 40 Å^3 (DNA four-way junctions). Large target site boxes were employed for DNA four-way junctions since experimental information on how and where compounds target these DNA forms is limited, so a large box size permits sampling of all regions within the structure for potential ligand receptor sites. Docking experiments employed *Glide* 5.0 using a small library of metal chelates with geometries based on the DFT-calculated structure of **4b** and free ligand structures calculated with the OPLS3e force field.[51] The docking runs

employed flexible ligand torsion angles, the OPLS2005 force field for ligand pose refinement, no torsion angle bias, a dielectric constant of 2.0 Debye, and no additional restrictions (such as heightened planarization of π-conjugated ring systems) or enhancements (such as including aromatic CH and halogen H-bond donors and acceptors, respectively). The same strategy was used throughout for each macromolecular target. Initial tests with several combinations of parameters showed that the default settings in Schrödinger for *Glide* were reliable, though we elected to remove any torsion angle biases for the screening procedure. (Docking scores for poses, and thus ligand binding orders, were very sensitive to the selection of parameters used for the screens, requiring a careful choice and fixed set of parameters for all runs to enable screening uniformity and comparison of data obtained from different runs and DNA targets.)

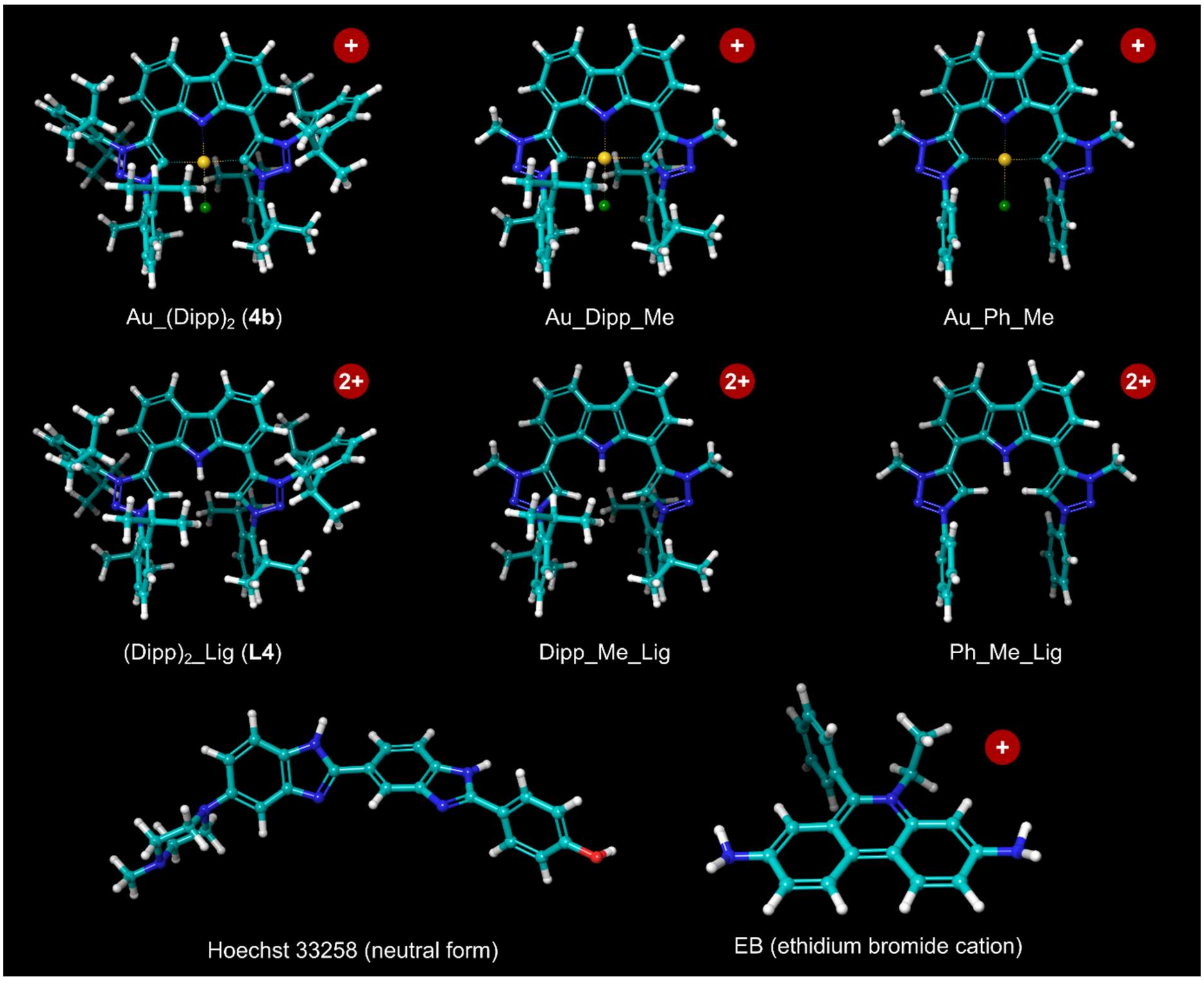

Figure S46. Molecular structures and charges of the ligands and gold(III) complexes used for docking studies with *Glide*. Only the neutral form of Hoechst 33258 is shown; docking experiments were carried out with this ligand in its neutral form as well as different tautomer and ionization states at pH 7.0. The compound names relate specifically to the three-dimensional "ligand" coordinate files used for the *in silico* study with *Glide*. Au_(Dipp)$_2$ and (Dipp)$_2$_Lig are named **4b** and **L4** in the main paper.

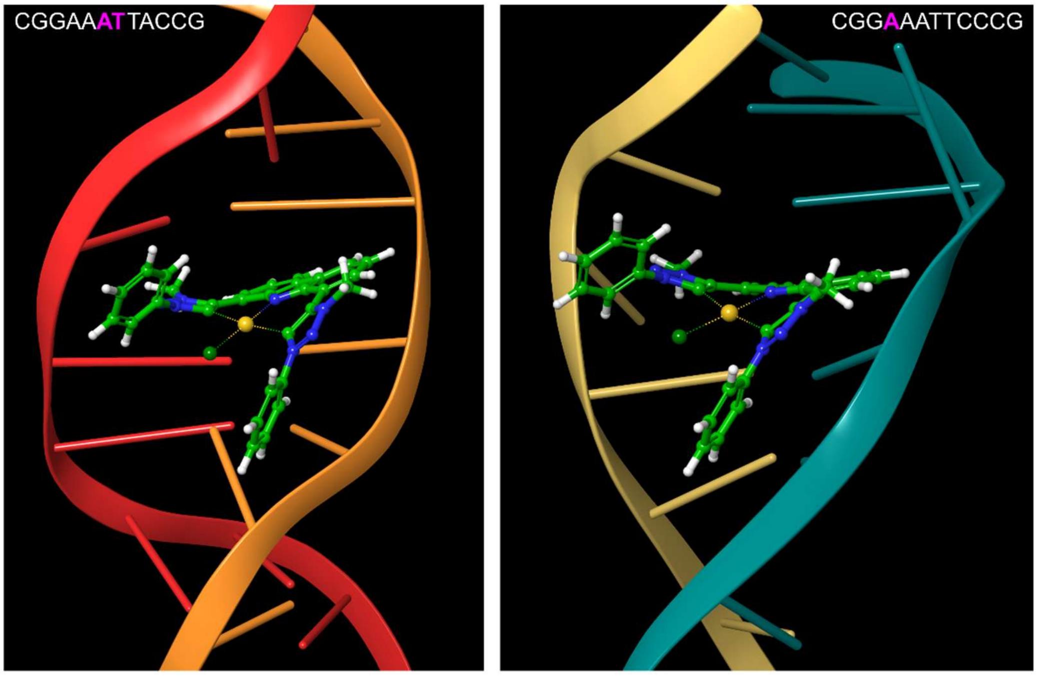

4E1U: TA intercalation (Au_Me_Ph) **2O1I: AA mismatch insertion (Au_Me_Ph)**

Figure S47. Structures of the lowest energy poses for Au_Me_Ph docked into DNA targets with typical intercalation and insertion sites for metal chelates. The X-ray structures have the PDB codes 4E1U and 2O1I. The glide docking score for the intercalation adduct with 4E1U is –10.21 kcal/mol while that for 2O1I is –12.09 kcal/mol. Ethidium bromide gave docking scores of –8.77 kcal/mol and –9.88 kcal/mol for the same pair of sites, respectively. All DNA atoms are omitted for clarity. Carbon atoms of the ligand are coloured bright green, the chloride ion dark green, all nitrogen atoms dark blue, and the gold(III) ion golden yellow.

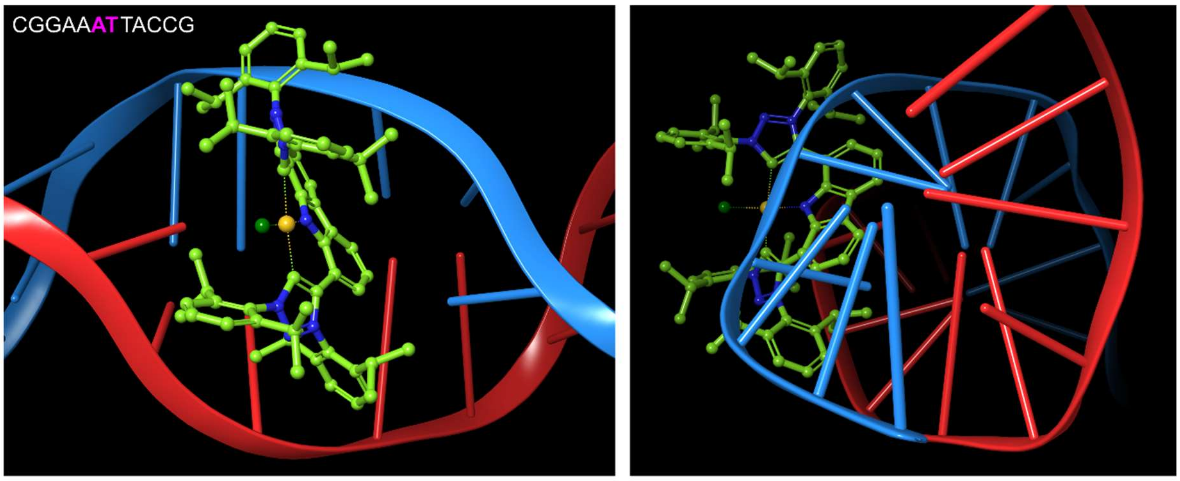

4E1U: TA intercalation (4b)

Figure S48. Structure of the lowest energy pose for **4b** bound at a 5′-TA-3′ step in the oligonucleotide with the RCSB PDB code 4E1U. The Glide docking score (ΔG_{bind}) for the pose was –7.93 kcal/mol. The view on the left shows that the carbazole ring is canted in the intercalation binding pocket and that the entry is via the minor groove. The view on the right taken approximately down the helix axis shows the imperfect intercalation of the carbazole ring along with the positioning of the bulky Dipp substituents. Two Dipp groups extend into the solvent region beyond the DNA fibre while the two groups lower down in this view of the structure lie within the minor groove. Hydrogen atoms and DNA atoms are omitted for clarity. Carbon atoms of the ligand are coloured light green, the chloride ion dark green, all nitrogen atoms dark blue, and the gold(III) ion golden yellow.

Table S2 Assessment of DNA targets for Au(III) CNC pincer complexes, their free ligand counterparts, and two control compounds (EB and Hoechst 33258). *Glide* 5.0 was used to perform the docking study in Schrodinger 2020-3. All docking scores are effective *in silico* ΔG_{bind} values (kcal/mol) for the pose with the highest affinity for the target site shown in the DNA sequence (bold underlined bases). Tight grids for intercalation binding sites were generated using 10 or 12 $Å^3$ target boxes, while loose grids used 35-40 $Å^3$ boxes (effectively the entire DNA substrate).

DNA Target	PDB Code	Sequence (5′ to 3′)	Au_(Dipp)2 (4b)	(Dipp)2 Lig (L4)	Au_Dipp_Me	Dipp_Me Lig	Au_Ph_Me	Ph_Me Lig	EB	HS 33258
**4-way junction**	**1JUC**	CCGG**TA**CCGG	0.000	–7.218	0.000	–8.949	–8.521	–8.369	–7.490	–7.156
	**1M6G**	TCGG**TA**CCGA	–6.809	–5.255	0.000	–8.205	–8.673	–9.826	–8.767	–8.609
	**2GWA**	TCGG**TA**CCGA	0.000	–5.262	0.168	–10.469	–10.713	–12.286	–8.222	–7.156
	**3Q5C**	CGGC**GG**CCGC	**–7.412**	–8.093	0.000	–8.197	–7.619	–9.187	–6.738	–6.528
**3–way junction**	**3I1D (tight grid 1)**	CG**TA**CG	–10.839	–9.614	–3.996	–9.034	–8.965	–9.913	–7.676	–7.052
	**3I1D (tight grid 2)**	CG**TA**CG	**–11.888**	0.000	–3.996	0.000	–8.965	–9.913	–7.723	0.000
	**2ET0**	CG**TA**CG	–8.363	–8.854	–0.450	–9.672	–6.852	–10.632	–8.598	–6.842
	**3FX8**	CG**TA**CG	0.000	0.000	0.000	–9.896	–8.753	–9.467	–8.545	0.000
	**4JIY (RNA)**	CG**UA**CG	–9.619	–9.950	–2.470	–9.677	–10.477	–9.707	–7.738	–7.226
**dsDNA intercalation**	**2O1I**	CGGAA**AT**TCCCG	0.000	0.000	–0.805	0.000	0.000	–10.812	–10.524	–7.431
	**3GSJ**	CGGAA**AT**TACCG	0.000	0.000	0.000	0.000	0.000	–10.975	0.000	–7.621
	**4E1U (tight grid)**	**CG**GAAATTACCG	0.000	0.000	–1.202	–1.538	0.000	–7.009	–7.958	0.000
	**4E1U (loose grid 1)**	CGGAA**AT**TACCG	**–7.933**	0.000	0.000	–8.075	0.000	–7.987	0.000	0.000
	**4E1U (loose grid 2)**	CGGAA**AT**TACCG	–7.746	0.000	0.000	–7.932	–9.888	0.000	0.000	0.000
	**4E1U (tight grid)**	CGGAA**AT**TACCG	0.000	0.000	–4.348	–8.158	–10.210	–8.718	–8.771	0.000
	**4E1U (tight grid)**	CGGAAATTAC**CG**	0.000	0.000	–0.652	–4.287	–5.951	–5.386	0.000	0.000
**dsDNA mismatch insertion**	**2O1I**	CGGAAATT**C**CCG	–6.927	–7.496	0.000	–10.118	–11.299	–11.039	–9.991	0.000
		CGG**A**AATTCCCG	–8.901	–8.170	0.000	–11.389	–12.085	–10.834	–9.883	0.000
	**3GSJ (tight grid)**	CGGAAATT**A**CCG	0.000	–6.581	0.000	–11.066	0.000	–11.644	–9.926	0.000
	**3GSJ (loose grid)**	CGG**A**AATTACCG	**–9.549**	–7.996	0.000	–10.590	0.000	–11.160	–10.070	0.000
	**3GSK (tight grid)**	CGGAAATT**A**CCG	–7.872	–7.692	0.000	–10.786	–8.565	–10.418	–10.115	0.000

DNA Target	PDB Code	Sequence (5′ to 3′)	Au_(Dipp)2 (4b)	(Dipp)2 Lig (L4)	Au_Dipp_Me	Dipp_Me Lig	Au_Ph_Me	Ph_Me Lig	EB	HS 33258
	**3GSK (loose grid)**	CGG**A**AATTACCG	–7.970	–7.948	0.000	–11.213	–11.528	–10.514	–9.844	–7.212
	**4E1U (tight grid site 2)**	CGG**A**AATTACCG	0.000	0.000	–1.173	0.000	–7.273	–8.829	–10.880	0.000
	**4E1U (tight grid site 4)**	CGGAAATT**A**CCG	0.000	0.000	0.000	–9.278	0.000	–7.959	–9.974	0.000
	**4E1U (loose grid)**	CGGAAATT**A**CCG	0	–8.185	0.000	–11.41	0.000	–8.856	0.000	–7.373
**DNA Hairpin**	**1OVF**	CCGTTTTGTGG	**–4.779**	–3.812	–0.591	–7.837	–6.113	–8.884	–8.751	–6.394
	**1LA8**	CGCGGTGTCCGCG	0	–3.769	0.000	–6.725	–8.062	–7.864	–7.327	–7.401
	**1ZHU**	CAATGCAATG	0	–3.952	–0.689	–4.272	–5.161	–4.593	–5.537	–6.295
		**Average***	**–8.329**	**–7.050**	**–1.777**	**–8.699**	**–8.784**	**–9.362**	**–8.741**	**–7.164**
		**ESD**	**1.776**	**1.964**	**1.634**	**2.469**	**1.933**	**1.798**	**1.355**	**0.578**

*The average energy was taken over all substrate binding site types excluding zero entries.

A noteworthy observation when comparing the data in the above table is that the mean Glide docking score for **4b** (–8.3 ± 1.8 kcal/mol) is slightly lower than that of EB (–8.74 ± 1.4 kcal/mol) across all DNA substrates (targets). This mirrors the experimental binding constant data reported for **4b** (2.6×10^5 M^{-1}) in the paper and EB in the literature (4.94×10^5 M^{-1})[52] with calf thymus DNA.

1.22 Predictive modelling (docking with *Chimera*)

Machine learning (ML) was used as a predictive tool to estimate the affinity of **4b** bound to various DNA configurations. All DNA-containing structures were downloaded from the www.rcsb.org database (October 2020) and were prepared for docking with Chimera[53] by removing ions, ligands, proteins and water molecules and adding hydrogens. The X-ray structure geometry of **4b** was used for docking the complex into >7000 DNA structures. Autodocktools[54] was used to convert the receptors and "ligand" to .pdbqt format and QuickVina-W[55] was used to dock **4b** into the prepared DNA structures. A previously developed Random Tree Forest model[56] to predict the activity of newly synthesized beta-secretase 1 inhibitors was used to predict the activity of the docked **4b** complexes. The Smina package[57] was used to extract 43 descriptors of the ligand-receptor complex and Babel[58] was used to calculate 9 ligand descriptors that were used in the RTF model. The Ranger package in R was used to predict the Gibbs free energy (ΔG) of the docked poses and this value was converted the predicted K_i-value with the standard Gibbs function (predicted K_i = $e^{(-\Delta G/RT)}$).

After docking **4b** into > 7000 DNA structures, extracting the data from the docked poses and applying the RTF model to predict the Gibbs free energy of the complex, only 12 complexes were predicted to have a K_i of less than 10 µM. Interestingly 11 out of the 12 complexes had **4b** bound to the *Z*-DNA configurations (Table S3 and Figure S49). The configuration of **4b** bound to 314D is similar for all **4b** docked into the *Z*-DNA structures (Table S3). The Au^{III} ion of the cationic complex is sandwiched between oxygen atoms of the phosphate backbone (3.319 Å and 4.507 Å for the closest oxygens) and the dicarbazolide moiety is nestled within the DNA groove (Figure S49).

The formation of *Z*-DNA in cells is tightly regulated and plays important roles immunological responses, gene expression, and genome stability.[59] Interestingly, *Z*-DNA formation is associated with control of *c-myc* expression.[60] The proto-oncogene *c-myc is* overexpressed in 40% of breast cancers and the overexpression of *c-myc* in triple negative breast cancers (TNBC) correlates with poor prognosis.[61–64] Selective inhibition of c-myc expression in TNBC offers a valid therapeutic avenue for these cancers. The observed selectivity of **4b** towards TNBCs cancer in this study can, in part, be explained by **4b** binding to *Z*-DNA structures and subsequently interfering with c-myc expression. However, future studies are needed to confirm this.

Table S3: The PDB structures for **4b** predicted to have a K_i < 10 µM.

PDB Code	Predicted K_i (µM)	Type of DNA configuration
**1DN4**	2.46	*Z*-DNA
**1VTV**	2.79	*Z*-DNA
**314D**	3.73	*Z*-DNA
**293D**	3.97	*Z*-DNA
**312D**	4.38	*Z*-DNA
**313D**	4.50	*Z*-DNA
**331D**	4.59	*Z*-DNA
**1VTY**	4.71	*Z*-DNA
**1D76**	4.90	*Z*-DNA
**1M6R**	5.44	*Z*-DNA
**1ZEY**	5.98	CGG *A*-DNA
**2DCG**	6.54	*Z*-DNA

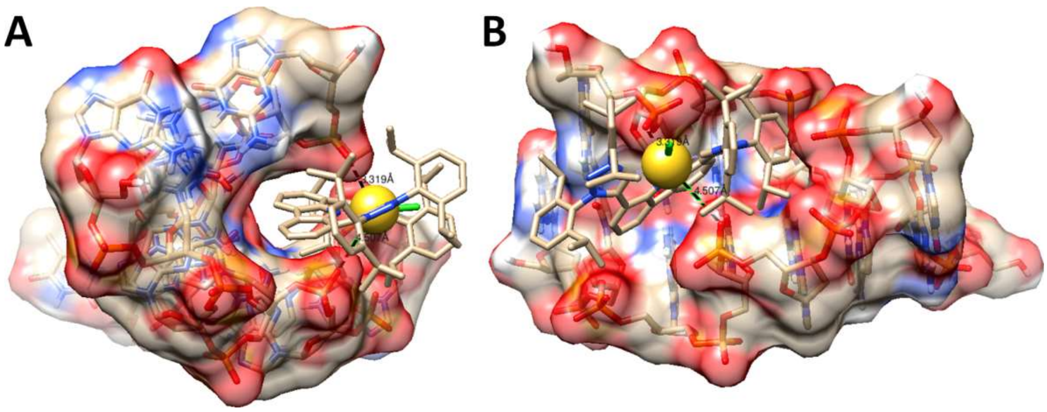

Figure S49. Docking pose of **4b** in the *Z*-DNA configuration of DNA (314D). The dicarbazolide moiety is nestled within the DNA groove and the Au^{III} ion of the cationic complex is sandwiched between oxygen atoms of the phosphate backbone.

References

[1] Y. Liu, M. Nishiura, Y. Wang, Z. Hou, *Journal of the American Chemical Society* **2006**, *128*, 5592–5593.

[2] V. C. Gibson, S. K. Spitzmesser, A. J. P. White, D. J. Williams, *Dalton Transactions* **2003**, 2718–2727.

[3] M. S. Bennington, H. L. C. C. Feltham, Z. J. Buxton, N. G. White, S. Brooker, *Dalton Transactions* **2017**, *46*, 4696–4710.

[4] S. J. Malthus, S. A. Cameron, S. Brooker, *Inorganic chemistry* **2018**, *57*, 2480–2488.

[5] N. Nimitsiriwat, V. C. Gibson, E. L. Marshall, P. Takolpuckdee, A. K. Tomov, A. J. P. White, D. J. Williams, M. R. J. Elsegood, S. H. Dale, *Inorganic Chemistry* **2007**, *46*, 9988–9997.

[6] A. G. M. Barrett, M. R. Crimmin, M. S. Hill, P. B. Hitchcock, G. Kociok-Köhn, P. A. Procopiou, *Inorganic Chemistry* **2008**, *47*, 7366–7376.

[7] M. J. Mintz, C. Walling, *Organic Syntheses* **1969**, *49*, 9.

[8] X. F. Zhao, C. Zhang, *Synthesis* **2007**, 551–557.

[9] J. R. Shapley, D. J. Santure, A. P. Sattelberger, F. A. Cotton, W. Wang, in *Inorganic Syntheses* (Ed.: H.D. Kaesz), John Wiley & Sons Ltd, **2007**, pp. 215–225.

[10] H. Meerwein, *Organic Syntheses* **1966**, *46*, 113.

[11] D. I. Bezuidenhout, G. Kleinhans, G. Guisado-Barrios, D. C. Liles, G. G. G. G. G. G. Ung, G. Bertrand, *Chemical Communications* **2014**, *50*, 2431.

[12] G. Kleinhans, M. M. Hansmann, G. Guisado-Barrios, D. C. Liles, G. Bertrand, D. I. Bezuidenhout, *Journal of the American Chemical Society* **2016**, *138*, 15873–15876.

[13] G. R. Fulmer, A. J. M. Miller, N. H. Sherden, H. E. Gottlieb, A. Nudelman, B. M. Stoltz, J. E. Bercaw, K. I. Goldberg, *Organometallics* **2010**, *29*, 2176–2179.

[14] O. V. Dolomanov, L. J. Bourhis, R. J. Gildea, J. A. K. Howard, H. Puschmann, *Journal of Applied Crystallography* **2009**, *42*, 339–341.

[15] G. M. Sheldrick, *Acta Crystallographica Section A: Foundations of Crystallography* **2015**, *71*, 3–8.

[16] G. M. Sheldrick, *Acta Crystallographica Section C Structural Chemistry* **2015**, *71*, 3–8.

[17] N. Huang, M. M. Siegel, G. H. Kruppa, F. H. Laukien, *Journal of the American Society for Mass Spectrometry* **1999**, *10*, 1166–1173.

[18] G. Guisado-Barrios, J. Bouffard, B. Donnadieu, G. Bertrand, *Organometallics* **2011**, *30*, 6017–6021.

[19] M. Tashiro, T. Yamato, *Synthesis (Germany)* **1979**, *1979*, 48–50.

[20] M. Tashiro, *Synthesis (Germany)* **1979**, *1979*, 921–936.

[21] N. Lewis, I. Morgan, *Synthetic Communications* **1988**, *18*, 1783–1793.

[22] S. A. Saleh, H. I. Tashtoush, *Tetrahedron* **1998**, *54*, 14157–14177.

[23] J. Bouffard, B. K. Keitz, R. Tonner, G. Guisado-Barrios, G. Frenking, R. H. Grubbs, G. Bertrand, *Organometallics* **2011**, *30*, 2617–2627.

[24] E. C. Keske, O. V Zenkina, R. Wang, C. M. Crudden, *Organometallics* **2012**, *31*, 456–461.

[25] J. Cai, X. Yang, K. Arumugam, C. W. Bielawski, J. L. Sessler, *Organometallics* **2011**, *30*, 5033–5037.

[26] H. Schmidbaur, A. Schier, *Chemical Society Reviews* **2012**, *41*, 370–412.

[27] C. Gabbiani, A. Casini, L. Messori, *Gold Bulletin* **2007**, *40*, 73–81.

[28] D. Canseco-Gonzalez, A. Petronilho, H. Mueller-Bunz, K. Ohmatsu, T. Ooi, M. Albrecht, *Journal of the American Chemical Society* **2013**, *135*, 13193–13203.

[29] M. Contel, J. Garrido, M. C. Gimeno, J. Jiménez, P. G. Jones, A. Laguna, M. Laguna, *Inorganica Chimica Acta* **1997**, *254*, 157–161.

[30] G. Kleinhans, A. K.-W. Chan, M.-Y. Leung, D. C. Liles, M. A. Fernandes, V. W. -W. Yam, I. Fernández, D. I. Bezuidenhout, *Chemistry – A European Journal* **2020**, *26*, 6993–6998.

[31] S. E. Burdall, A. M. Hanby, M. R. J. Lansdown, V. Speirs, *Breast Cancer Research* **2003**, *5*, 89.

[32] D. G. Altman, J. M. Bland, *BMJ* **2005**, *331*, 903.

[33] S. H. Vosko, L. Wilk, M. Nusair, *Canadian Journal of Physics* **1980**, *58*, 1200–1211.

[34] C. Lee, W. Yang, R. G. Parr, *Physical Review B* **1988**, *37*, 785–789.

[35] A. D. Becke, *The Journal of Chemical Physics* **1993**, *98*, 5648–5652.

[36] S. Grimme, J. Antony, S. Ehrlich, H. Krieg, *The Journal of Chemical Physics* **2010**, *132*, 154104.

[37] F. Weigend, R. Ahlrichs, *Physical Chemistry Chemical Physics* **2005**, *7*, 3297.

[38] M. J. Frisch, G. W. Trucks, H. B. Schlegel, G. E. Scuseria, M. A. Robb, J. R. Cheeseman, G. Scalmani, V. Barone, B. Mennucci, G. A. Petersson, H. Nakatsuji, M. Caricato, X. Li, H. P. Hratchian, A. F. Izmaylov, J. Bloino, G. Zheng, J. L. Sonnenberg, M. Hada, M. Ehara, K. Toyota, R. Fukuda, J. Hasegawa, M. Ishida, T. Nakajima, Y. Honda, O. Kitao, H. Nakai, T. Vreven, Jr. J. A. Montgomery, J. E. Peralta, F. Ogliaro, M. Bearpark, J. J. Heyd, E. Brothers, K. N. Kudin, V. N. Staroverov, T. Keith, R. Kobayashi, J. Normand, K. Raghavachari, A. Rendell, J. C. Burant, S. S. Iyengar, J. Tomasi, M. Cossi, N. Rega, J. M. Millam, M. Klene, J. E. Knox, J. B. Cross, V. Bakken, C. Adamo, J. Jaramillo, R. Gomperts, R. E. Stratmann, O. Yazyev, A. J. Austin, R. Cammi, C. Pomelli, J. W. Ochterski, R. L. Martin, K. Morokuma, V. G. Zakrzewski, G. A. Voth, P. Salvador, J. J. Dannenberg, S. Dapprich, A. D. Daniels, O. Farkas, J. B. Foresman, J. V. Ortiz, J. Cioslowski, D. J. Fox, *Gaussian, Inc., Wallingford CT,* **2013**.

[39] S. Miertuš, E. Scrocco, J. Tomasi, *Chemical Physics* **1981**, *55*, 117–129.

[40] J. L. Pascual-ahuir, E. Silla, I. Tuñon, *Journal of Computational Chemistry* **1994**, *15*, 1127–1138.

[41] C. Latouche, D. Skouteris, F. Palazzetti, V. Barone, *Journal of Chemical Theory and Computation* **2015**, *11*, 3281–3289.

[42] N. M. O'boyle, A. L. Tenderholt, K. M. Langner, *Journal of Computational Chemistry* **2008**, *29*, 839–845.

[43] R. Marrington, T. R. Dafforn, D. J. Halsall, J. I. MacDonald, M. Hicks, A. Rodger, *Analyst* **2005**, *130*, 1608–1616.

[44] B. Nordén, T. Kurucsev, *Journal of Molecular Recognition* **1994**, *7*, 141–155.

[45] B. Nordén, *Applied Spectroscopy Reviews* **1978**, *14*, 157–248.

[46] B. Norden, M. Kubista, T. Kurucsev, *Quarterly reviews of biophysics* **1992**, *25*, 51–170.

[47] R. A. Friesner, J. L. Banks, R. B. Murphy, T. A. Halgren, J. J. Klicic, D. T. Mainz, M. P. Repasky, E. H. Knoll, M. Shelley, J. K. Perry, D. E. Shaw, P. Francis, P. S. Shenkin, *Journal of Medicinal Chemistry* **2004**, *47*, 1739–1749.

[48] T. A. Halgren, R. B. Murphy, R. A. Friesner, H. S. Beard, L. L. Frye, W. T. Pollard, J. L. Banks, *Journal of Medicinal Chemistry* **2004**, *47*, 1750–1759.

[49] J. R. Greenwood, D. Calkins, A. P. Sullivan, J. C. Shelley, *Journal of Computer-Aided Molecular Design* **2010**, *24*, 591–604.

[50] J. C. Shelley, A. Cholleti, L. L. Frye, J. R. Greenwood, M. R. Timlin, M. Uchimaya, *Journal of Computer-Aided Molecular Design* **2007**, *21*, 681–691.

[51] K. Roos, C. Wu, W. Damm, M. Reboul, J. M. Stevenson, C. Lu, M. K. Dahlgren, S. Mondal, W. Chen, L. Wang, R. Abel, R. A. Friesner, E. D. Harder, *Journal of Chemical Theory and Computation* **2019**, *15*, 1863–1874.

[52] S. Satyanarayana, J. C. Dabrowiak, J. B. Chaires, *Biochemistry* **1992**, *31*, 9319–9324.

[53] G. S. Couch, D. K. Hendrix, T. E. Ferrin, *Nucleic Acids Research* **2006**, *34*, 1–5.

[54] R. Huey, G. M. Morris, *The Scripps Research Insitute, USA* **2008**, *54*, 1–16.

[55] N. M. Hassan, A. A. Alhossary, Y. Mu, C. K. Kwoh, *Scientific Reports* **2017**, *7*, 1–13.

[56] C. J. van der Westhuizen, D. G. van Greunen, W. Cordier, M. Nell, V. Steenkamp, A. Stander, J.-L. Panayides, D. L. Riley, *Arkivoc* **2020**, *2020*, 84–107.

[57] D. R. Koes, M. P. Baumgartner, C. J. Camacho, *Journal of Chemical Information and Modeling* **2013**, *53*, 1893–1904.

[58] N. M. O'Boyle, M. Banck, C. A. James, C. Morley, T. Vandermeersch, G. R. Hutchison, *Journal of Cheminformatics* **2011**, *3*, 1–14.

[59] S. Ravichandran, V. K. Subramani, K. K. Kim, *Biophysical Reviews* **2019**, 383–387.

[60] H. J. Kang, T. V. T. Le, K. Kim, J. Hur, K. K. Kim, H. J. Park, *Journal of Molecular Biology* **2014**, *426*, 2594–2604.

[61] R. Naidu, N. A. Wahab, M. Yadav, M. K. Kutty, *International journal of molecular medicine* **2002**, *9*, 189–196.

[62] J. Xu, Y. Chen, O. I. Olopade, *Genes & Cancer* **2010**, *1*, 629–640.

[63] E. Wang, A. Sorolla, P. T. Cunningham, H. M. Bogdawa, S. Beck, E. Golden, R. E. Dewhurst, L. Florez, M. N. Cruickshank, K. Hoffmann, R. M. Hopkins, J. Kim, A. J. Woo, P. M. Watt, P. Blancafort, *Oncogene* **2019**, *38*, 140–150.

[64] J. A. Mertz, A. R. Conery, B. M. Bryant, P. Sandy, S. Balasubramanian, D. A. Mele, L. Bergeron, R. J. Sims, *Proceedings of the National Academy of Sciences of the United States of America* **2011**, *108*, 16669–16674.
